# Identification of a Highly Cooperative PROTAC Degrader Targeting GTP-Loaded KRAS(On) Alleles

**DOI:** 10.1021/jacs.5c10354

**Published:** 2025-10-30

**Authors:** Vesna Vetma, Ilaria Puoti, Natalia K. Karolak, Sohini Chakraborti, Emelyne Diers, Enrico Girardi, Shakil Khan, Giorgia Kidd, Katrin G. Kropatsch, Ross Mclennan, Suzanne O’Connor, Matthias Samwer, Nicole Trainor, Claire Whitworth, Andre J. Wijaya, Jeff Y. F. Wong, David Zollman, William Farnaby, Johannes Popow, Alessio Ciulli, Peter Ettmayer, Kirsten McAulay

**Affiliations:** † Centre for Targeted Protein Degradation, School of Life Sciences, 3042 University of Dundee, 1 James Lindsay Place, DD1 5JJ Dundee, Scotland, U.K.; ‡ Division of Biological Chemistry and Drug Discovery, School of Life Sciences, James Black Centre, 3042 University of Dundee, DD1 5EH Dundee, Scotland, U.K.; § 33433 Boehringer Ingelheim RCV GmbH & Co KG, 1221 Vienna, Austria

## Abstract

Kirsten rat sarcoma viral oncogene homologue (KRAS) is a frequently mutated oncogene in multiple types of cancer and is a high priority target for oncology drug development. There are many different KRAS mutations, including mutations that favor the GTP-loaded hydrolysis-incompetent “active” state of KRAS, KRAS­(on), that can lead to tumorigenesis. However, small molecule interventions thus far have predominantly targeted single mutations of “inactive” GDP-loaded KRAS, KRAS­(off), such as KRAS^G12C^. Here, we address this gap through the development of heterobifunctional VHL-based PROTACs capable of engaging and degrading KRAS­(on), thus addressing a wider range of KRAS mutations. By studying ternary complex affinity, stability, and binding modes using SPR and X-ray cocrystal structures, we identified PROTACs that exhibit high positive cooperativity in forming ternary complexes with VHL and GCP-loaded KRAS as representative of KRAS­(on) variants. Degrader activity profiling in relevant cancer cells supported the discovery of ACBI4, a PROTAC which forms a highly stable and cooperative ternary complex between VHL and GTP-bound KRAS and which potently degrades KRAS^G12R^, leading to antiproliferative effect in KRAS mutant-driven cancer cells. ACBI4 provides a new chemical tool for studying the impact of degrading KRAS­(on) mutants, which is not possible with current pan-KRAS inhibitors or degraders.

## Graphical abstract

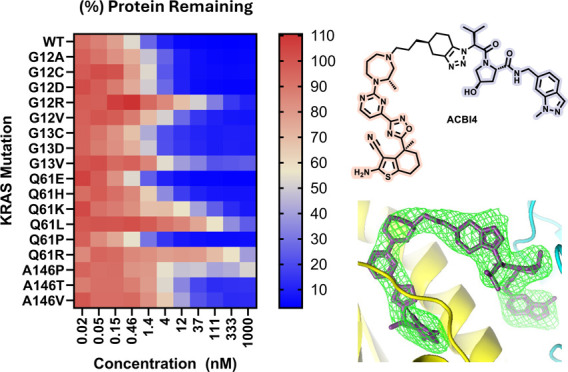

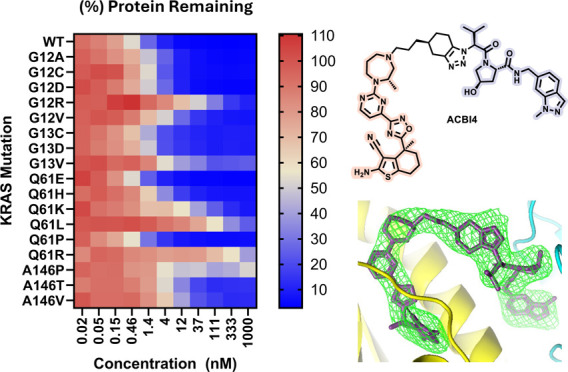

## Introduction

The mitogen-activated protein kinase (MAPK) pathway regulates several critical cellular functions involved in cell cycle control, including proliferation, differentiation, and survival. Thus, gain-of-function pathway mutations can cause unregulated cellular division. Kirsten rat sarcoma viral oncogene homologue (KRAS), a key component of the pathway, is the most commonly mutated oncogene and is a key driver of tumor growth in several human cancers.^1^ KRAS cycles between an inactive guanosine diphosphate (GDP)-loaded and active guanosine triphosphate (GTP)-loaded state, acting as a molecular switch for downstream signal transduction.^2^ The most prevalent mutations that enhance RAF-MEK-ERK signaling occur at Glycine12 (G12) and Glycine13 (G13) in the P-loop or Glutamine61 (Q61) in the Switch II region and impair GTPase activity, leading to abnormal signaling. Substitutions at G12 and G13 generally reduce GAP (GTPase-activating protein) binding, while Q61 mutations damage the inherent GTPase function of KRAS.^3^ Studies suggest that KRAS mutations are most prevalent in colorectal (CRC), pancreatic (PDAC), and non-squamous non-small cell lung (NSCLC) cancers, with KRAS G12D (29%), G12V (23%), G12C (15%), G13D (7%), and G12R (5%), the five most commonly observed isoforms, accounting for ∼80% of all modifications.^4^

Despite the high level of interest in KRAS as a therapeutic target, so far only covalent inhibitors targeting the G12C allele have been clinically approved.^5,6^ However, these agents are only effective for a small percentage of KRAS-driven cancers, and so there remains a need for clinically proven therapeutics that can address other, and ideally multiple, oncogenic KRAS alleles. Indeed, most of the disclosed efforts seeking to inhibit oncogenic KRAS variants have relied on specific interactions with a single mutation. In addition to covalent inhibitors targeting G12C, reversible inhibitors containing basic groups have been utilized to selectively target the G12D aspartate residue.^7^ Recently, the first KRAS inhibitor, BI-2865 (Figure 1), capable of engaging multiple KRAS alleles was disclosed, a significant breakthrough in the field.^8^ All of the aforementioned inhibitors selectively engage “inactive” KRAS and resistance mechanisms have already started to emerge, further driving tumorigenesis.^9–14^ KRAS^G12C^ inhibitor resistance arises from both genomic and non-genetic alterations, with pathways such as Hippo, WNT/β-catenin, Hedgehog, and Notch all having been implicated in cases of resistance.^10^ To mitigate some of these risks, BridgeBio developed BBO-8520, a dual inhibitor of KRAS^G12C^(on) and (off) forms.^15^ BBO-8520 binds in the Switch-II/Helix3 pocket and exhibits an inhibitory effect in models where (off)-only inhibitors demonstrate little activity. In general, molecules engaging the “active” state of KRAS have the potential to moderate some of these resistance risks but have been more challenging to discover due to the picomolar binding affinity of GTP for KRAS.^16^ To this end, Revolution Medicines have reported Cyclophilin A molecular glues capable of inactivating RAS, including KRAS,^17^ Harvey Rat Sarcoma (HRAS), and Neuroblastoma RAS viral oncogene homologue (NRAS) mutants in the “on” state.^18,19^ RMC-6236 (Figure 1), daraxonrasib, which is currently in Phase III clinical trials, engages and inhibits all RAS alleles in the GTP-bound state, preventing tumor growth.^20^ A recent study also provided evidence to suggest that RMC-6291 targeting KRAS^G12C^(on) alone, or in combination with a KRAS^G12C^(off) drug, could be used to circumvent resistance driven by increased levels of active-state KRAS,^21^ while an additional study from Lito et al. demonstrated that some molecular glues which recruit CYPA to GTP-loaded KRAS can enhance the ability of G12X mutants to hydrolyze GTP.^22^ Nevertheless, clinical resistance mechanisms have begun to emerge, with alterations attenuating the formation of the RAS:daraxonrasib:CYPA tricomplex, either through prevention of daraxonrasib binding or by inducing RAF dimers.^23^

**Figure 1:**
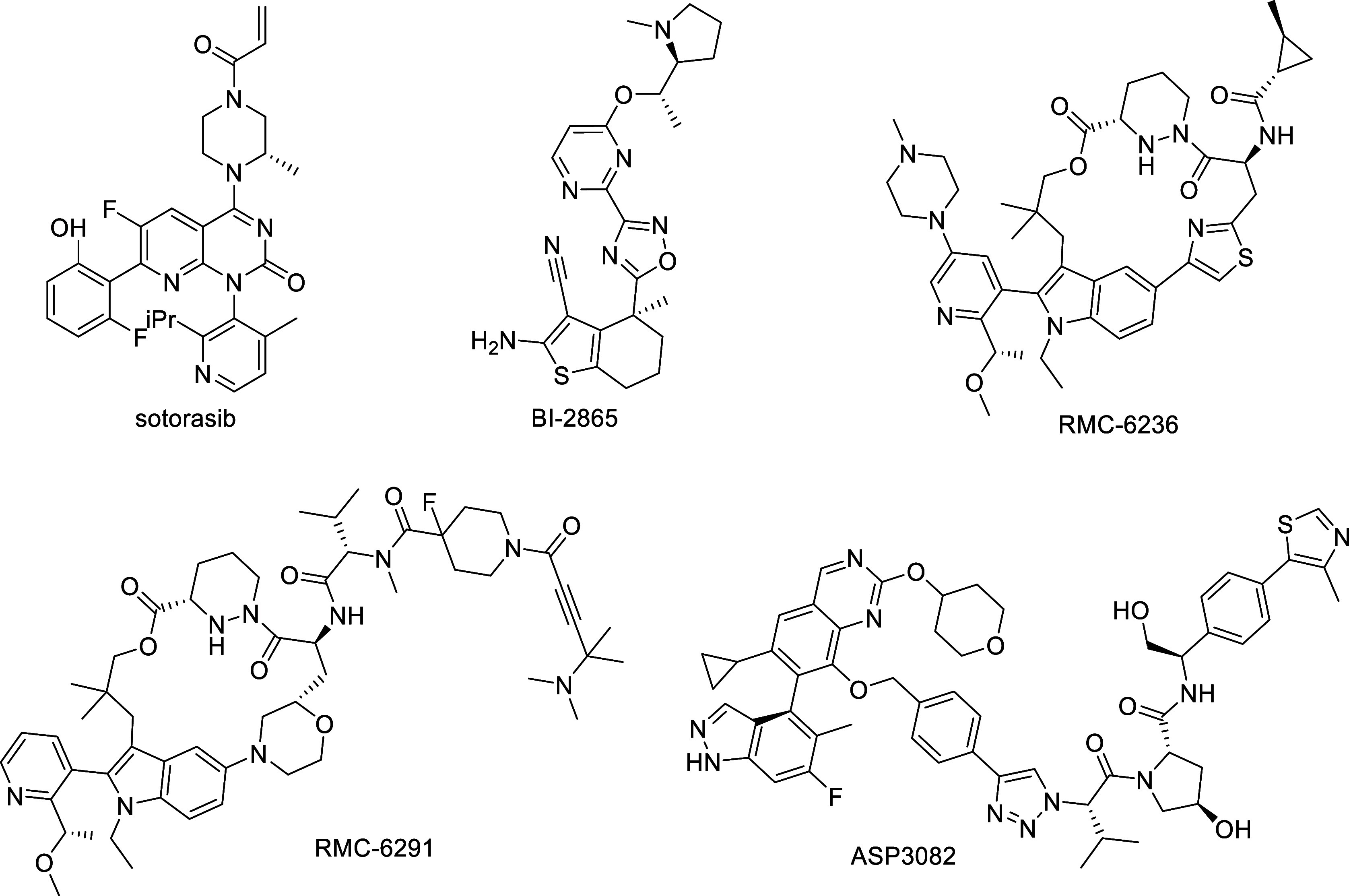
Chemical structures of literature and clinical KRAS inhibitors and degrader.

Proteolysis targeting chimeras (PROTACs) are heterobifunctional modalities, which recruit an E3 ligase to a protein of interest, resulting in subsequent ubiquitination and degradation of the target protein via the ubiquitin-proteasome system. To date, there are >30 PROTAC degrader drugs in clinical trials.^24^ The majority of these are for oncology indications, with one KRAS^G12D^ degrader, the VHL-based PROTAC ASP3082 (Figure 1) from Astellas, currently in Phase I.^25,26^ Since PROTACs require the inclusion of a POI binding ligand, the KRAS degrader landscape has paralleled that of the inhibitors, with the majority of focus on targeting either the G12C or G12D allele.^27–32^ Recently, we reported an in vivo active panKRAS-specific degrader, ACBI3, capable of degrading 13 of the 17 most prevalent KRAS mutants, while sparing H- and NRAS, resulting in more effective pathway modulation across a broad range of KRAS mutant cell lines when compared to inhibition.^33^ However, four alleles were not effectively degraded by ACBI3, namely, G12R, Q61K, Q61L, and Q61R. These mutations affect the ratio of GDP- and GTP-bound KRAS in favor of the GTP-bound state (KRAS­(on)). ACBI3 and related PROTACs, including **1**, instead bind preferentially to GDP-bound KRAS (KRAS­(off)). We hypothesized that a deeper and more durable response could be achieved by degraders engaging the active state of oncogenic KRAS (KRAS­(on)). Herein, we report the use of biophysical data of ternary complexes to determine divergent SAR for PROTACs targeting the “active” state of KRAS and the subsequent discovery of ACBI4, a KRAS-selective degrader capable of depleting multiple KRAS mutants in several cell lines.

## Results

We recently disclosed our efforts toward identifying an in vivo active VHL-based KRAS degrader, with **1** and ACBI3 identified as potent degraders of multiple KRAS­(off) alleles. However, neither was found to effectively degrade G12R, Q61K, Q61L, and Q61R. To this end, we sought to target the KRAS­(on) alleles that are only poorly degraded by ACBI3, with a particular focus on degradation of KRAS^G12R^ as the most prevalent mutant in patients. We hypothesized that degraders for the active state of oncogenic KRAS would elicit a deeper and more durable response against all mutations and enable more effective depletion of the mutants that remained untouched by ACBI3.

It has been extensively shown by us and others, that PROTAC ternary complex affinity often correlates with degradation potency and that improving ternary complex stability and half-life can increase the rate of degradation and improve efficacy.^34–37^ We therefore postulated that faster, more potent degraders could be identified by enhancing the KRAS-GCP:VHL ternary complexes. During and prior to the discovery of ACBI3,^33^ we had designed and synthesized molecules that sought to maximize both binary and ternary complex interactions, guided by our KRAS-GDP:PROTAC:VHL ternary complex structures. Throughout that campaign, we focused on utilizing the KRAS and VHL ligand exit vectors exemplified in ACBI3 and compound **1** while maintaining medium linker lengths. These designs included the replacement of the 5-membered heteroaromatic ring with a cyclopropyl moiety at the exit vector of the VHL binder (compounds **2** and **3**, Figure 2A), which had been shown to be effective in the context of improved VHL ligands, including VH101 (fluoro-cyclopropyl), VH298 (cyano-cyclopropyl), and related analogues.^38–40^ In compound **2**, we also modified the biaryl motif of the VHL ligand to explore different physicochemical property space.^41^ With a desire now to identify compounds capable of forming long-lived KRAS-GCP:VHL ternary complexes, we profiled compounds, such as these, that had been designed to maximize binary and ternary interactions with VHL and KRAS by ternary SPR. We had previously developed^34^ and deployed ternary SPR experiments in prior drug discovery campaigns against this and other targets.^33,42^

**Figure 2:**
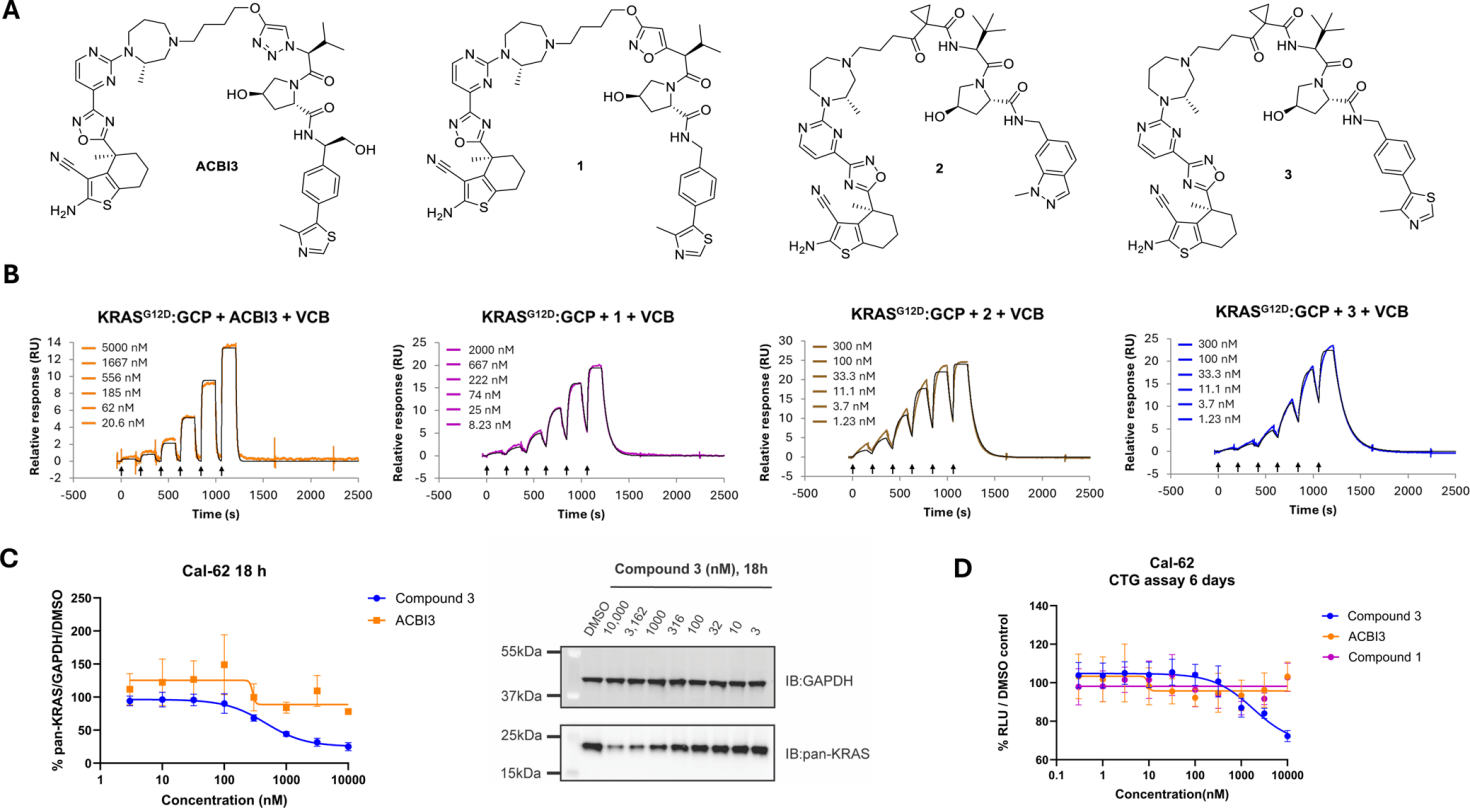
ACBI3-analoguous PROTACs and initial biophysical and cellular profiles against KRAS­(on). (A) Chemical structures of KRAS-VHL PROTACs. (B) Representative ternary SPR sensorgrams for PROTACs **1**–**3** and ACBI3 with KRAS^G12D^-GCP + VCB. (C) Endogenous degradation of KRAS following treatment with PROTAC **3** in Cal-62 cells at 18 h (*N* = 3 biological replicates, SD). (D) Antiproliferative effect in Cal-62 following compound treatment with compounds **1**, **3**, and ACBI3 (*N* = 2 biological replicates, SD).

Upon profiling compounds **2** and **3** against GDP- and guanosine-5′-[(β,γ)-methyleno]­triphosphate (GCP)-loaded KRAS^G12D^ (GCP was used as a nonhydrolyzable mimic for GTP) using SPR, we observed significant improvements in both the binary affinity to KRAS^G12D^-GCP (*K*_d_ = 246 nM and 161 nM, respectively; Tables 1 and S1a) and the ternary affinity to KRAS^G12D^-GCP (*K*_d_ = 39 nM and 38 nM, respectively) in the presence of VCB (VCB = VHL:ElonginC:ElonginB complex) when compared to compound **1** and ACBI3 (binary *K*_d_ = 1466 nM and 719 nM; ternary *K*_d_ = 221 nM and 987 nM, respectively). Both compounds **2** and **3** exhibited positive cooperativity, with α values of 6.7 and 4.2, respectively (α > 1, with α defined as the ratio between binary and ternary *K*_d_). PROTAC **3** was found to have the longest ternary half-life (KRAS^G12D^-GCP + VCB *t*_1/2_ = 93 s) of all four compounds, with a 10-fold improvement over ACBI3 (KRAS^G12D^-GCP + VCB *t*_1/2_ = 9 s). Interestingly, compounds **2** and **3** exhibited similar ternary half-lives for KRAS^G12D^-GDP (KRAS^G12D^-GDP + VCB *t*_1/2_ = 66 and 73 s, respectively; Table S1b) to those for KRAS^G12D^-GCP (*t*_1/2_ = 56 and 93 s; Tables 1 and S1a). This trend strikingly contrasts to that of compound **1** and ACBI3, which instead exhibited much longer half-lives for the GDP-loaded KRAS^G12D^ allele (KRAS^G12D^-GDP + VCB *t*_1/2_ = 225 and 196 s, respectively; Table S1b), compared to those for KRAS^G12D^-GCP + VCB (*t*_1/2_ = 42 and 9 s; Tables 1 and S1a). All four PROTACs were found to have ternary dissociation constants <10 nM for KRAS^G12D^-GDP + VCB. Work from Alexander et al. investigating the biophysical and structural characterization of switch-II pocket binders revealed allele-specific as well as nucleotide-dependent binding constraints resulting from the switch-II pocket being less accessible in the GppNHp bound state.^43^ Binary SPR data for BI-2865, ACBI3, and PROTAC **1** (Table S2) suggested no significant differences in affinity between alleles for GDP-loaded KRAS. However, additional SPR experiments were conducted with KRAS^G12R^-GCP to investigate whether allele-specific differences were observed for the active state. Affinity and half-life values were found to be largely similar between the two alleles for compounds **1**–**3** (Table 1 and Figures S5 and S6). Interestingly, the largest affinity difference was observed for ACBI3 (KRAS^G12D^-GCP + VCB *K*_d_ = 987 nM, KRAS^G12R^-GCP + VCB *K*_d_ = 366 nM) although half-lives were comparable (KRAS^G12D^-GCP *t*_1/2_ = 9 s, KRAS^G12R^-GCP *t*_1/2_ = 6 s).

**Table 1:** Binary and Ternary SPR Dissociation Constants, Ternary Half-Lives, and Cooperativity Values for PROTACs **1**–**3** and ACBI3 with KRAS^G12D^-GCP ± VCB and KRAS^G12R^-GCP ± VCB^a^

	ACBI3	**1**	**2**	**3**
KRAS^G12D^-GCP *K*_d_ (nM)	719 ± 147	1466 ± 193	246 ± 50	161 ± 22
KRAS^G12D^-GCP + VCB *K*_d_ (nM)	987 ± 241	221 ± 12	39 ± 9	38 ± 2
cooperativity (α)	0.8 ± 0.1	6.6 ± 0.6	6.7 ± 2.2	4.2 ± 0.5
KRAS^G12D^-GCP + VCB *t*_1/2_ (s)	9 ± 2	42 ± 1	56 ± 4	93 ± 6
				
KRAS^G12R^-GCP *K*_d_ (nM)	252 ± 13	1397 ± 186	167 ± 23	169 ± 10
KRAS^G12R^-GCP + VCB *K*_d_ (nM)	366 ± 26	201 ± 31	21 ± 3	23 ± 1
cooperativity (α)	0.7 ± 0.03	7.4 ± 2.6	8.3 ± 1.8	7.3 ± 0.6
KRAS^G12R^-GCP + VCB *t*_1/2_ (s)	6 ± 1	34 ± 3	97 ± 7	52 ± 3

a Values are derived from single cycle experiment kinetic fitting (*N* = 3 to 5 independent experiments, ±SD).

Having identified compound **3** as a cooperative PROTAC with the lowest ternary *K*_d_ and the longest ternary half-life with GCP-loaded KRAS by SPR, we sought to explore its degradation profile in cell lines exhibiting different KRAS mutations to ascertain whether greater ternary complex stability for GCP-loaded KRAS would indeed improve the degradation efficacy of the KRAS^G12R^ mutant (Figure 2C). PROTAC **3** dose dependently degraded KRAS in the Cal-62 KRAS^G12R^ cell line with a DC_50_ = 462 nM at 18 h and maximal degradation of 75%, while ACBI3 and **1** had no effect. Similarly, KRAS degradation was also observed in the KP-2 cell line upon treatment with compound **3** (DC_50_ = 162 nM, *D*_max_ = 60% at 18 h; Table S3a). Meanwhile, in GP5d cells presenting a G12D mutation, all PROTACs performed to a similar level (Table S3a). Antiproliferative effects were also found to correlate with degradation for the Cal-62 cell line, with PROTAC **3** exhibiting a dose-dependent antiproliferative effect with an IC_50_ of 1.5 μM.

We next aimed to solve the structure of the PROTAC **3** ternary complex to try and rationalize a potential structural basis for the improved affinity toward GCP-loaded KRAS. Leveraging established cocrystallization protocols,^33,44^ we crystallized ternary complexes of VCB-PROTAC-KRAS with KRAS^G12V^-GDP and KRAS^G12R^-GCP as representations of KRAS­(off) and -(on) (Figure 3). We obtained suitable cocrystal ternary complex structures of **1** with KRAS^G12R^(GCP)-VCB (Figure 3A) and **3** with both KRAS^G12V^(GDP)-VCB (Figure 3B) and KRAS^G12R^(GCP)-VCB (Figure 3C,E) at resolutions of 2.83, 2.63, and 2.89 Å, respectively. We previously disclosed the ternary complex structure of PROTAC **1** with KRAS^G12V^(GDP)-VCB.^33^ Analysis of the ternary complex structures of **1** and **3** with both KRAS^G12R^(GCP) and KRAS^G12V^(GDP) showed that the same relative orientation as previously solved cocrystal structures was adopted,^33^ regardless of the mutation or nucleotide (Figure 3D). The main observed difference was the loss of interaction between **3** and Tyr112 of VHL, relative to that of PROTAC **1**. Chemically, **3** differs from **1** only at the point of attachment to the VHL binder, where it presents a cyclopropyl 1,3-dicarbonyl in place of the isoxazole of **1**. This modification alters the interaction potential between the PROTAC and Tyr112 of VHL, which may partially explain the shorter ternary half-life observed for **3** in KRAS^G12V^-GDP + VCB (**3** = 70 s and **1** = 228 s). However, there were no significant differences observed at the KRAS–VHL binding interface to explain the observed difference in the ternary kinetic stability between the two compounds. As expected, differences in the Switch II loop are observed between the G12V­(GDP) and G12R­(GCP) variants for each compound.^45^ Interestingly, for **3**, very small differences are also observed in the base-binding loop regions between the two differentially loaded mutants. Nonetheless, without clear structural rationale for the improvement in affinity toward KRAS­(on), we maintained focus on optimization utilizing biophysical and in vitro assays.

**Figure 3:**
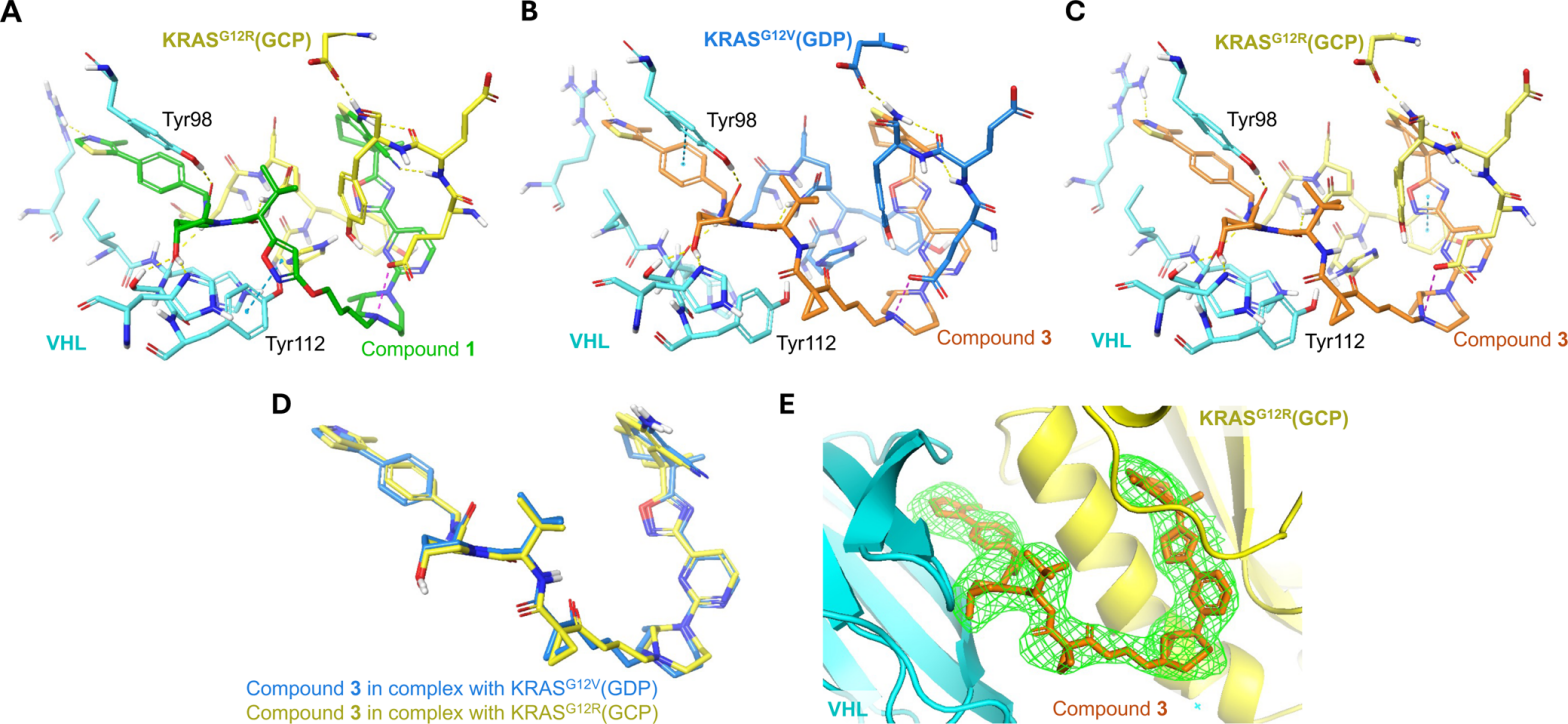
Cocrystal structures of KRAS:PROTAC:VHL ternary complexes. (A) Binding interface of **1** [green] -induced ternary complex between KRAS^G12R^(GCP) [yellow] and VHL [cyan], PDB: 9RKE; (B) binding interface of **3** [orange] -induced ternary complex between KRAS^G12V^(GDP) [blue] and VHL [cyan], PDB: 9RK8; (C) binding interface of **3** [orange] -induced ternary complex between KRAS^G12R^(GCP) [yellow] and VHL [cyan], PDB: 9RKJ; (D) overlay of **3** bound to KRAS^G12V^(GDP) [blue] and **3** bound to KRAS^G12R^(GCP) [yellow]; (E) polder Fo–Fc map [green] is shown contoured at 3σ around the modeled compound **3** [orange] bound with KRAS^G12R^(GCP) [yellow] and VHL [cyan].

Having observed promising levels of endogenous degradation upon treatment with PROTAC **3** in Cal-62 (KRAS^G12R^) cells (Figure 2C), we sought to generate a HiBiT-KRAS^G12R^ cell line to enable focused screening of PROTACs with varying linkers to identify more potent G12R degraders. Genetic depletion using siRNA and antiproliferative experiments were performed in three cell lines exhibiting G12R mutations (Cal-62, PSN-1 and NCI-H157; Figure S7) to understand the effect of KRAS knockdown in each. Genetic depletion had the fastest and most profound effect in the Cal-62 cell line. Additionally, treatment with **3** induced the greatest antiproliferative effect in Cal-62, while the other cell lines exhibited a partial response. KRAS^G12R^ is homozygously expressed in Cal-62 cells, which also contain the smallest copy number (Cal-62 absCN *N* = 3; PSN-1 absCN *N* = 9.1; NCI-H157 absCN *N* = 4.9) of the three tested cell lines. Typically, there is a higher likelihood of making a homozygous knock-in where there is a lower copy number. This, combined with the depletion and antiproliferative data, made Cal-62 the best candidate for genetic insertion of the HiBiT tag, so we next generated a HiBiT-KRAS^G12R^ Cal-62 knock-in cell line using the CRISPR Cas9 technology. We previously reported the use of a HiBiT-KRAS^G12D^ GP5d cell line for profiling.^33^ A series of compounds were analyzed in both the GP5d-G12D and Cal-62-G12R HiBiT cell lines at 4- and 24 h time points, which led to the identification of PROTAC **4** (a mixture of four diastereoisomers; Figure 4A) as a potent KRAS^G12R^ degrader. Compound **4** was originally designed as part of the campaign to identify KRAS­(off) degraders, to maintain the π-stacking interaction with Tyr112 on VHL, which had been observed for **1** and ACBI3, while rigidifying the linker. In the HiBiT cell lines (Figure 4D), PROTAC **4** was found to be significantly more efficacious than **3**, exhibiting a ∼9-fold improvement in DC_50_ at 24 h for KRAS^G12D^ (**4**: *D*_max_ = 94%, DC_50_ = 4 nM at 24 h; **3**: *D*_max_ = 92%, DC_50_ = 37 nM at 24 h) and both improved DC_50_ and *D*_max_ against KRAS^G12R^ (**4**: *D*_max_ = 87%, DC_50_ = 183 nM at 24 h; **3**: *D*_max_ = 69%, DC_50_ = 483 nM at 24 h). The starkest contrast was observed for KRAS^G12R^ degradation at the 4 h time point where **3** shows negligible degradation (*D*_max_ = 21%) in HiBiT-KRAS^G12R^ Cal-62 cells compared to **4** (*D*_max_ = 76%, DC_50_ = 163 nM at 4 h). This data was corroborated using a kinetic degradation assay which also showed **4** to degrade HiBiT-KRAS^G12R^ at a significantly faster rate than **3** (Figure 4B). To investigate whether degradation in the HiBiT cell lines translated to robust endogenous degradation of both KRAS^G12R^ and KRAS^G12D^, we treated cells exhibiting these mutations with compounds **3** and **4** and quantified endogenous protein levels by Western blot (Figure 4E). Both PROTACs performed similarly in Cal-62, KP-2, and GP5d cells; however, degradation of KRAS^G12D^ in GP5d cells also occurs at a much faster rate, leading us to report degradation at the earlier 4 h time point. Compound **3** also showed a very strong hook effect in the GP5d cell line. This effect is seen to a lesser extent in the comparatively similar KRAS^G12D^-HiBiT cell line. PROTAC **4** was also found to effect improved maximal degradation (*D*_max_ = 70%) of KRAS^G12R^ in the KP-2 cell line. The increased rate of degradation exhibited by compound **4** also led to improved antiproliferative effect in Cal-62 cells (**4**: IC_50_ = 488 nM) (Figure 4C).

**Figure 4:**
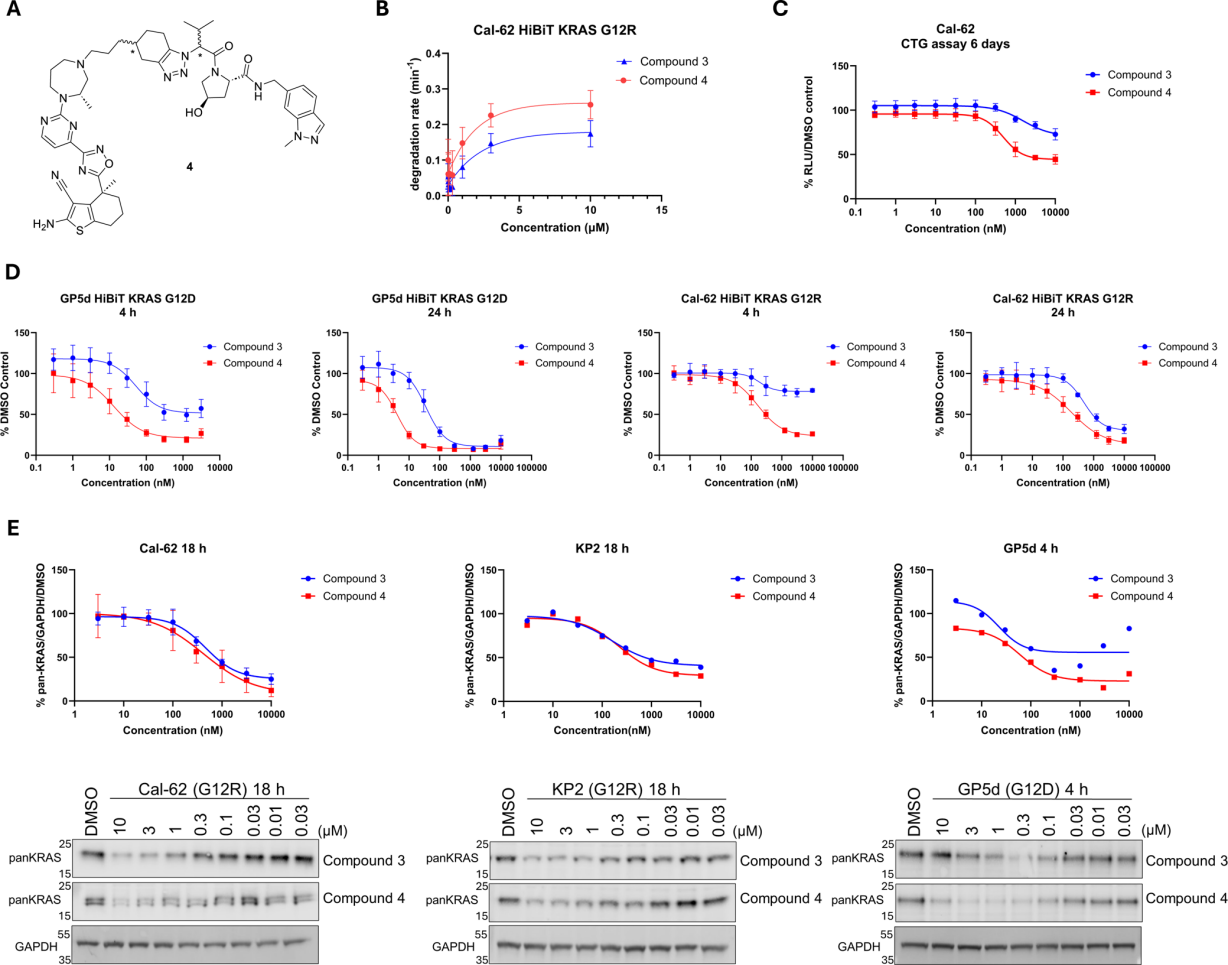
Compound **4** is a potent, fast degrader of KRAS^G12R^. (A) Chemical structure of PROTAC **4**. (B) Degradation rates of HiBiT-KRAS^G12R^ for compounds **3** and **4** (*N* = 5 biological replicates, SD). (C) Comparison of antiproliferative effects of **3** and **4** in Cal-62 cells (*N* = 2 biological replicates, SD). (D) Degradation profiles of PROTACs **3** and **4** in HiBiT-KRAS^G12R^ Cal-62 and HiBiT-KRAS^G12D^ GP5d at 4 and 24 h time points (HiBiT GP5d 4 h and 24 h, HiBiT Cal-62 24 h: *N* = 3 biological replicates with duplicate technical repeats, SD. HiBiT Cal-62 4 h: *N* = 2 biological replicates with duplicate technical repeats, SD). (E) Comparison of endogenous degradation effected by compounds **3** and **4** in Cal-62 (KRAS^G12R^), Calu-6 (KRAS^Q61K^), and SW948 (KRAS^Q61L^) measured by Western blot analysis at 18 h and GP5d (KRAS^G12D^) at 4 h (*N* = 3 biological replicates, SD).

Separation of **4** into all four of its diastereoisomers was carried out by supercritical fluid chromatography (SFC) to give PROTACs **5a**, **5b**, **5c**, and ACBI4, which were profiled in both HiBiT-KRAS^G12D^ and HiBiT-KRAS^G12R^ cell lines. Two isomers were revealed to be more efficient degraders, with ACBI4 exhibiting the most promising profile in HiBiT-KRAS^G12R^ Cal-62 at the 4 h time point (Figure 5A). SPR data also confirmed ACBI4 to induce the most stable ternary complex of the two more active diastereoisomers (Figure 5D and Table 2), for both GCP- and GDP-loaded KRAS (KRAS^G12D^-GCP + VCB: *t*_1/2_ = 3,283 s, *K*_d_ = 0.9 nM; KRAS^G12R^-GCP + VCB: *t*_1/2_ = 1,863 s, *K*_d_ = 1.6 nM; KRAS^G12D^-GDP + VCB: *t*_1/2_ = 11,699 s, *K*_d_ = 0.08 nM), with >35-fold improvement over the initial hit **3** for KRAS­(on)^G12D^ and ∼20-fold for KRAS­(on)^G12R^. The cooperativity value for the formation of both KRAS^G12D^-GCP + VCB and KRAS^G12R^-GCP + VCB ternary complex also substantially improved, with ACBI4 featuring the largest α value (α = 143 and α = 106, respectively) compared to diastereomer **5c** and compound **3** (KRAS^G12D^-GCP α = 60 and α = 4.2 and KRAS^G12R^-GCP α = 89 and α = 7.3, respectively) (Table 2). Potent degradation of endogenous KRAS^G12R^ was achieved by ACBI4 in the Cal-62 cell line (*D*_max_ = 93%, DC_50_ = 162 nM at 18 h), translating to an antiproliferative effect (Figure 5C). No antiproliferative effect was observed in the KRAS-independent A375 cell-line (Figure S12), indicating an on-target effect in Cal-62 cells. Competition experiments with a 1 h pre-treatment of the VHL ligand VH298, proteasomal (MG132) or neddylation (MLN4924) inhibitors confirmed that ACBI4 degrades its target via a PROTAC mode of action (Figure 5E).

**Figure 5:**
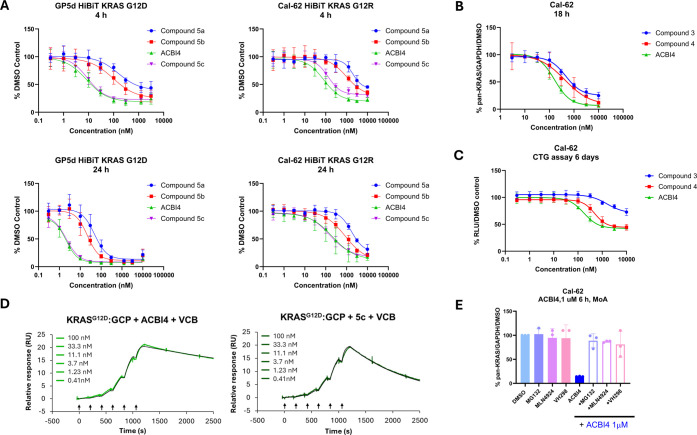
ACBI4 forms the most cooperative and stable ternary complexes and acts as the most potent degrader of KRAS^G12R^ amongst the four diastereoisomers of compound **4**. (A) HiBiT-KRAS^G12R^ and KRAS^G12D^ degradation induced by compounds **5a**, **5b**, **5c**, and ACBI4 at 4 and 24 h time points (*N* = 3 biological replicates, SD). (B) Comparison of endogenous KRAS degradation in Cal-62 cells by **3**, **4**, and ACBI3 (*N* = 3 biological replicates, SD). (C) Comparison of antiproliferation in Cal-62 cells by **3**, **4**, and ACBI3 (*N* = 3 biological replicates, SD). (D) Representative ternary SPR sensorgrams for PROTAC **5c** and ACBI4 with KRAS^G12D^-GCP + VCB. (E) Western blot analysis of KRAS levels following treatment with DMSO or ACBI4 ± VH298, MG132, and MLN4924 (*N* = 3 biological replicates, SD).

**Table 2:** Binary and Ternary SPR Dissociation Constants, Ternary Half-Lives, and Cooperativity for PROTACs **5c** and ACBI4 with KRAS^G12D^-GCP + VCB and KRAS^G12R^-GCP ± VCB (*N* = 3 or 4 Independent Experiments, ±SD)

	ACBI4	**5c**
KRAS^G12D^-GCP *K*_d_ (nM)	125 ± 16	194 ± 40
KRAS^G12D^-GCP + VCB *K*_d_ (nM)	0.9 ± 0.2	3.4 ± 0.8
KRAS^G12D^-GCP + VCB *t*_1/2_ (s)	3283 ± 155	837 ± 54
cooperativity (α)	143 ± 25	60 ± 14
		
KRAS^G12R^-GCP *K*_d_ (nM)	141 ± 32	179 ± 24
KRAS^G12R^-GCP + VCB *K*_d_ (nM)	1.6 ± 0.8	2.1 ± 0.6
KRAS^G12R^-GCP + VCB *t*_1/2_ (s)	1863 ± 149	710 ± 33
cooperativity (α)	106 ± 44.4	89 ± 25

We consequently aimed to determine the stereochemical configuration of ACBI4 and rationalize what was driving cooperativity by solving its ternary complex structure. We crystallized ternary complexes of VCB-PROTAC-KRAS^G12D^-GDP and -KRAS^G12R^-GCP with ACBI4 and solved the cocrystal structures of both at resolutions of 2.19 Å and 2.85 Å, respectively. We observed that the overall binding mode remained the same as previous PROTAC analogues for both KRAS^G12D^-GDP and KRAS^G12R^-GCP bound structures (Figure 6B). At these resolutions, the stereoconfiguration of the isopropyl group could not be unequivocally assigned from the map density (Figures 6C and S13). Based on extensive knowledge of the stereochemical preference of the alkylic side chain at that same α-carbon in high-affinity VHL ligands^39,40^ and our ternary structures of compounds **1**, **3**, and ACBI3,^33^ the α-carbonyl stereocenter bearing the isopropyl group has been assigned as the (*S*)-configuration in our refined structure.

**Figure 6:**
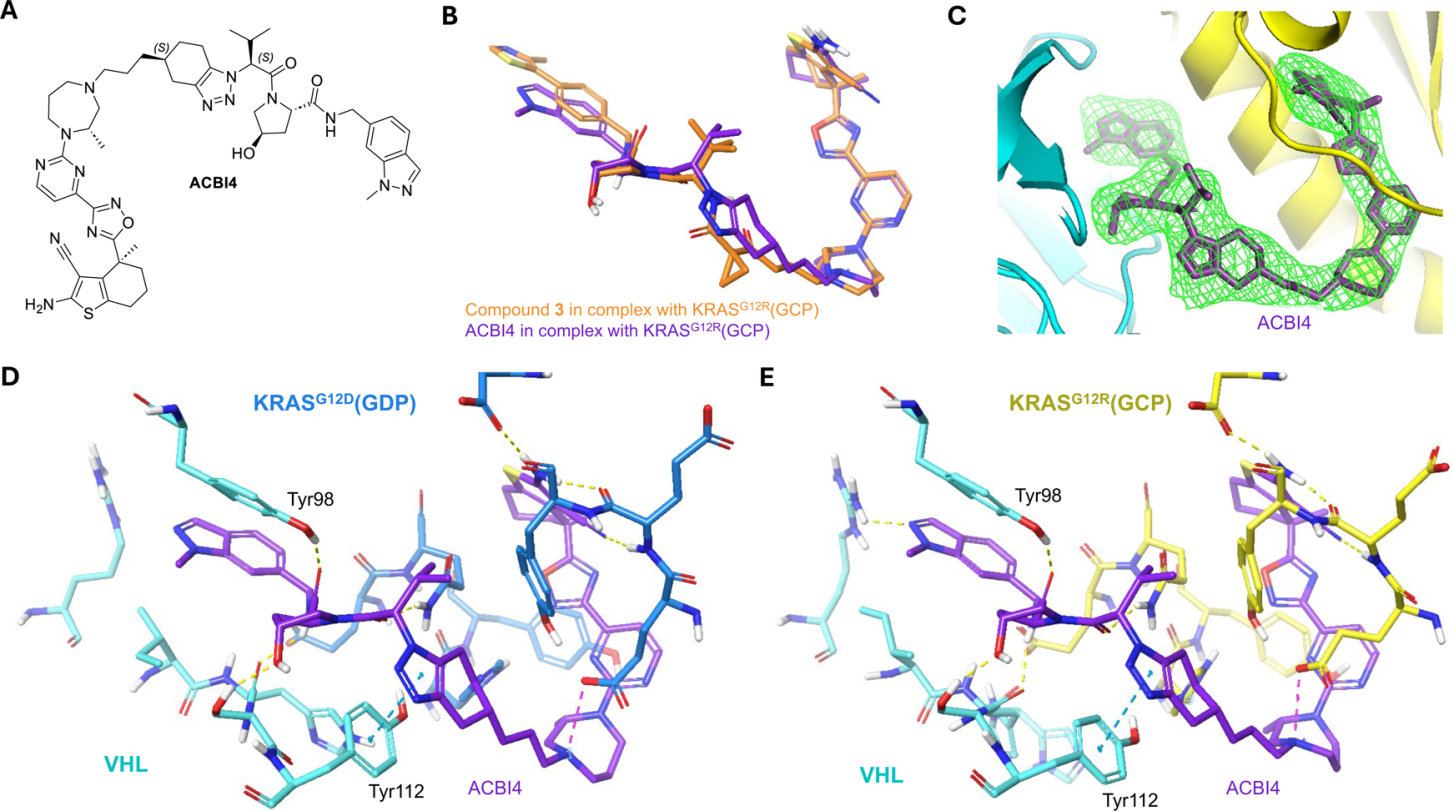
X-ray cocrystal structures of ACBI4 bound in ternary complexes with VHL and KRAS^G12R^ or KRAS^G12D^ reveal PROTAC binding mode. (A) Chemical structure of ACBI4 with postulated stereochemical assignment. (B) Overlay of compound **3** [orange] and ACBI4 [purple] bound to KRAS^G12R^(GCP). (C) Polder Fo–Fc map [green] is shown contoured at 3 s around the modeled compound ACBI4 [purple] bound with KRAS^G12R^(GCP) [yellow] and VCB [cyan]. (D) Binding interface of ACBI4 [purple] induced ternary complex between KRAS^G12D^(GDP) [blue] and VCB [cyan], PDB: 9RKC. (E) Binding interface of ACBI4 [purple] induced ternary complex between KRAS^G12R^(GCP) [yellow] and VCB [cyan], PDB:9RKN.

To ascertain whether our newly identified degrader ACBI4 exhibited selectivity for KRAS, Cal-62 (KRAS^G12R^) cells were treated with either ACBI4 (1 μM, 6 h) or a negative control containing a cis-hydroxyproline moiety in the VHL binding portion of the molecule. As expected, KRAS was found to be significantly downregulated by ACBI4 in comparison to the negative control compound (Figure 7A). Previous whole cell proteomics analysis of GP2d cells (KRAS^G12D^) treated with PROTAC **1** had also shown significant KRAS degradation.^33^ Additionally, levels of neuroblastoma RAS viral oncogene homologue (NRAS), Fos-related antigen 1 (FOSL1) and G0/G1 switch protein 2 (GOS2) were found to be significantly reduced. NRAS is a closely related GTPase, while FOSL1 is abnormally expressed in many tumors and known to be linked to the MAPK pathway.^46^ Cyclin-dependent kinase inhibitor 1C (CDKN1C) and Ras homologue family member B (RHOB) were both found to be upregulated. CDKN1C is involved in cell-cycle regulation, while RHOB upregulation has previously been observed upon MAPK pathway inhibition.^47,48^ Interestingly, Western blot analysis of Cal-62 cells treated with ACBI4 showed the degradation of NRAS only at very high concentrations (Figure S9). In addition, downregulation of Harvey rat sarcoma viral oncogene homologue (HRAS) was observed at higher concentrations, reaching maximal degradation at 3 μM (DC_50_ = 637 nM, *D*_max_ = 72%; Figure S9), representing a ca. 2-fold window over KRAS degradation (DC_50_ = 382 nM, *D*_max_ = 88%; Table S3a). Selective targeting of KRAS while sparing HRAS and NRAS has previously been thought important to maintain a wide therapeutic window.^8^ However, thresholds for RAS isoform specificity required for safety are not established, and RMC-6236, a panRAS inhibitor, has advanced successfully into Phase III clinical trials.^49^

**Figure 7:**
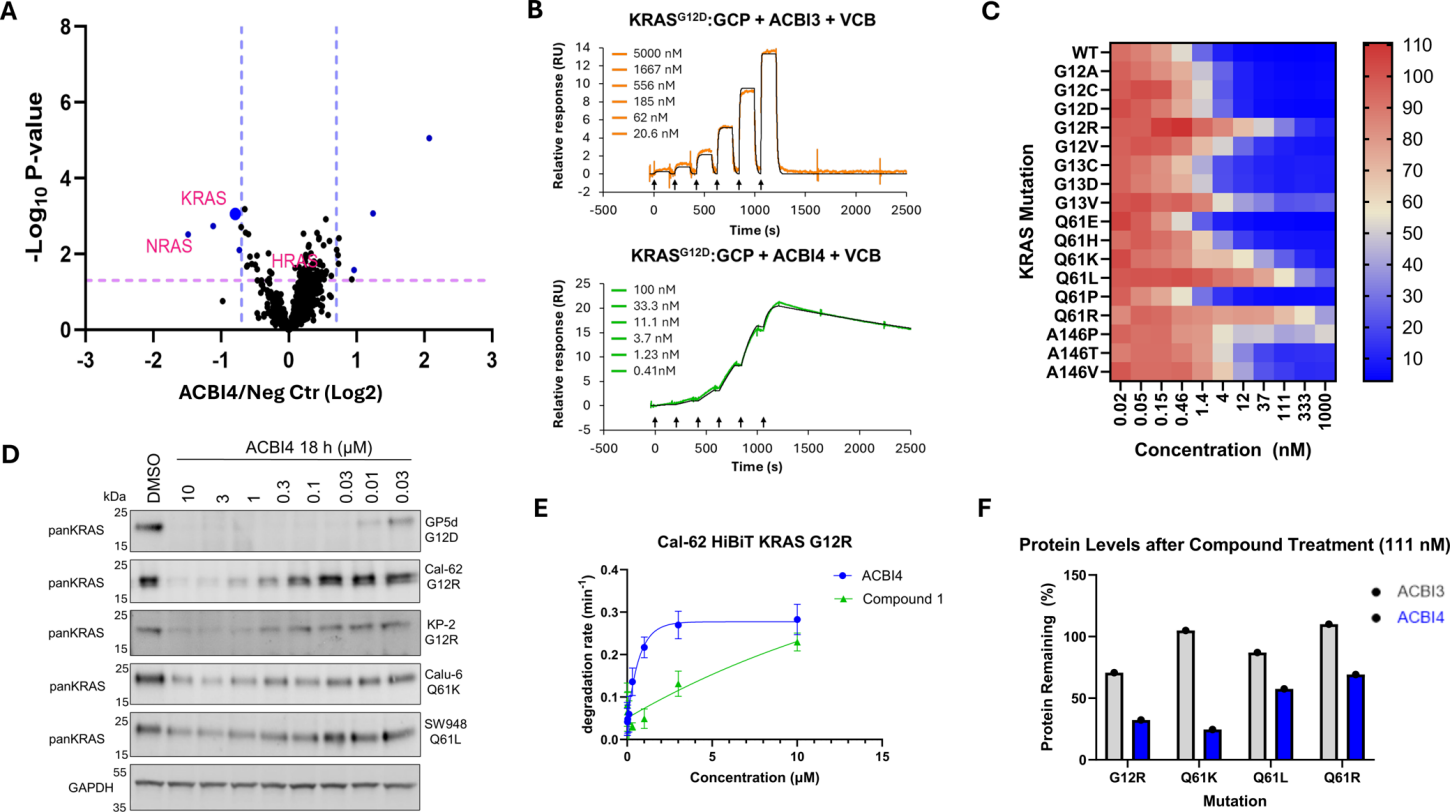
ACBI4 qualifies as a potent, fast, and proteome-wide selective degrader of KRAS^G12R^ and other KRAS­(on) alleles. (A) Whole cell proteomics MS analysis of Cal-62 cells treated for 6 h with 1 μM ACBI4 compared to inactive negative control. KRAS, HRAS, and NRAS are highlighted in pink. (B) Representative SPR sensorgrams of KRAS^G12D^-GCP + VCB ternary complex formation with ACBI3 and ACBI4. (C) Degradation of retrovirally transduced HiBiT-tagged KRAS mutants by ACBI4 in GP5d cells at 18 h (*N* = 3 biological replicates, SD). (D) Western blot analysis of KRAS^mutant^ levels in different cell lines following treatment with ACBI4. (E) HiBiT-KRAS^G12R^ degradation rate for compound **1** and ACBI4 (*N* = 5 biological replicates, SD). (F) Protein levels remaining of retrovirally transduced HiBiT-tagged KRAS mutants G12R, Q61K, Q61L, and Q61R following treatment with ACBI3 or ACBI4 (*N* = 3 biological replicates, SD).

Having established ACBI4 to be a selective degrader, we sought to investigate its effects on KRAS mutants beyond G12R and G12D and whether it could address all KRAS­(on) mutations. To this end, ACBI4 was tested against a panel of retrovirally transduced HiBiT-tagged KRAS mutants and found to induce the degradation of all KRAS alleles (Figure 7C). Except for the A146P mutant, ACBI4 showed improved levels of degradation when compared to ACBI3, with the most profound difference observed for the KRAS­(on) G12R, Q61K, Q61L, and Q61R mutations (Figure 7F). Evaluation of PROTAC-induced degradation of endogenous KRAS­(on) mutants KRAS^Q61L^ and KRAS^Q61K^ was also carried out by Western blot in Calu-6 and SW948 cells, in addition to KRAS^G12R^ degradation in the KP-2 cell line (Figure 7D). In line with the exogenous HiBiT-data, ACBI4 was found to degrade endogenous KRAS^G12R^, KRAS^Q61L^, and KRAS^Q61K^ in a concentration-dependent manner, with higher concentrations required to effect degradation in the SW948­(KRAS^Q61L^) cell line. The GP5d cell line, expressing KRAS^G12D^, was included for comparison of the degradative effect on endogenous KRAS­(off) alleles. Kinetic experiments revealed that ACBI4 degrades HiBiT-KRAS^G12R^ at a rate significantly faster than those of previous analogues (Figure 7E), as expected from the improvement in KRAS^G12D^-GCP ternary complex stability (Figure 7B). These data support that ACBI4 is a selective KRAS targeted protein degrader capable of degrading all prevalent oncogenic KRAS mutants and inducing antiproliferative effects in cell lines dependent on the most challenging KRAS alleles. Notably, this has been achieved using a KRAS inhibitor that alone was previously found to have 60–140 times lower affinity for KRAS variants loaded with GCP.^8^

## Conclusions

Targeting KRAS with small molecules is highly challenging due to the wide range of mutations that can cause oncogenic activation. We recently reported PROTACs capable of potently and selectively degrading most KRAS mutants,^33^ and panRAS molecules that form complexes with cyclophilin have been disclosed and are currently under clinical investigation.^20,49^ Despite these important advances, there remains a need for small molecules capable of specifically addressing the most intractable KRAS alleles, which are hydrolysis-incompetent and display greater bias toward the GTP-active state. Such tumor-driving alleles are prevalent in several cancers, with KRAS^G12R^ among the five most observed mutant isoforms. Herein, we describe the use of biophysical and cellular in vitro assays supported by high-resolution cocrystal structures of ternary complexes, leading to the discovery of ACBI4, a panKRAS degrader capable of targeting all KRAS variants. ACBI4 effectively engages the GTP-activated state of KRAS, to drive rapid and profound degradation of KRAS­(on) mutants, leading to antiproliferative effect in a cell line driven by KRAS^G12R^. This was achieved by these PROTACs despite the relatively weak binding affinity for KRAS­(on) of the KRAS ligand they are built of (KRAS^G12D^-GCP binary *K*_d_ ranging from 1.47 μM to 125 nM across the compound series). The enhanced target engagement capacity arises because of the high level of ternary complex cooperativity attained by the compounds through the formation of highly stable interactions, culminating in ACBI4 as the degrader forming the most cooperative and most long-lived ternary complex (KRAS^G12D^-GCP:ACBI4:VHL α ∼ 140 and *t*_1/2_ ∼ 55 min, respectively). These findings therefore highlight how biophysics-guided optimization of stable and cooperative PROTAC ternary complexes can enable degradation without the requirement for high-affinity binders.^50,51^

The pursuit of a single agent that could specifically and simultaneously address all oncogenic KRAS alleles represents a major drug discovery endeavor. The discovery of ACBI4 represents a significant stride forward toward this goal, addressing long-considered “undruggable” variants of KRAS in a way not possible to date and thus substantially expanding scope beyond the coverage attained by current KRAS degraders ACBI3 ^33^ or ASP3082.^26^ This study provides a new molecule that we elect as meeting key criteria of degradation potency, speed, and selectivity to qualify it as a degrader chemical probe for biomedical research^52,53^ and intend to make it available for unrestricted, cost-free access via the opnMe platform.^54^ We envisage future utilization will aid greater understanding of the therapeutic potential of degrading KRAS­(on) and will pave the way for the development of future agents for the treatment of KRAS-driven malignancies.

## Supplementary Material



## Data Availability

Atomic coordinates and structure factors for X-ray crystallographic structures have been deposited in the Protein Data Bank with accession codes 9RK8, 9RKC, 9RKE, 9RKJ and 9RKN. All raw data and search results for proteomics studies have been deposited to the ProteomeXchange Consortium (PRIDE) with the data set identifier PXD065163.

## Notes

Experimental procedures and supplementary tables and figures (PDF)

## ∥

Computational Chemistry, Nxera Pharma U.K., Steinmetz Building, Granta Park, Cambridge CB21 6DG, U.K

## ⊥

Respiratory & Immunology (R&I), Research and Early Development, BioPharmaceuticals R&D, AstraZeneca, Pepparedsleden 1, 43183 Mölndal, Sweden.

## #

Solgate GmbH, Klosterneuburg 3400, Austria.

## ¶

The Institute of Cancer Research, 123 Old Brompton Road, London, SW7 3RP, U.K.

## ∇

Research Office, University of Edinburgh, Edinburgh EH1 1HT, U.K.

## ○

Australian Centre for Targeted Therapeutics, WEHI, 4 Research Avenue, Bundoora, Victoria 3086, Australia.

## ⧫

Mironid Ltd., Hamnett Wing, Arbuthnott Building, 161 Cathedral Street, Glasgow G4 0RE, U.K.

## §§

Kalbe Farma TBK, Jln. Let. Jend Suprapto Kav 4, Jakarta 10510, Indonesia.

## ⬡

CatSci Ltd, Biocity Nottingham, Pennyfoot Street, NG1 1GF Nottingham, U.K.
