# Supplementary material for: Identification of a Highly Cooperative PROTAC Degrader Targeting GTP-Loaded KRAS(On) Alleles
Source: J Am Chem Soc. 2025 Oct 30;147(45):41367–78. doi: 10.1021/jacs.5c10354 (PMC12616682; doi:10.1021/jacs.5c10354)
Supplement: Supplementary file 1 [file ja5c10354_si_001.pdf]

Identification of a Highly Cooperative PROTAC Degrader Targeting GTP-loaded KRAS(on) Alleles

Supplementary Information

Vesna Vetma[1,2,†], Ilaria Puoti[1,†], Natalia K. Karolak[1,†], Sohini Chakraborti[1,2], Emelyne Diers[2], Enrico Girardi[3], Shakil Khan[1,2], Giorgia Kidd[1,2], Katrin G. Kropatsch[3], Ross Mclennan[1,2], Suzanne O'Connor[1,2], Matthias Samwer[3], Nicole Trainor[2], Claire Whitworth[2], Andre Wijaya[1,2], Jeff Y. F. Wong[1,2], David Zollman[1,2], William Farnaby[1,2], Johannes Popow[3], Alessio Ciulli[1,2,*], Peter Ettmayer[3,*], Kirsten McAulay[1,*]

[1]Centre for Targeted Protein Degradation, School of Life Sciences, 1 James Lindsay Place, University of Dundee, DD1 5JJ, Dundee, Scotland, U.K.

[2] Division of Biological Chemistry and Drug Discovery, School of Life Sciences, James Black Centre, University of Dundee; DD1 5EH Dundee, Scotland, U.K.

[3] Boehringer Ingelheim RCV GmbH & Co KG, 1221 Vienna, Austria

[†] These authors contributed equally to this work

[*]Corresponding authors Emails: a.ciulli@dundee.ac.uk; peter.ettmayer@boehringer-ingelheim.com; KMcaulay001@dundee.ac.uk

Contents

Methods and Materials 4
Protein Production 4
Nucleotide exchange 4
SPR 5
Protein Crystallography 5
Cell Culture 6
Cell Line Generation 7
Degradation Assays 7
Degradation via Detection of Split Nanoluciferase (HiBiT) 7
Kinetic Degradation 8
Retroviral Transduction of HiBiT-tagged KRAS Mutants 8
Western Blot 8
CRISPR Depletion Experiments 9
Anti-Proliferation 9
Proteomics 9
Chemical Synthesis 11
General Information 11
Analytical MS Methods and instrumentation 11
Experimental Procedures 12
NMR Data 21
Supplementary Data and Figures 28
Table S1a: SPR KRAS^{G12D}-GCP Characterisation Data Summary 28
Table S1b: SPR KRAS^{G12D}-GDP Characterisation Data Summary 30
Table S1c: SPR KRAS^{G12R}-GCP Characterisation Data Summary 32
Table S1d: SPR VCB Ternary Characterisation Data Summary 34
Figure S1: SPR characterization of binary and ternary complexes for immobilized KRAS^{G12D}-GCP with VCB 36
Figure S2: SPR characterization of binary and ternary complexes for immobilized KRAS^{G12D}-GDP with VCB 38
Figure S3: SPR characterization of binary and ternary complexes for immobilized KRAS^{G12R}-GCP with VCB 40
Figure S4: SPR characterization of ternary complexes for immobilized VCB with KRAS^{G12D}-GCP or KRAS^{G12D}-GDP 43
Table S2. Comparison of KRAS^{G12X}-GDP Binary SPR Affinity for BI-2865, ACBI3 and PROTAC **1** 44

Figure S5. Comparison of SPR Afffinities for PROTACs with $KRAS^{G12D}$-GCP±VCB and $KRAS^{G12R}$-GCP±VCB 45

Figure S6. Comparison of Half-Lives for PROTACs with $KRAS^{G12D}$-GCP±VCB and $KRAS^{G12R}$-GCP±VCB 45

Table S3a. Data Summary *in vitro KRAS* Degradation and Proliferation 46

Table S3b. Data Summary Kinetic Degradation 47

Figure S7: Effect of $KRAS^{G12R}$ Depletion in Cell Lines of Interest 47

Figure S8: Whole Cell Proteomic Analysis 48

Table S4: Significantly Up- and Down-regulated Proteins in Whole Cell Proteomics Analysis 48

Figure S9. Endogenous Evaluation of the Effect of ACBI4 on HRAS and NRAS levels in Cal-62 Cells 49

Figure S10. KRAS Degradation in KP2 Cells 50

Figure S11. Representative Western Blot from MoA Experiment in Cal-62 Cells for ACBI4. 50

Figure S12. CTG Proliferation Data for ACBI4 in A375 Cell Line 50

Table S5. Crystallographic Data and Refinement Statistics 51

Figure S13. Modelling of ACBI4 Isomers 52

References 53

Methods and Materials

Protein Production

Wild-type and mutant versions of human proteins were used for all protein expression, as follows: VHL (UniProt accession number P40337), ElonginC (Q15369), ElonginB (Q15370) and KRAS G12R and G12D or G12V variant of KRAS C118S (P01116, residues 1-169, hereafter referred to as KRASG12R, KRASG12D, KRASG12V, respectively). The VCB complex was expressed and purified as described previously.[1] Biotinylated VCB was also produced as previously described.[2] Briefly, N-terminally His6-tagged VHL (54-213), Elongin C (17-112) and Elongin B (1-104) (Addgene ID 204500 & 204501) were co-expressed in E. coli and the complex isolated by Ni-affinity chromatography. The His6-tag was removed using TEV protease, and the complex was further purified by anion exchange and size-exclusion chromatography (SEC). N-terminally His6-tagged KRASG12D/G12R/G12V was expressed and purified as described previously.[3] In brief, the protein was overexpressed in E. coli in Terrific Broth (TB) supplemented with 0.2 mM IPTG for expression induction. Expression was performed overnight at 18 °C. The protein was then purified by Ni-affinity chromatography, followed by His6-tag removal using TEV protease, another round of Ni-affinity chromatography to remove the His6-tag and the uncleaved protein, and SEC. KRAS G12D/G12R proteins were used ether as the GDP-bound form or underwent nucleotide exchange (see below) for generation of GppCp-bound forms.

C-Avi KRAS G12D/G12R were expressed in E. coli OverExpress C41(DE3) (Merck) cells in Terrific Broth (TB) media, induced with 0.2 mM IPTG at 21 °C for 22 h. The cells were harvested by centrifugation and stored at −20 °C. The cells were lysed by sonication in lysis buffer (20 mM Tris, 500 mM NaCl, 5 mM imidazole, 1 mM TCEP, 5 mM $MgCl_2$, 0.5 % 3-[(3-cholamidopropyl) dimethylammonio]-1-propanesulfonate (CHAPS), pH 7.5) supplemented with the protease inhibitors (cOmplete, EDTA-free, Merck). Cell debris was removed by centrifugation for 45 min at 13500 rpm in a JA14 rotor (Beckman) at 4°C. Recombinant KRAS was loaded onto Ni-NTA resin (Qiagen) by incubation for 2 h on ice. The slurry was loaded into a column and washed extensively with lysis buffer. The additional washing step was performed with 35 mM imidazole, and the KRAS protein was eluted with 300 mM imidazole in lysis buffer. The TEV protease cleavage and GDP exchange (1 mg GDP per 20 mg eluted protein) were performed overnight at 4 °C. The sample was then desalted into the lysis buffer and the cleaved His-tag was removed by passing the protein solution through a Ni-NTA resin. The buffer was exchanged into SEC buffer (20 mM Tris; 150 mM NaCl; 2 mM TCEP; 2 mM $MgCl_2$; pH 7.5) using centrifugal filter devices (Amicon cutt off 10 kDa, Merck). Protein was diluted to 40 μM and biotinylated overnight at 4 °C in reaction with 5 mM $MgCl_2$, 2 mM ATP, 1 μM GST-BirA and 150 μM D-Biotin followed by the incubation with additional 1 μM GST-BirA and 150 μM D-Biotin for 2 h at room temperature. Then KRAS protein was further purified by passing the protein solution through GSTrap (to remove GST-BirA) and by gel filtration chromatography (HiLoad 75S, GE Healthcare) pre-equilibrated in SEC buffer. The purity of the KRAS preparations was checked by SDS-PAGE electrophoresis and the pure fractions were pooled. The GCP-bound form was generated by nucleotide exchange protocol (below).

Nucleotide exchange

Nucleotide exchange was performed using FastAP Thermosensitive Alkaline Phosphatase (Thermoscientific) in 2M $(NH_4)_2SO_4$, 0.01M $ZnCl_2$, pH 7.5 buffer. 1U of the enzyme and 2 molar excess of guanylyl 5‘-(β,γ-methylenediphosphonate) (GppCp; Abcam) was used per 1 mg of KRAS G12D/G12R protein and incubated overnight at 4 °C. Proteins were further purified using HiLoad

Superdex 75 16/600 column pre-equilibrated in 20 mM Tris(hydroxymethyl) aminomethane (Tris), 100 mM NaCl, 2 mM Tris(2-carboxyethyl) phosphine hydrochloride (TCEP), 2 mM $MgCl_2$; pH 7.5 buffer.

SPR

Surface plasmon resonance experiments were performed on Biacore 8K instruments (Cytiva) at 20 °C with the sensor chips pre-coated with streptavidin (Xantec SAD200M), conditioned with 50 mM NaOH, 1M NaCl buffer followed by 6M guanidine-HCl; pH 2.0 (according to manufacturer's protocol). The immobilisation of KRAS proteins was performed in KRAS SPR running buffers (20 mM Tris, 200 mM potassium chloride, 2 mM magnesium chloride, 2 mM TCEP, 0.005% Tween20, pH 8.0, 2% DMSO) supplemented with 10 µM Guanosine 5'- diphosphate (GDP) or 10 µM GppCp depending on KRAS G12D/G12R nucleotide loading state. The immobilisation was set for target level of 600 RU or 30 RU (50 nM or 1.25 nM biotinylated KRAS concentration used) for binary and ternary measurements respectively. The single cycle kinetic experiments were performed with 150 s association and 5000 s dissociation time with 50 µl/min flow rate. The compounds were diluted in running buffer and injected over the immobilized target proteins with 6 concentrations of 3-fold dilution series (ranging from 0.041-10 to 20.6-5000 nM for KRAS G12D or 0.21-50 to 82.3 – 20000 nM for KRAS G12R depending on K_D estimation). For ternary complex measurements experiments were run in the presence of 10 µM VCB during the injection phase. Sensorgrams from reference surfaces and blank injections were subtracted from the raw data before data analysis using Biacore Insight software (5.0.18.22102). Affinity and binding kinetic parameters were determined by using a 1/1 interaction model.

VCB immobilisation was performed in VCB SPR running buffer (20 mM HEPES, pH 8.0, 200 mM potassium chloride, 2 mM magnesium chloride, 0.005% Tween20, 2% DMSO supplemented with 10 µM GDP or 10 µM GCP) aiming for target 160-200 RU (4-5 nM biotinylated VCB concentration used) for ternary measurements. The single cycle kinetic experiments and data analysis were performed as described above with concentration range changing from 0.041-10 to 4.12-1000 nM, in the presence of 5 µM $KRAS^{G12D}$-GDP or $KRAS^{G12D}$- GCP during the injection phase.

Protein Crystallography

Data relating to **1** $KRAS^{G12V}$ GDP were previously disclosed.[4]

VCB, compound **1** and $KRAS^{G12R}$ GCP were mixed in a 1:1.1:1.5 stoichiometric ratio in 20 mM HEPES (pH 8.0), 100 mM sodium chloride, 1 mM TCEP, 2% DMSO, 1 mM GCP and incubated for 30 min on ice before purification by size exclusion chromatography (SEC) to isolate the formed ternary complex. The eluted complex was concentrated to a final concentration of approximately 5-10 mg/mL. The drops were prepared by combining 200 nL of the ternary complex with 200 nL of well solution (100 mM Bis Tris pH 7.3, 300 mM trisodium citrate, 20% PEG 3350) and crystallized at 4 °C using the hanging-drop vapor diffusion method. Crystals were grown then cryoprotected by addition of ~500 nL well solution supplemented with 20% (v/v) glycerol to the crystallization drop followed by harvesting and flash cooling in liquid nitrogen.
VCB, compound **3** and $KRAS^{G12V}$ GDP were mixed in a 1:1.2:1 stoichiometric ratio in 20 mM HEPES (pH 8.0), 100 mM sodium chloride, 1 mM TCEP, 2% DMSO, 1 mM GDP and incubated for 30 min on ice. The complex was diluted to 6 mg/mL by addition of buffer. The drops were prepared by combining 200 nL of the ternary complex with 200 nL of well solution (200 mM magnesium

chloride, 100 mM Tris pH 7, 10% w/v polyethylene glycol 8000) and crystallized at 4 °C using the sitting-drop vapor diffusion method. Crystals were grown for 19 days then cryoprotected by addition of ~500 nL well solution supplemented with 20% (v/v) glycerol to the crystallization drop followed by harvesting and flash cooling in liquid nitrogen.

VCB, compound **3** and $KRAS^{G12R}$ GCP were mixed in a 1:1.1:1.5 stoichiometric ratio in 20 mM HEPES (pH 8.0), 100 mM sodium chloride, 1 mM TCEP, 2% DMSO, 1 mM GCP and incubated for 30 min on ice before purification by size exclusion chromatography (SEC) to isolate the formed ternary complex. The eluted complex was concentrated to a final concentration of approximately 5-10 mg/mL. The drops were prepared by combining 200 nL of the ternary complex with 200 nL of well solution (100 mM Bis Tris pH 7.3, 166.7 mM trisodium citrate, 17% PEG 3350) and crystallized at 4 °C using the sitting-drop vapor diffusion method. Crystals were grown then cryoprotected by addition of ~500 nL well solution supplemented with 20% (v/v) glycerol to the crystallization drop followed by harvesting and flash cooling in liquid nitrogen.

VCB, ACBI4 and $KRAS^{G12D}$ GDP were mixed in a 1:1.2:1 stoichiometric ratio in 20 mM HEPES, 100 mM sodium chloride, 1 mM TCEP, 0.5 mM GDP and incubated for 30 min on ice before purification by size exclusion chromatography (SEC) to isolate the formed ternary complex. The eluted complex was concentrated to a final concentration of 7 mg/mL. The drops were prepared by combining 200 nL of the ternary complex with 200 nL of well solution (0.2 M Sodium sulfate, 15% w/v Polyethylene glycol 3350, 0.1 M BIS-TRIS propane pH 7.8) before micro seeding with 20x diluted seeds (0.2 M ammonium sulfate 0.1 M sodium HEPES 7.5, 25 % w/v PEG 3350). Crystals were grown at 20 °C for 19 days then harvested, cryoprotected in a well solution supplemented with 25% (v/v) glycerol, followed by flash cooling in liquid nitrogen.

VCB, ACBI4 and $KRAS^{G12R}$ GCP were mixed in a 1:1.2:1 stoichiometric ratio in 20 mM HEPES (pH 8.0), 100 mM sodium chloride, 1 mM TCEP, 0.25 mM GCP and incubated for 30 min on ice before purification by size exclusion chromatography (SEC) to isolate the formed ternary complex. The eluted complex was concentrated to a final concentration of 7.8 mg/mL. The drops were prepared by combining 200 nL of the ternary complex with 200 nL of well solution (0.14 M ammonium sulfate, 25% w/v polyethylene glycol 3350, 0.1 M BIS-TRIS pH 5.8) before micro seeding with 20x diluted seeds (0.26 M ammonium sulfate, 25% w/v polyethylene glycol 3,350, 0.1M BIS-TRIS pH 5.5). Crystals were grown at 20°C for 19 days then harvested, cryoprotected in a well solution supplemented with 25% (v/v) PEG 400, followed by flash cooling in liquid nitrogen.

Diffraction data for the ternary complex crystals were collected at beamline X10SA at the Swiss Light Source, Switzerland at a wavelength of 1.0 Å or at beamline I24 at Diamond Light Source at a wavelength of 1.0 Å or 0.62 Å (for ACBI4 crystals). Images were processed using autoPROC or xia2 dials, the phase problem solved using PHASER using previously determined structures, the model built using Coot and iteratively refined using Phenix. The structures were deposited in the PDB with codes 9RK8, 9RKC, 9RKE, 9RKJ and 9RKN.

Cell Culture

Cell lines Cal-62, SW948 and Calu-6 were purchased from Leibnitz Institute DMSZ (German Collection of Microorganisms and Cell Cultures Gmbh), KP-2 was purchased from JCRB Tebu-bio. PSN-1 and GP5d were purchased from ECACC. A375 was purchased from LGC standards. NCI-H157 was purchased from 2B Scientific. GP5d HiBiT KRAS G12D cell line was purchased from Horizon Discovery. Cal-62 HiBiT KRAS G12R was generated in house using CRISPR technology to insert the HiBiT tag.

All the cell lines were verified for identity and tested for mycoplasma contamination at regular intervals. All the cell lines, except for KP-2, were cultured in DMEM, high glucose with GlutaMAX™

(Gibco) with 10% FBS in a humidified cell incubator at 37 °C and 5% CO_2. The KP-2 cell line was cultured in RPMI with 15% FBS.

Cell Line Generation

Plasmids pSpCas9 BB-2A-GFP (PX458) containing gRNA, Cas9 and GFP sequences and pUC67 plasmid containing ssDNA donor template were purchased from Genscript. Cal-62 cells were plated at 5x10^5 cells in a 6-well plate and left to adhere overnight. The next day, the transfection plasmids (0.5 µg of each gRNA plasmid and 0.5 µg of donor template) were diluted in Opti-MEM, combined with Lipofectamine solution in Opti-mem according to the manufacturer's protocol and incubated for 20 min at RT. The mixture was added to the cells and incubated for 24 h. The next day the media was exchanged, and the cells were left to incubate for another 24 h. GFP positive cells were single-cell sorted into 96-well plates using fluorescence-activated cell sorting (FACS) at the Flow Cytometry and Cell Sorting Facility (University of Dundee). Sorted cells were left to grow in conditioned media supplemented with 20% FBS for 3 weeks. Formed colonies were tested for the expression of HiBiT KRAS using the Nano-Glo® HiBiT Lytic system (Promega). The positive clones were additionally tested for insertion of the HiBiT tag by western blotting for KRAS (mouse anti-KRAS, LsBio) and HiBiT (mouse anti-HiBiT, Promega) and the insert was further confirmed by Sanger sequencing.

gRNA1: 5' CTGAATTAGCTGTATCGTCA 3', gRNA2: 5' GAATATAAACTTGTGGTAGT 3', gRNA 3: 5' AAACTTGTGGTAGTTGGAGC 3'

template DNA: 5' caatccagctt tatttgacac tcattctctc aactctcatc tgattcttac tgttaatatt tatccaagag aactactgcc atgatgcttt aaaagttttt ctgtagctgt tgcatattga cttctaacac ttagaggtgg gggtccacta ggaaaactgt aacaataaga gtggagatag ctgtcagcaa cttttgtgag ggtgtgctac agggtgtaga gcactgtgaa gtctctacat gagtgaagtc atgatatgat cctttgagag cctttagccg ccgcagaaca gcagtctggc tatttagata gaacaacttg attttaagat aaaagaactg tctatgtagc atttatgcat ttttcttaag cgtcgatgga ggagtttgta aatgaagtac agttcattac gatacacgtc tgcagtcaac tggaattttc atgattgaat tttgtaaggt attttgaaat aatttttcat ataaaggtga gtttgtatta aaaggtactg gtggagtatt tgatagtgta ttaaccttat gtgtgacatg ttctaatata gtcacatttt cattattttt attataaggc ctgctgaaa atgATGGTGAGCGGCTGGCGGCTGTTCAAGAAGATTAGCact gaGtaCaaGctCgtAgtagttggag ctCgtggc gta ggc aag agt gcc ttA acAatCcaActaattcagaatcat tttgtggacg aatatgatcc aacaatagag gtaaatcttg ttttaatatg catattactg gtgcaggacc attctttgat acagataaag gtttctctga ccattttcat gagtacttat tacaagataa ttatgctgaa agttaagtta tctgaaatgt accttgggtt tcaagttata tgtaaccatt aatatgggaa ctttactttc cttgggagta tgtcagggtc catgatgttc actctctgtg cattttgatt ggaagtgtat ttcagagttt cgtgagaggg tagaaatttg tatcctatct ggacctaaaa gacaatcttt ttattgtaac ttttattttt atgggtttct tggtattgtg acatcatatg taaaggttag atttaattgt actagtgaaa tataattgtt tgatggttga tttttttaaa cttcatcagc agtattttcc tatcttcttc tcaacattag agaacctaca actaccggat aaattttaca aaatgaatta tttgcctaag gtgtggttta tataaaggta ctattaccaa ctttaccttt gctttgttgt catttttaaa tttactcaag gaaatactag gatttaaaaa aaaattcctt 3'

Degradation Assays

Degradation via Detection of Split Nanoluciferase (HiBiT)

To assess PROTAC-mediated degradation of HiBiT-tagged KRAS constructs, cells were seeded at 3000 cells per well in culture medium into white bottom opaque 384-well plates (Revvity cat no. 6007689). Plates were incubated at 37 °C, 5% CO_2 in a humidified incubator over night to allow the cells to adhere. Test compounds (10 mM stock in DMSO) were added at logarithmic dose series using an Echo acoustic liquid handler (Labcyte), normalizing for added DMSO. Plates were further incubated at 37 °C for 4 and 24 h. Following incubation, 20 µL per well of Promega Nano-Glo® HiBiT lytic detection reagent mix (Promega Nano-Glo® HiBiT Lytic Detection System

#N3050), prepared according to the manufacturer´s instructions in the kit, were added. To allow for adequate cell lysis, plates were incubated on an orbital shaker for 15 min. Upon completion of cell lysis, luminescence was measured using a Glomax® Discover plate reader (Promega). Luminescence levels were normalized by the values obtained with DMSO-treated samples and plotted as percent of DMSO control. DC_{50} and Dmax values were computed using a four parametric logistic model in GraphPad Prism (version 10.4.1)

Kinetic Degradation

ViaScript™ LgBiT mRNA delivery system (Promega) was used to insert LgBiT in the cell. LgBiT mRNA was prepared according to the manufacturer's protocol. Cal-62 HiBiT KRAS cells were prepared at 5×10^5 cells/mL in a T75 flask and transfected with the mRNA mixture according to the protocol. The next day, the cells were re-plated in white 96-well tissue culture plates (Thermo) at a density of 5×10^4 cells per well in 100 µL of growth medium and incubated overnight at 37 °C, 5 % CO_2. The following day, 90 µL of CO_2-independent medium (Gibco) containing a 1× concentration of Endurazine™ (Promega) was added to each well and incubated at 37 °C, 5% CO_2, for 2.5 h to allow luminescence to equilibrate before addition of each compound. Plate lids were removed and replaced with a Breathe-Easy® sealing membrane (MilliporeSigma). Plates were read before the compounds were added (t_0) and afterwards every 5 min for a period of 18 h on the GloMax® Discover (Promega) set to 37 °C. Data was normalised to t_0 timepoint and to DMSO control and plotted in GraphPad Prism (10.4.1). Degradation rate was calculated by fitting the linear part of each kinetic degradation curve to the simple linear regression equation, where the slope is degradation rate (min^{-1}). Degradation rate was plotted against individual concentrations and fitted to the one phase association equation in GraphPad Prism:

Y=Y0 + (Plateau-Y0)*(1-exp(-K*x)), where K = degradation rate in units of μM^{-1}.

Parameters plateau (min^{-1}) and Tau (µM) were extracted from the best-fit to compare the compounds according to the degradation rate efficiency.

Retroviral Transduction of HiBiT-tagged KRAS Mutants

The therapeutically relevant mutant KRAS constructs (WT, A146P, A146T, A146V, G12A, G12C, G12D, G12R, G12V, G13C, G13D, G13V, Q61E, Q61H, Q61K, Q61L, Q61P, Q61R) were obtained by site directed mutagenesis using a KRAS4B WT cDNA construct as a template at Genscript (https://www.genscript.com). GP5d cells (ECACC No. 95090715) expressing transgenic murine Slc7a1 to allow for transduction ecotropic lentiviral particles (Takarabio Cat. No. 631278) were transduced with lentiviral vectors expressing mutant KRAS4B cDNA under control of a CMV promoter. Stably transduced cells were selected using a neomycin selectable marker encoded on the construct.

Western Blot

5×10^5 GP5d, 5×10^5 Cal-62, 3×10^5 Calu-6 cells in 2 mL/well were seeded into 6-well plates 24 hours before treatment. 2×10^5 KP2 cells in 1mL/well were seeded into 12-well plates 24 hours before treatment. Cells were treated for 4 or 18 hours as indicated, washed with PBS and lysed with lysis buffer (1% Triton X-100, 150 mM NaCl, 1 mM EDTA, 50 mM Tris pH 7.4, protease inhibitor cocktail (Roche), 50 units/mL benzonase nuclease (Sigma). The lysates were cleared by centrifugation at 4 °C, at 15800 × *g* for 10 min. and the supernatants stored at −20 °C. Protein concentration was determined by a BCA assay (Pierce) and the absorbance at 562 nm measured by spectrophotometry on a plate reader (BMG Labtech PHERAstar). Samples were separated by

SDS-PAGE using 20 µg of protein per well of NuPAGE Novex 4-12% BIS-TRIS gels (Invitrogen) and transferred to 0.2 µm pore nitrocellulose membrane (Amersham) using iBlot3 (Invitrogen). Western blot images were obtained through detection of mouse anti-pan-KRAS (1:1 000, LsBio-C175665) antibody with donkey anti-mouse IRDye 800CW secondary antibody (1:10 000, LICOR #926-32212), rabbit anti-NRAS (1:1000, Proteintech 18296-1-AP) and rabbit anti-HRAS (1:1000, Proteintech 18295-1-AP) antibodies with donkey anti-rabbit IRDye 800CW secondary antibody (1:5000, LICOR #926-32213), hFAB rhodamine anti-GAPDH (1:10 000, BioRad) and hFAB rhodamine anti-Actin (1:10 000, BioRad) using a ChemiDoc MP imaging system (Bio-Rad). Western blots were quantified using Image Studio Lite (Licor, version 5.2) with normalization to loading control and DMSO and further analyzed using GraphPad Prism (version 10.2.0) and ImageJ (Fiji) and Image Lab (Bio-Rad, version 6.2)

CRISPR Depletion Experiments

CRISPR-Cas9 depletion experiments were conducted as described previously.[5] Target cell lines of choice were transduced with lentiviral Cas9 constructs harboring puromycin resistance cassettes using routine methods. Puromycin resistant cell pools were then transduced with guide RNA constructs inserted downstream of a U6 promoter in a lentiviral vector that also expresses GFP as a marker. Viability of gRNA and Cas9 expressing cells was monitored by following the fraction of GFP positive cells in the transduced pools in the presence of puromycin by FACS. Results are stated in percent relative to the GFP+ fraction on day three post transfection. The following gRNA sequences were used to deplete the indicated target proteins. POLR2A_e10.1 (positive control) 5‘-GTA CAA TGC AGA CTT TGA CG-3‘; negative control 5‘-GAT ACA CGA AGC ATC ACT AG-3‘; KRAS_GTPase_#4 5’-CAA TGA GGG ACC AGT ACA TG-3’; KRAS_GTPase_#5 5’-TCT CGA CAC AGC AGG TCA AG-3’; KRAS_GTPase_#6 5’- CCT CCC CAG TCC TCA TGT AC-3’.

Anti-Proliferation

Cal-62 WT, A375 and Calu-6 were seeded at density of 1000 cells per well in culture medium into white bottom opaque 384-well plates (Revvity cat no. 6007689). Plates were incubated at 37 °C, 5% CO_2 in a humidified incubator over night to allow the cells to adhere. Test compounds (10 mM stock in DMSO) were added at logarithmic dose series using an Echo acoustic liquid handler (Labcyte), normalizing for added DMSO. Plates were further incubated at 37 °C for 6 days. Following incubation, 10 µL per well Promega CellTiter-Glo® detection reagent mix (G7570) prepared according to the manufacturer’s instructions in the kit, were added. To allow for adequate cell lysis, plates were incubated on an orbital shaker for 5 min, then incubated at RT for 15 min. Upon completion of cell lysis, luminescence was measured using a Glomax® Discover plate reader (Promega). Luminescence levels were normalized by the values obtained with DMSO-treated samples and plotted as percent of DMSO control. IC_{50} values were computed using a four parametric logistic model in GraphPad Prism.

Proteomics

5 × 10^6 Cal-62 cells were seeded on a 100 mm plate 24 hours before treatment. Cells were treated with ACBI4 and negative control in triplicates at 1 µM for 6 hours. Then, the cells were washed twice with 10 mL of cold PBS and lysed in 5% (w/v) SDS in 100 mM Tris at pH 8.5. The lysates were pulse sonicated briefly and then centrifuged at 15000 × g for 15 min. Samples were quantified using a micro-BCA protein assay kit (Thermo Fisher Scientific). 300 µg proteins of each sample were reduced with DTT 20 mM, alkylated with iodoacetamide 40 mM and digested with trypsin 1:4 ratio using the modified S-TRAP mini (ProtiFi) protocol. Peptide quantification was done using

Pierce™ Quantitative Fluorometric Peptide Assay and an equal amount of peptides from each sample were labelled using a TMTpro 16-plex Label Reagent Set (Thermo Fisher Scientific, A44522) as per the manufacturer's instructions. The samples were then pooled and desalted using a 7 mm, 3 mL C18 SPE cartridge column (Empore, 3M).

LCMS method Q Exactive HF Hybrid Quadrupole-Orbitrap Mass Spectrometer: The pooled and desalted sample was fractionated using high pH reverse-phase chromatography on an XBridge peptide BEH column (130 Å, 3.5 µm, 2.1 × 150 mm, Waters) on an Ultimate 3000 HPLC system (Thermo Scientific/Dionex). Buffers A (10 mM ammonium formate in water, pH 9) and B (10 mM ammonium formate in 90% acetonitrile, pH 9) were used over a linear gradient of 2% to 100% buffer B over 80 min at a flow rate of 200 µL/min. 80 fractions were collected using an aWPS-3000 FC auto-sampler (Thermo Scientific) before concatenation into 20 fractions based on the UV signal of each fraction. All the fractions were dried in a Genevac EZ-2 concentrator and resuspended in 1 % formic acid for MS analysis. The fractions were analyzed sequentially on a Q Exactive HF Hybrid Quadrupole-Orbitrap Mass Spectrometer (Thermo Scientific) coupled to a Dionex Ultimate 3000 RS (Thermo Scientific). Buffers A (0.1% formic acid in water) and B (0.1% formic acid in 80% acetonitrile) were used over a linear gradient from 5% to 35% buffer B over 125 min, and then from 35% buffer B to 98% buffer B in 2 min at a constant flow rate of 300 nL/min. The column temperature was 50 °C. The mass spectrometer was operated in data dependent mode with a single MS survey scan from 335-1600 m/z followed by 15 sequential m/z dependent MS2 scans. The 15 most intense precursor ions were sequentially fragmented by higher energy collision dissociation (HCD). The MS1 isolation window was set to 0.7 m/z and the resolution set at 120000. MS2 resolution was set at 60000. The AGC targets for MS1 and MS2 were set at 3 × 106 ions and 1 × 105 ions, respectively. The normalized collision energy was set at 32%. The maximum ion injection times for MS1 and MS2 were set at 50 ms and 200 ms respectively. The mass accuracy was checked before the initiation of sample analysis. MaxQuant software (version 2.0.3.0) coupled to the Andromeda search engine was used for the identification and quantification of the total proteome TMT peptides.[6] Raw MS data files for all 20 fractions were loaded and queried against the Human Uniprot database. The MaxQuant parameters were set as follows: enzyme used Trypsin/P; maximum number of missed cleavages equal to two; precursor mass tolerance equal to 10 ppm; fragment mass tolerance equal to 20 ppm. Variable modifications: oxidation (M), identifier (MW), acetyl (N-term), deamidation (NQ), Gln -> pyro-Glu (Q N-term). Fixed modifications: carbamidomethyl (c), TMT of lysine and N-terminal. The data was filtered by applying a 1% false discovery rate followed by exclusion of proteins with less than one unique peptide. The isotopic impurity of the TMTpro 16-plex batch were specified in the configuration of the TMT modification to enable MaxQuant to apply the automatic corrections of TMT intensities. The proteingroups.txt file generated as output table from MaxQuant processing was loaded into Perseus for statistical analysis.[7] The dataset was filtered by removing potential contaminants, reverse peptide s, only identified by site peptides. Then, the replicates were grouped based on their annotation to DMSO, ACBI4, Negative control. The TMT reporter ions corrected intensities values were transformed in log2 scale and normalized by the median for each sample independently. Then, for pairwise comparisons, a Student-T test was performed using FDR<0.05 and s0=0.1. The analysed data were used to generate volcano plots using Graph Prism software (Version 9). Significant changes were classified by p<0.005 and a fold change greater than 1.4-fold. The mass spectrometry proteomics data generated in Cal-62 WT have been deposited to the ProteomeXchange Consortium via the PRIDE (*49*) partner repository with the dataset identifier PXD065163.

Chemical Synthesis

General Information

Commercially available dry solvents were obtained from Sigma Aldrich. All reagents, unless otherwise noted, were commercially available and purchased from Sigma Aldrich, Combi Blocks, ABCR, Fluorochem, Activate or Enamine, at least 95% pure and used without further purification. All reactions were carried out under a nitrogen atmosphere. Normal phase TLC was carried out on pre-coated silica plates (Kieselgel 60 F254, BDH) with visualization via UV light (UV 254 and/or 365 nm) and/or basic potassium permanganate solution. Isolute® phase separator columns from Biotage were used. Flash column chromatography was performed using either a Teledyne Isco Combiflash Rf or Rf200i, or a Biotage Isolera One with prepacked Redisep RF normal phase disposable columns. Reverse phase chromatography was carried out using Biotage SNAP-C18 columns or RediSep Rf Reversed Phase C18 Columns. Strong cation exchange (SCX) chromatography was carried out using Biotage Isolute SCX-2 columns. NMR Spectra were recorded on Bruker 400 MHz or 500 MHz spectrometers as specified. Chemical shifts are quoted in ppm and referenced to the residual solvent signals: ^{1}H NMR δ (ppm) = 7.26 ($CDCl_3$-d), 5.32 (CD_2Cl_2) or 3.31 (MeOD-d_4). Signal splitting patterns are described as singlet (s), doublet (d), triplet (t), quartet (q), quintet (quin.), multiplet (m), broad (br) or a combination thereof. Coupling constants (J) are measured in Hertz (Hz). Diastereomeric ratios (dr) were calculated using the ratios of NMR integrals. Chiral SFC was carried out by Reach Separations Ltd., using a Sepiatec SFC systems equipped with a Lux A1 column (21.2 mm x 250 mm, 5 um particle size). Samples were eluted with an isocratic gradient of 40:60 EtOH:CO_2 (0.2% v/v NH_3) over 10 min at a flow rate of 50 mL/min with an oven temperature of 40 °C. Diastereomeric mixture was dissolved in EtOH at 6 mg/mL and injected 16 times at 500 uL.

Abbreviations

aq. for aqueous, Boc for *N-tert*butyloxycarbonyl, DCE for 1,2-dichloroethane, DCM for dichloromethane, DMA for *N*,*N*-dimethylacetamide, DME for dimethoxyethane, DMF for *N*,*N*-dimethylformamide, DIPEA for N,N-diisopropylethylamine, DMSO for dimethylsulfoxide, dppf for 1,1'-bis(diphenylphosphino)ferrocene, Et_3N for triethylamine, EtOAc for ethyl acetate, HATU for 1-[bis(dimethylamino)methylene]-1*H*-1,2,3-triazolo[4,5-b]pyridinium 3-oxide hexafluorophosphate, HOAt for 1-hydroxy-7-azabenzotriazole, MeOH for methanol, MsCl for methanesulfonyl chloride, TBAF for tetra-*N*-butylammonium fluoride, TBS for tert-butyldimethylsilyl, TBDPS for *tert*-butyldiphenylsilyl.

Analytical MS Methods and instrumentation

HRMS data

HRMS data was recorded using an LTQ Orbitrap XL (Thermo Scientific) coupled with a Triversa Nanomate Nanospray ion source (ADVION Bioscience Inc.). The mass calibration was performed using the Pierce LTQ Velos ESI positive ion calibration solution from Thermo Scientific (Product Nr. 88323). MS parameters: The scan window was set to 50–400 amu with a maximum injection time of 500 ms and 1 microscan. Resolution of the Orbitrap was 60000 with a mass accuracy ≤ 5 ppm. The ion mode set to positive with a capillary temperature 200 °C and voltage of 60 eV. The tube lens potential was set to 110 eV. 12 NanoESI voltage was 1.45 kV and the N_2 gas pressure set to 0.45 psi. Total sample volume was 5 μL and the acquisition time was 0.4 sec, with 10 scans of averaging per spectrum. Sample dilution: 10 mM DMSO stock solution was diluted to 1:200 in 50% MeOH +0.01% formic acid.

LCMS

Liquid chromatography-mass spectrometry (LCMS) was carried out on a Shimadzu HPLC/MS 2020 equipped with a Hypersil Gold column (1.9 µm particle size, 50 × 2.1 mm), photodiode array detector and ESI detector. Samples were eluted with either a 3 min or 5 min gradient of 5–95% acetonitrile:water containing 0.1% formic acid at a flow rate of 0.7 mL/min.

HPLC

Preparative HPLC was performed on a Waters Prep 150 LC system with a Waters XBridge C18 column (100 mm x 19 mm; 5 µm particle size) and a gradient of 5% to 95% acetonitrile in water over 20 minutes and a flow rate of 25 mL/min, with ammonia in the aqueous phase.

Experimental Procedures

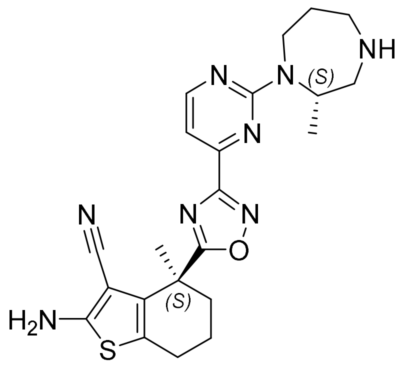

S1

KRAS binder **S1** was synthesised according to literature procedures[4]

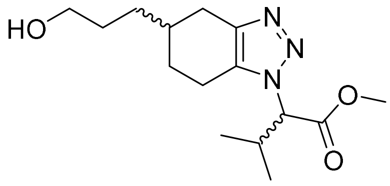

S2

Intermediate **S2** was synthesised by Aragen Life Sciences Ltd using the following route:

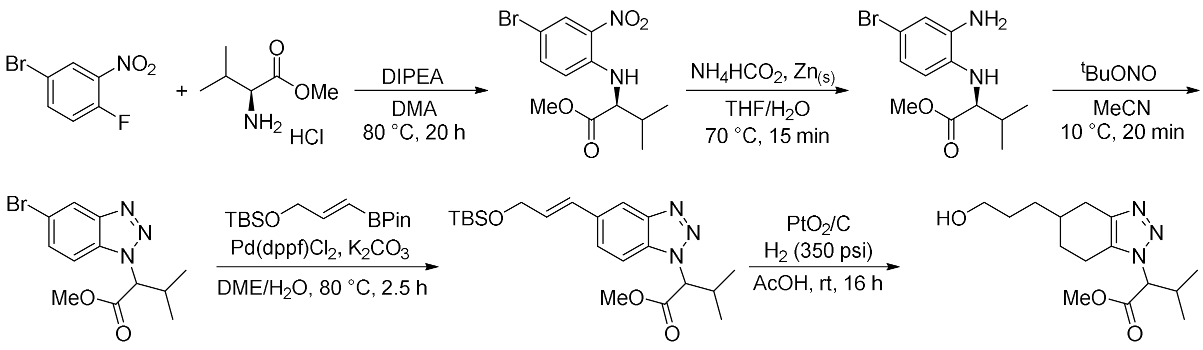

S2

General procedure A: ester hydrolysis and amide coupling

To a solution of ester (1.0 eq) in EtOH or MeOH (0.1 M) is added 2 M $NaOH_{(aq)}$ (2.0 eq). The resulting solution is stirred and heated at 50 °C for 1 h. The solution is then either acidified to pH 1 by the addition of 1 M $HCl_{(aq)}$ and concentrated under reduced pressure to give the crude acid which is

used in next step without further purification, or acidified by the addition of formic acid, concentrated under reduced pressure and purified by reverse phase chromatography to give the corresponding acid product.

To a solution of acid (1.0 eq), amine (1.2 eq) and DIPEA in DMF or DMSO (0.1 M) is added HOAt and HATU. The resulting solution is stirred at rt for 30 min. The solution is quenched with saturated $NaHCO_{3(aq)}$. The aqueous layer is extracted with DCM x3. The combined organic layers are washed with brine, dried (Na_2SO_4) and concentrated under reduced pressure to give the crude product.

Ethyl 6-((*tert*-butyldiphenylsilyl)oxy)-3-oxohexanoate **S4**

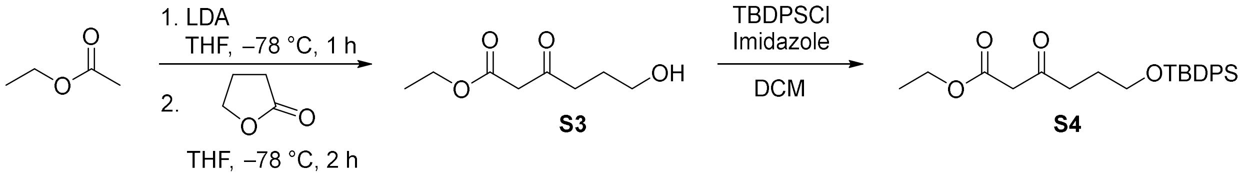

n-BuLi (24.6 ml of a 2.5 M solution in hexanes, 61.5 mmol, 1.2 eq) is added to a solution of diisopropylamine (8.60 ml, 61.3 mol, 1.2 eq) in THF (90 ml) at –78 °C dropwise. The resulting solution is stirred at –78 °C for 30 min. EtOAc (6.0 ml, 61.4 mmol, 1.2 eq) is added dropwise. The resulting solution is stirred at –78 °C for 1 h. Butyrolactone (4.10 ml, 53.5 mmol, 1.0 eq) is then added and the resulting solution is stirred at –78 °C for 2 h. The reaction mixture is quenched with acetic acid (5 mL) and is allowed to warm up to rt. The reaction mixture is diluted with saturated $NaHCO_{3(aq)}$ (100 mL) and ether (100 mL). The aqueous phase is extracted with ether (2 x 50 mL). The combined organic layers are washed with saturated $NaHCO_{3(aq)}$ (50 mL), brine (50 mL), dried ($MgSO_4$) and concentrated under reduced pressure to give crude **S3** which is used in the next step without further purification.
TBDPSCl (12.0 ml, 46.1 mmol, 0.9 eq) is added dropwise to a solution of crude **S3** and imidazole (5.70 g, 83.7 mmol, 1.6 eq) in DCM (40 ml) at 0 °C. The resulting solution is allowed to warm to rt and stirred at rt for 16 h. The reaction mixture is loaded onto silica gel and purified by normal phase column chromatography (0-20% EtOAc in heptane). A 75/25 mixture of product **S4** and TBDPSCl is isolated and used as such in the next step.

^{1}H NMR (400 MHz, $CDCl_3$) δ 7.67 – 7.62 (m, 4H), 7.43 – 7.35 (m, 6H), 4.19 (q, *J* = 7.0 Hz, 2H), 3.67 (t, *J* = 6.0 Hz, 2H), 2.67 (t, *J* = 7.0 Hz, 2H), 1.98 – 1.75 (m, 2H), 1.27 (t, *J* = 7.0 Hz, 3H), 1.05 (s, 9H).

Ethyl 1-(4-((*tert*-butyldiphenylsilyl)oxy)butanoyl)cyclopropane-1-carboxylate **S5**

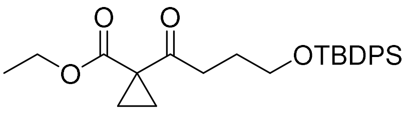

S5

S4 (14.5 g, 24.6 mmol, 1.0 eq) is dissolved in DMF (30 ml). Potassium carbonate (15.3 g, 110 mmol, 4.5 eq) is added to the reaction mixture at room temperature. The resulting reaction mixture is treated with 1,2-dibromo-ethane (3.18 ml, 36.9 mmol, 1.5 eq) over 5 minutes. The reaction mixture is vigorously stirred at rt for 16 h. The reaction mixture is diluted with ether and filtered through Celite©. The filtrate is concentrated under reduced pressure to give the crude product. Purification by normal phase column chromatography (0-30% EtOAc in Heptane) gives **S5** (8.26 g, 77% yield) as a yellow oil.

^{1}H NMR (500 MHz, $CDCl_3$) δ 7.70 – 7.66 (m, 4H), 7.47 – 7.37 (m, 6H), 4.22 (q, *J* = 7.0 Hz, 2H), 3.70 (t, *J* = 6.0 Hz, 2H), 2.99 (t, *J* = 7.5 Hz, 2H), 1.89 (tt, *J* = 7.5, 6.0 Hz, 2H), 1.48 – 1.40 (m, 4H), 1.29 (t, *J* = 7.0 Hz, 3H), 1.08 (s, 9H).

Ethyl 1-(4-hydroxybutanoyl)cyclopropane-1-carboxylate **S6**

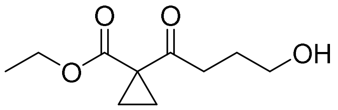

S6

TBAF (35 ml of a 1.0 M solution in THF, 35 mol, 2.5 eq) is added to a solution of **S5** (8.26 g, 14.1 mmol, 1.0 eq) in THF (70 ml) at 0 °C. The resulting solution is allowed to warm to rt and stirred at rt for 2 h. TLC shows full conversion of SM. The reaction mixture is diluted with dichloromethane, dried ($MgSO_4$) and concentrated under reduced pressure to give the crude product. Purification by normal phase column chromatography (30-50% EtOAc in heptane) gives **S6** (2.59 g, 92% yield) as a yellow oil.

^{1}H NMR (400 MHz, $CDCl_3$) δ 4.19 (q, *J* = 7.0 Hz, 2H), 3.64 (q, *J* = 6.0 Hz, 2H), 2.98 (t, *J* = 7.0 Hz, 2H), 1.93 – 1.82 (m, 2H), 1.46 (d, *J* = 4.0 Hz, 2H), 1.45 (d, *J* = 4.0 Hz, 2H), 1.27 (t, *J* = 7.0 Hz, 3H).

Ethyl 1-(4-oxobutanoyl)cyclopropane-1-carboxylate **S7**

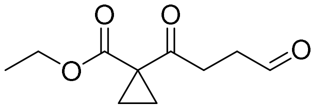

S7

To a solution of **S6** (200 mg, 1.0 mmol, 1.0 eq) in DCM (5 mL) is added Dess–Martin periodinane (508 mg, 1.20 mmol, 1.20 eq). The resulting suspension is stirred at rt for 1 h. TLC shows full consumption of SM so is a 1:1 mixture of saturated $NaHCO_{3(aq)}$ and saturated $Na_2S_2O_{3(aq)}$ is added. The resulting biphasic mixture is stirred for 30 min. The aqueous layer is extracted with DCM (2 x 10 mLL. The combined organic layers are washed with brine, dried (Na_2SO_4) and concentrated under reduced pressure to give **S7** (130 mg, 53 % yield) as a pale yellow oil.

^{1}H NMR (500 MHz, $CDCl_3$) δ 9.79 (br s, 1H), 4.21 (q, *J* = 7.0 Hz, 2H), 3.22 (br t, *J* = 6.0 Hz, 2H), 2.77 (br t, *J* = 6.5 Hz, 2H), 1.51 (d, *J* = 3.0 Hz, 2H), 1.50 (d, *J* = 3.0 Hz, 2H), 1.29 (t, *J* = 7.0 Hz, 3H).

Ethyl 1-(4-((*S*)-4-(4-(5-((*S*)-2-amino-3-cyano-4-methyl-4,5,6,7-tetrahydrobenzo[b]thiophen-4-yl)-1,2,4-oxadiazol-3-yl)pyrimidin-2-yl)-3-methyl-1,4-diazepan-1-yl)butanoyl)cyclopropane-1-carboxylate **S8**

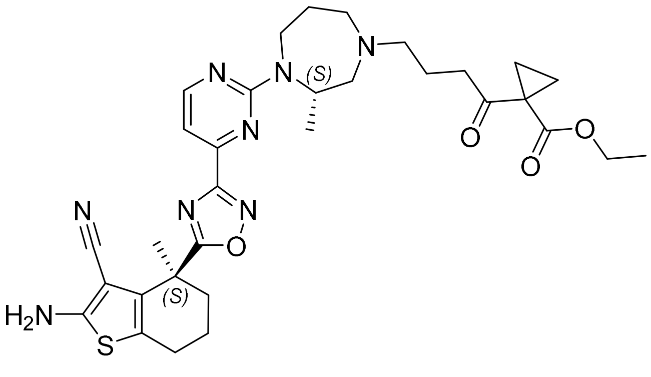

S8

To a solution of **S1** (50 mg, 0.11 mmol, 1.0 eq) and **S8** (44 mg, 0.22 mmol, 2.0 eq) in DCE (1 mL) is added STAB (118 mg, 0.56 mmol, 5.0 eq). The resulting solution is stirred at rt for 30 min. LCMS shows full conversion to desired product so it is quenched with saturated $NaHCO_{3(aq)}$. The aqueous layer is extracted with DCM (3 x 5 mL). The combined organic layers are washed with brine, dried (Na_2SO_4) and concentrated under reduced pressure to give the crude product. Purification by normal phase chromatography (0-20% DCM in MeOH), then reverse phase chromatography (5-95% 0.1 % NH_4OH in H_2O/MeCN) gives **S8** (19 mg, 24% yield) as a brown solid.

^{1}H NMR (500 MHz, $CDCl_3$) δ 8.45 (d, *J* = 5.0 Hz, 1H), 7.16 (d, *J* = 5.0 Hz, 1H), 4.91 – 4.62 (m, 3H), 4.59 – 4.29 (m, 1H), 4.21 – 4.02 (m, 2H), 3.18 – 3.08 (m, 1H), 3.04 (dd, *J* = 15.0, 5.5 Hz, 1H), 2.90 (br d, *J* = 13.0 Hz, 1H), 2.80 (t, *J* = 7.0 Hz, 2H), 2.70 – 2.29 (m, 6H), 1.97 – 1.84 (m, 7H), 1.81 – 1.55 (m, 5H), 1.38 (s, 3H), 1.23 (t, *J* = 7.5 Hz, 3H), 1.13 – 1.01 (m, 3H). MS (ESI) for $C_{32}H_{41}N_8O_4S$ $[M+H]^+$ calculated 633.30, found 633.3.

(2*S*,4*R*)-1-((*S*)-2-(1-(4-((*S*)-4-(4-(5-((*S*)-2-Amino-3-cyano-4-methyl-4,5,6,7-tetrahydrobenzo[b]thiophen-4-yl)-1,2,4-oxadiazol-3-yl)pyrimidin-2-yl)-3-methyl-1,4-diazepan-1-yl)butanoyl)cyclopropane-1-carboxamido)-3,3-dimethylbutanoyl)-4-hydroxy-*N*-((1-methyl-1*H*-indazol-6-yl)methyl)pyrrolidine-2-carboxamide **2**

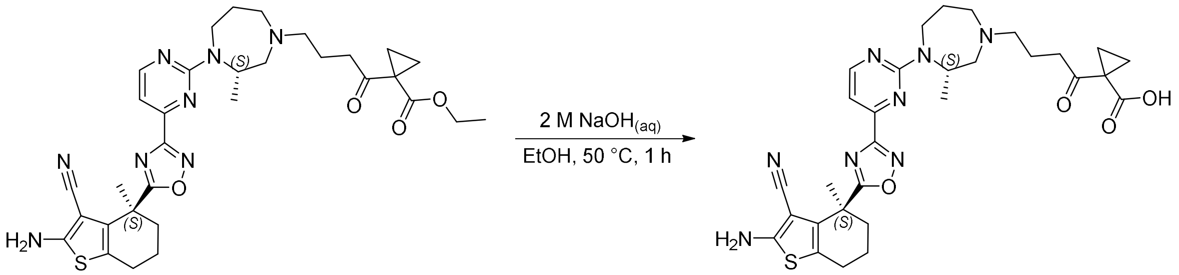

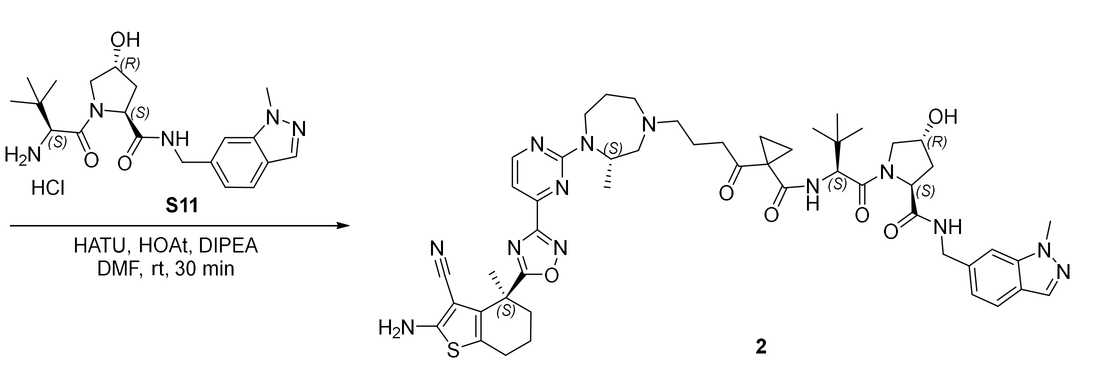

Using general procedure A, **S8** (19 mg, 0.03 mmol, 1.0 eq) and 2 M $NaOH_{(aq)}$ (30 uL, 0.06 mmol, 2.0 eq) in EtOH (0.3 ml) gives the crude product. The solution is acidified to pH 1 by the addition of 1 M $HCl_{(aq)}$ and concentrated under reduced pressure to give the crude acid **S9** which is used in the next step without further purification.

MS (ESI) for $C_{30}H_{37}N_8O_4S$ $[M+H]^+$ calculated 605.26, found 605.2.

S9, **S11** (15 mg, 0.04 mmol, 1.2 eq), HATU (17 mg, 0.05 mmol, 1.5 eq), HOAt (6 mg, 0.05 mmol, 1.5 eq) and DIPEA (31 µl, 0.18 mmol, 6.0 eq) in DMF (0.3 ml) gives the crude product. Purification by reverse phase chromatography (5-95% MeCN in 0.1 % aq. NH_4OH), then preparative HPLC gives **2** as an off white solid.

^{1}H NMR (500 MHz, $CDCl_3$)(mixtures of rotamers) δ 9.82 – 9.72 (m, 1H), 8.46 (s, 1H), 7.91 (d, *J* = 1.0 Hz, 1H), 7.60 (d, *J* = 8.0 Hz, 1H), 7.53 (br t, *J* = 6.0 Hz, 1H), 7.35 (s, 1H), 7.21 – 7.14 (m, 1H), 7.02 (dd, *J* = 8.0, 1.0 Hz, 1H), 5.02 – 4.84 (m, 2H), 4.74 (t, *J* = 8.0 Hz, 1H), 4.68 (dd, *J* = 15.0, 7.0 Hz, 1H), 4.50 (d, *J* = 14.5 Hz, 0.5H), 4.42 (s, 1H), 4.38 – 4.29 (m, 1.5H), 4.29 – 4.21 (m, 1H), 4.12 (d, *J* = 11.5 Hz, 1H), 4.05 (s, 3H), 3.60 – 3.39 (m, 2H), 3.14 – 3.04 (m, 1H), 3.01 – 2.90 (m, 1H), 2.86 – 2.79 (m, 1H), 2.67 – 2.42 (m, 5H), 2.42 – 2.27 (m, 3H), 2.16 – 1.99 (m, 3H), 1.97 – 1.84 (m, 7H), 1.75 – 1.46 (m, 7H), 1.43 – 1.31 (m, 2H), 1.08 – 1.03 (m, 3H), 0.88 (s, 9H). HRMS (ESI) for $C_{50}H_{64}N_{13}O_6S$ $[M+H]^+$ calculated 974.4823, found 974.4831

(2*S*,4*R*)-1-((*S*)-2-(1-(4-((*S*)-4-(4-(5-((*S*)-2-Amino-3-cyano-4-methyl-4,5,6,7-tetrahydrobenzo[b]thiophen-4-yl)-1,2,4-oxadiazol-3-yl)pyrimidin-2-yl)-3-methyl-1,4-diazepan-1-yl)butanoyl)cyclopropane-1-carboxamido)-3,3-dimethylbutanoyl)-4-hydroxy-*N*-(4-(4-methylthiazol-5-yl)benzyl)pyrrolidine-2-carboxamide **3**

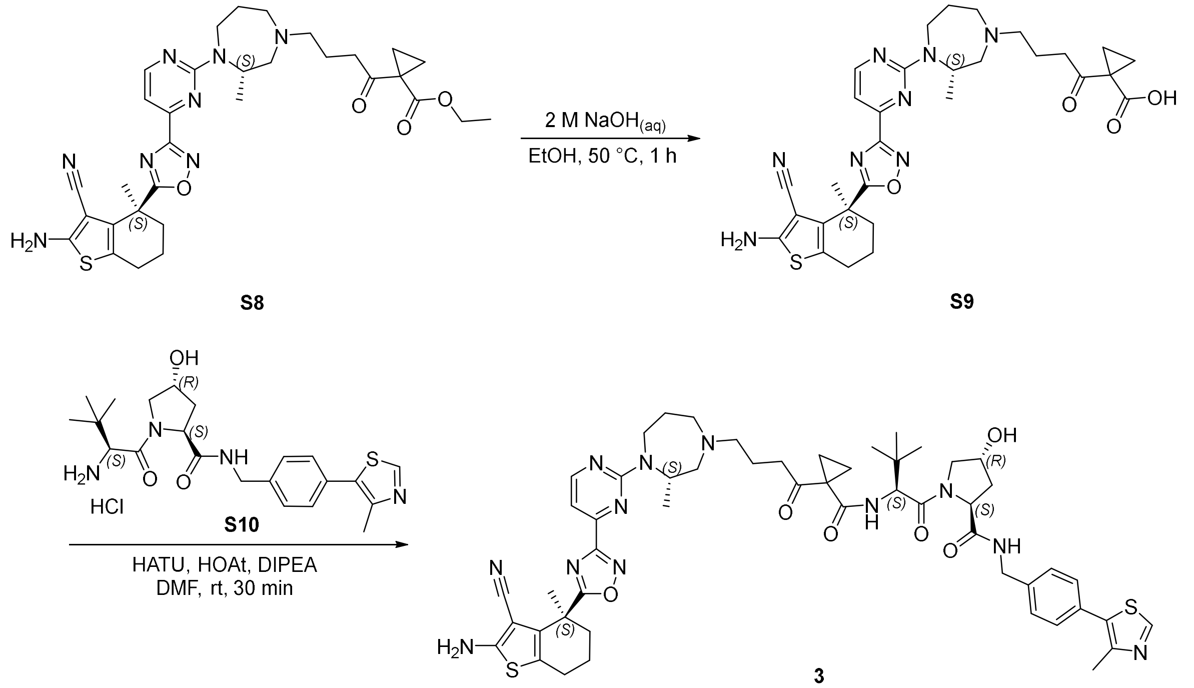

Using general procedure A, **S8** (43 mg, 0.07 mmol, 1.0 eq) and 2 M $NaOH_{(aq)}$ (70 uL, 0.14 mmol, 2.0 eq) in EtOH (0.7 ml) gives the crude product. The solution is acidified to pH 1 by the addition of 1 M $HCl_{(aq)}$ and concentrated under reduced pressure to give the crude acid **S9** which is used in next step without further purification.

MS (ESI) for $C_{30}H_{37}N_8O_4S$ $[M+H]^+$ calculated 605.26, found 605.2.

S9, **S10** (32 mg, 0.07 mmol, 1.0 eq), HATU (44 mg, 0.12 mmol, 1.7 eq), HOAt (16 mg, 0.12 mmol, 1.7 eq) and DIPEA (44 µl, 0.27 mmol, 4.0 eq) in DMF (2.0 ml) gives the crude product. Purification by reverse phase chromatography (5-95% MeCN in 0.1 % aq. NH_4OH), then preparative HPLC gives **3** as an off white solid.

^{1}H NMR (400 MHz, CD_2Cl_2) δ 9.53 (s, 1H), 8.57 (s, 1H), 8.37 (d, *J* = 4.8 Hz, 1H), 7.35 (t, *J* = 5.2 Hz, 1H), 7.30 (d, *J* = 8.4 Hz, 2H), 7.26 (d, *J* = 8.3 Hz, 2H), 7.06 (d, *J* = 4.9 Hz, 1H), 4.91 (d, *J* = 39.5 Hz, 1H), 4.77 (s, 1H), 4.60 (t, *J* = 8.1 Hz, 1H), 4.44 (dd, *J* = 15.0, 6.7 Hz, 1H), 4.33 (s, 1H), 4.27 – 4.17 (m, 2H), 3.99 (d, *J* = 11.3 Hz, 1H), 3.42 (dd, *J* = 11.5, 3.5 Hz, 1H), 3.03 (dd, *J* = 14.2, 11.7 Hz, 1H), 2.87 (d, *J* = 14.6 Hz, 1H), 2.73 (d, *J* = 12.6 Hz, 1H), 2.53 (q, *J* = 5.7 Hz, 2H), 2.47 – 2.15 (m, 8H), 2.14 – 1.82 (m, 6H), 1.80 (s, 3H), 1.68 – 1.25 (m, 10H), 0.97 (d, *J* = 6.4 Hz, 3H), 0.87 (s, 9H). HRMS (ESI) for $C_{52}H_{65}N_{12}O_6S_2$ $[M+H]^+$ calculated 1017.4591, found 1017.4723.

Methyl 3-methyl-2-(5-(3-((methylsulfonyl)oxy)propyl)-4,5,6,7-tetrahydro-1*H*-benzo[d][1,2,3]triazol-1-yl)butanoate **S12**

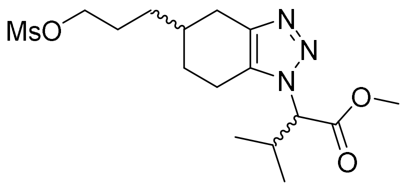

S12

To a solution of **S2** (240 mg, 0.82 mmol, 1.0 eq) and Et_3N (1.13 mL, 8.13 mmol, 10 eq) in DCM (6 mL) at 0 °C is added MsCl (0.31 mL, 4.06 mmol, 5.0 eq). The resulting solution is stirred at 0 °C for 15 min. LCMS shows full conversion to desired product so it is diluted with DCM and quenched with saturated $NaHCO_{3(aq)}$. The aqueous layer is extracted with DCM x2. The combined organic layers are washed with brine, dried ($MgSO_4$) and concentrated under reduced pressure to give the crude product. Purification by normal phase chromatography (0-5% DCM in MeOH) gives **S12** (294 mg, 97% yield) as a yellow viscous oil.

^{1}H NMR (500 MHz, $CDCl_3$)(60:40 mixture of diastereomers) δ 4.76 (d, *J* = 10.5 Hz, 0.4H), 4.72 (d, *J* = 10.0 Hz, 0.6H), 4.32 – 4.21 (m, 2H), 3.75 (s, 2H), 3.75 (s, 1H), 3.02 (s, 2H), 3.02 (s, 1H), 2.95 (dt, *J* = 16.5, 4.5 Hz, 1H), 2.91 – 2.89 (m, 0.4H), 2.85 – 2.73 (m, 1H), 2.72 – 2.67 (m, 1H), 2.60 – 2.49 (m, 0.6H), 2.41 – 2.28 (m, 1H), 2.06 – 1.97 (m, 1H), 1.93 – 1.77 (m, 3H), 1.62 – 1.46 (m, 3H), 1.10 (dd, *J* = 6.5, 3.0 Hz, 3H), 0.82 (dd, *J* = 6.5, 5.5 Hz, 3H). MS (ESI) for $C_{16}H_{28}N_3O_5S$ $[M+H]^+$ calculated 374.17, found 374.1.

Methyl 2-(5-(3-((*S*)-4-(4-(5-((*S*)-2-amino-3-cyano-4-methyl-4,5,6,7-tetrahydrobenzo[b]thiophen-4-yl)-1,2,4-oxadiazol-3-yl)pyrimidin-2-yl)-3-methyl-1,4-diazepan-1-yl)propyl)-4,5,6,7-tetrahydro-1*H*-benzo[d][1,2,3]triazol-1-yl)-3-methylbutanoate **S13**

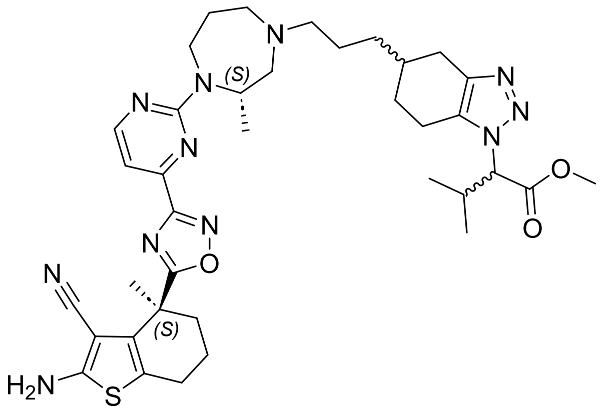

S13

To a solution of **S1** (50 mg, 0.11 mmol, 1.0 eq), **S12** (46 mg, 0.12 mmol, 1.2 eq) and KI (20 mg, 0.12 mmol, 1.2 eq) in MeCN (3.5 mL) is added $NaHCO_3$ (93 mg, 1.11 mmol, 10 eq). The resulting suspension is stirred and heated at 85 °C for 16 h. LCMS shows full conversion to desired product so the reaction mixture is partitioned between water and DCM. The aqueous layer is extracted DCM x2. The combined organic layers are washed with brine, dried (Na_2SO_4) and concentrated under reduced pressure to give the crude product. Purification by normal phase chromatography (0-10% DCM in MeOH) gives **S13** (64 mg, 78% yield) as a brown solid.

^{1}H NMR (500 MHz, $CDCl_3$) δ 8.46 (d, *J* = 5.0 Hz, 1H), 7.20 – 7.14 (m, 1H), 5.09 – 4.76 (m, 3H), 4.75 – 4.66 (m, 1H), 4.66 – 4.35 (m, 1H), 3.76 – 3.71 (m, 3H), 3.19 – 3.05 (m, 2H), 2.97 (br d, *J* = 13.0 Hz, 1H), 2.89 (br d, *J* = 17.0 Hz, 1H), 2.85 – 2.71 (m, 2H), 2.68 – 2.56 (m, 4H), 2.54 – 2.41 (m, 1H), 2.37 – 2.22 (m, 2H), 2.00 – 1.87 (m, 8H), 1.76 – 1.65 (m, 3H), 1.64 – 1.53 (m, 2H), 1.48 – 1.40 (m, 1H), 1.38 –1.30 (m, 2H), 1.14 – 1.05 (m, 6H), 0.80 (t, *J* = 6.0 Hz, 3H). MS (ESI) for $C_{37}H_{50}N_{11}O_3S$ $[M+H]^+$ calculated 728.38, found 728.4.

(2*S*,4*R*)-1-(2-(5-(3-((*S*)-4-(4-(5-((*S*)-2-Amino-3-cyano-4-methyl-4,5,6,7-tetrahydrobenzo[b]thiophen-4-yl)-1,2,4-oxadiazol-3-yl)pyrimidin-2-yl)-3-methyl-1,4-diazepan-1-yl)propyl)-4,5,6,7-tetrahydro-1*H*-benzo[d][1,2,3]triazol-1-yl)-3-methylbutanoyl)-4-hydroxy-*N*-((1-methyl-1*H*-indazol-6-yl)methyl)pyrrolidine-2-carboxamide **4**

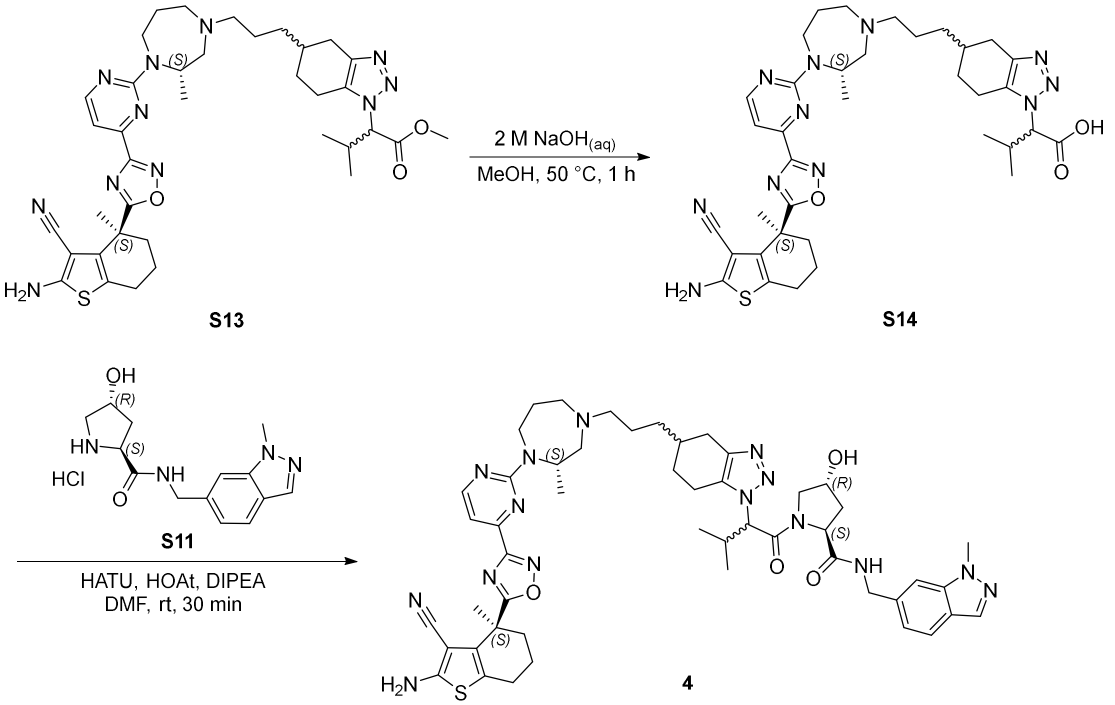

Using general procedure A, **S13** (48 mg, 0.07 mmol, 1.0 eq) and 2 M $NaOH_{(aq)}$ (70 uL, 0.14 mmol, 2.0 eq) in MeOH (0.7 ml) gives the crude product. The solution is acidified formic acid and concentrated under reduced pressure to give the crude product. Purification by reverse phase chromatography (5-95% MeCN in 0.1 % aq. formic acid) gives acid **S14** (34 mg, 70% yield) as a brown solid.

MS (ESI) for $C_{36}H_{48}N_{11}O_3S$ $[M+H]^+$ calculated 714.37, found 714.4.

S14, **S11** (17 mg, 0.05 mmol, 1.2 eq), HATU (20 mg; 0.05 mmol; 1.2 eq), HOAt (7 mg, 0.05 mmol, 1.2 eq) and DIPEA (39 µl, 0.22 mmol, 5.0 eq) in DMSO (2.0 ml) gives the crude product. Purification by normal phase chromatography (0-20% DCM in MeOH) then preparative HPLC gives a diastereomeric mixtures of **4** (27 mg, 59% yield) as an off white solid.

^1H NMR (500 MHz, $CDCl_3$)(mixtures of diastereomers) δ 8.47 – 8.40 (m, 1H), 7.93 – 7.89 (m, 1H), 7.66 – 7.58 (m, 0.9H), 7.53 – 7.50 (m, 0.1H), 7.46 – 7.38 (m, 1H), 7.32 – 7.21 (m, 0.2 H), 7.19 – 7.10 (m, 1H), 7.05 – 6.97 (m, 0.8H), 5.34 – 5.15 (m, 1H), 5.02 – 4.88 (m, 2H), 4.84 – 4.71 (m, 1H), 4.70 – 4.42 (m, 3H), 4.42 – 4.31 (m, 1H), 4.07 – 4.02 (m, 2.5H), 3.88 – 3.42 (m, 0.5H), 3.16 – 3.00 (m, 2H), 2.97 2.90 (br d, J = 12.0 Hz, 1H), 2.84 – 2.06 (m, 14H), 1.97 – 1.57 (m, 13H), 1.55 – 1.41 (m, 2H), 1.36 – 1.16 (m, 3H), 1.12 – 0.90 (m, 5.6H), 0.77 – 0.73 (m, 0.4H), 0.71 – 0.61 (m, 2.5H) 0.34 – 0.27 (m, 0.5H). HRMS (ESI) for $C_{50}H_{64}N_{15}O_4S$ $[M+H]^+$ calculated 970.4986, found 970.4991.

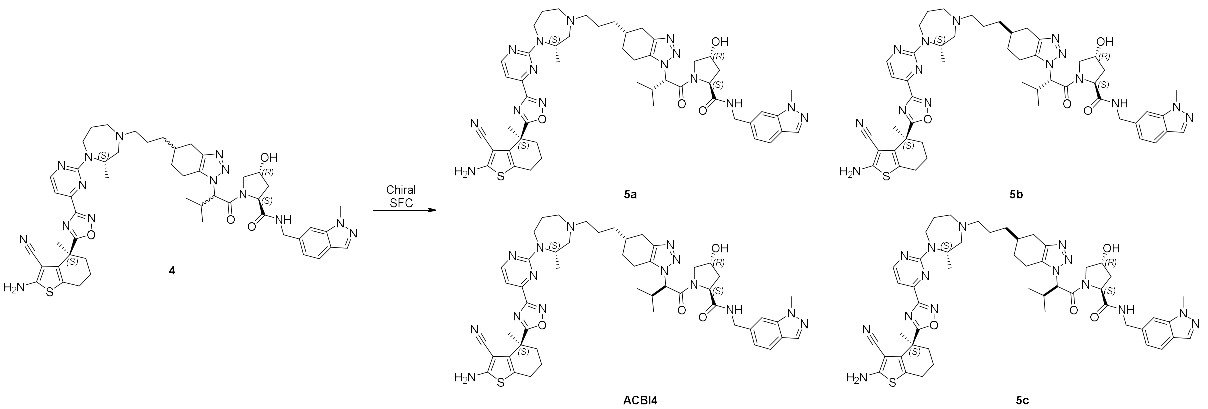

4 (50 mg) is separated by chiral SFC to give **5a** (9.1 mg), **5b** (9.2 mg), **5c** (3.1 mg) and **ACBI4** (3.0 mg) as off-white solids. Stereochemistry was arbitrarily assigned for undefined stereocentres in the separated isomers.

5a: ^{1}H NMR (500 MHz, MeOD) δ 8.48 (m, 1H), 7.93 (d, *J* = 1.0 Hz, 1H), 7.73 – 7.60 (m, 2H), 7.28 – 7.14 (m, 1H), 7.05 (dd, *J* = 8.5, 1.0 Hz, 1H), 5.15 (d, *J* = 10.5 Hz, 1H), 4.69 – 4.62 (m, 2H), 4.48 – 4.32 (m, 2H), 4.08 (s, 3H), 3.77 (d, *J* = 11.0 Hz, 1H), 3.24 – 3.08 (m, 2H), 2.98 (d, *J* = 13.0 Hz, 1H), 2.88 – 2.43 (m, 7H), 2.33 (dt, *J* = 16.0, 7.0 Hz, 1H), 2.25 – 2.07 (m, 1H), 2.03 – 1.82 (m, 10H), 1.69 (m, 1H), 1.49 (s, 3H), 1.25 – 1.05 (m, 8H), 0.96 – 0.82 (m, 0.5H), 0.75 (d, *J* = 6.5 Hz, 0.25H), 0.70 (d, *J* = 6.5 Hz, 2.5H), 0.28 (d, *J* = 6.5 Hz, 0.25H).

5b: ^{1}H NMR (500 MHz, MeOD) δ 8.48 (d, *J* = 5.0 Hz, 1H), 8.00 – 7.89 (m, 1H), 7.79 – 7.52 (m, 2H), 7.19 (d, *J* = 5.0 Hz, 1H), 7.05 (dd, *J* = 8.5, 1.5 Hz, 1H), 5.14 (d, *J* = 10.5 Hz, 1H), 5.03 – 4.93 (m, 0.5H), 4.70 – 4.62 (m, 1H), 4.61 – 4.29 (m, 4H), 4.23 – 3.95 (m, 3H), 3.76 (d, *J* = 11.0 Hz, 1H), 3.67 – 3.50 (m, 1H), 3.25 – 3.06 (m, 2H), 3.04 – 2.92 (m, 1H), 2.88 – 2.40 (m, 11H), 2.37 – 2.09 (m, 4H), 2.06 – 1.81 (m, 9H), 1.76 – 1.38 (m, 5H), 1.29 (s, 1H), 1.24 – 1.06 (m, 9H), 0.94 – 0.56 (m, 3.75H), 0.29 (d, *J* = 7.0 Hz, 0.25H).

5c: ^{1}H NMR (500 MHz, MeOD) δ 8.49 (d, *J* = 5.0 Hz, 1H), 7.95 (d, *J* = 1.0 Hz, 1H), 7.74 – 7.61 (m, 2H), 7.20 (d, *J* = 5.0 Hz, 1H), 7.10 (dd, *J* = 8.0, 1.5 Hz, 1H), 5.12 (d, *J* = 11.0 Hz, 1H), 5.05 (br s, 0.5H), 4.70 (d, *J* = 15.5 Hz, 1H), 4.63 – 4.35 (m, 3H), 4.10 (s, 3H), 3.89 (dd, *J* = 11.0, 4.0 Hz, 1H), 3.72 (d, *J* = 11.0 Hz, 1H), 3.23 (br s, 2H), 3.11 (br d, *J* = 16.5 Hz, 1H), 3.03 (br d, *J* = 13.0 Hz, 1H), 2.91 – 2.65 (m, 4.5H), 2.64 – 2.47 (m, 5H), 2.30 – 2.17 (m, 3H), 2.08 (ddd, *J* = 13.0, 9.0, 4.5 Hz, 1H), 2.03 – 1.84 (m, 9H), 1.81 – 1.57 (m, 5H), 1.47 – 1.29 (m, 4H), 1.16 – 1.12 (d, *J* = 6.5 Hz, 3H), 1.09 (d, *J* = 6.5 Hz, 3H), 0.76 (d, *J* = 6.5 Hz, 3H).

ACBI4: ^{1}H NMR (500 MHz, MeOD) δ 8.49 (br s, 1H), 7.95 (s, 1H), 7.71 – 7.66 (m, 2H), 7.21 (d, *J* = 5.0 Hz, 1H), 7.10 (d, *J* = 9.0 Hz, 1H), 5.10 (d, *J* = 10.5 Hz, 1H), 4.69 (d, *J* = 15.5 Hz, 1H), 4.55 (t, *J* = 8.0 Hz, 1H), 4.49 – 4.43 (m, 2H), 4.10 (s, 3H), 3.86 (dd, *J* = 10.9, 4.1 Hz, 1H), 3.59 – 3.51 (m, 1H), 3.26 (br s, 2H), 3.05 (br d, *J* = 13.0 Hz, 1H), 2.94 – 2.68 (m, 7H), 2.60 (m, 5H), 2.35 – 2.16 (m, 3H), 2.07 (ddd, *J* = 13.0, 8.5, 4.5 Hz, 1H), 2.02 – 1.83 (m, 10H), 1.81 – 1.60 (m, 4H), 1.47 (br s, 1H), 1.32 (m, 3H), 1.15 – 1.11 (d, *J* = 6.5 Hz, 3H), 1.08 (d, *J* = 6.5 Hz, 3H), 0.75 (d, *J* = 6.5 Hz, 3H).

NMR Data

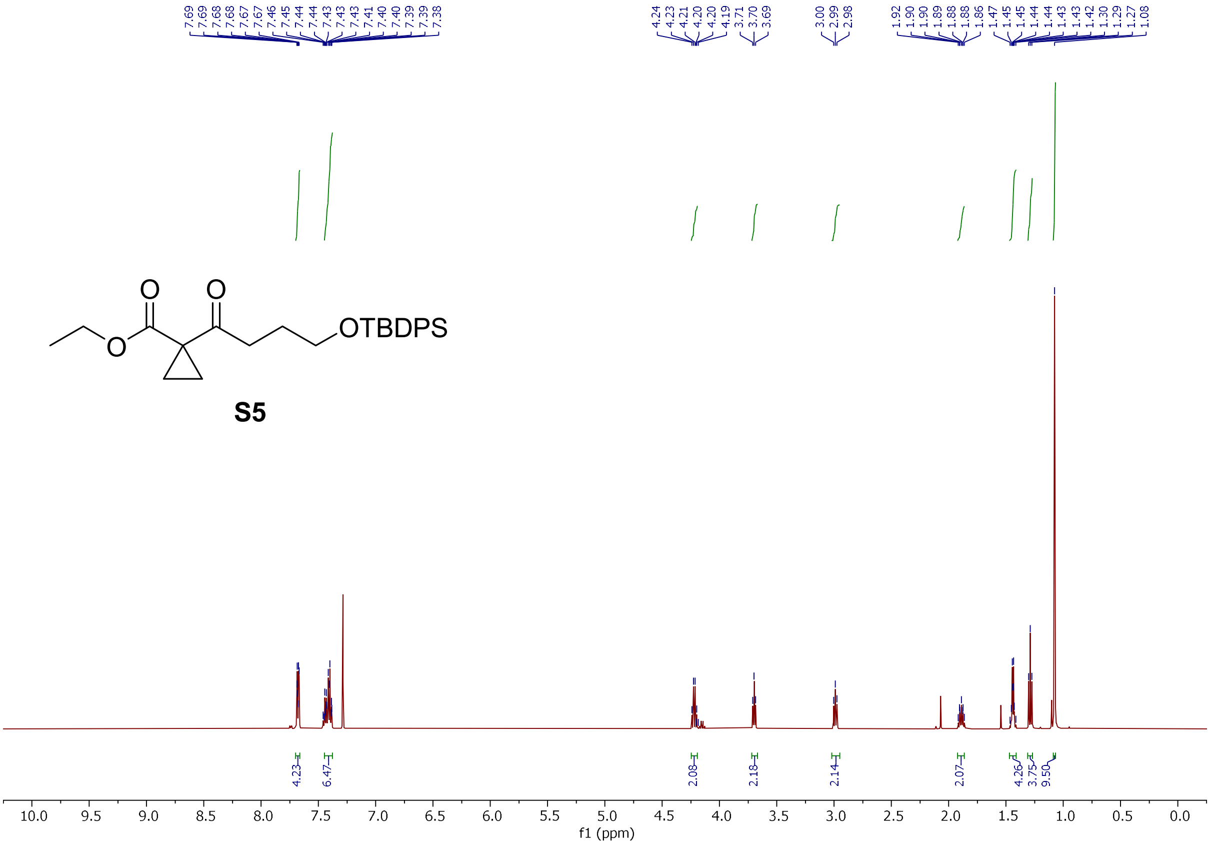

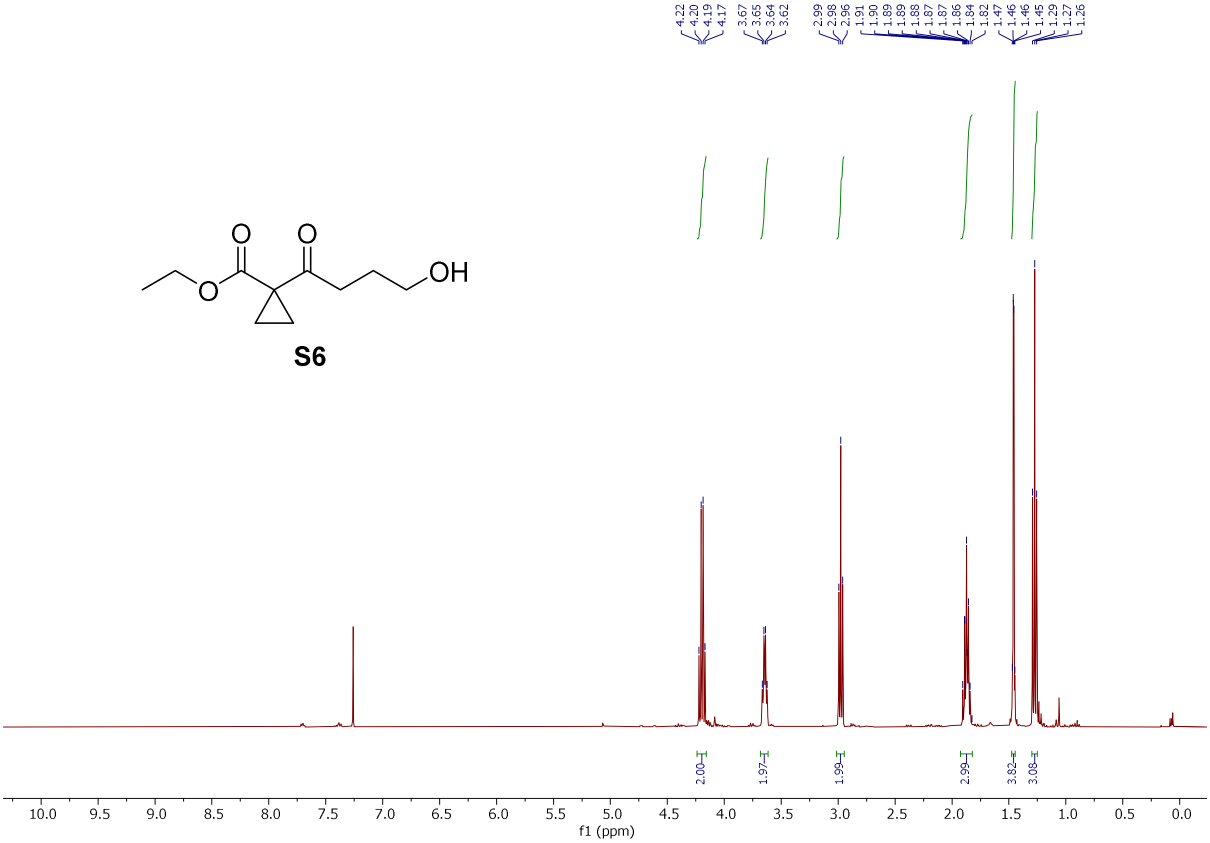

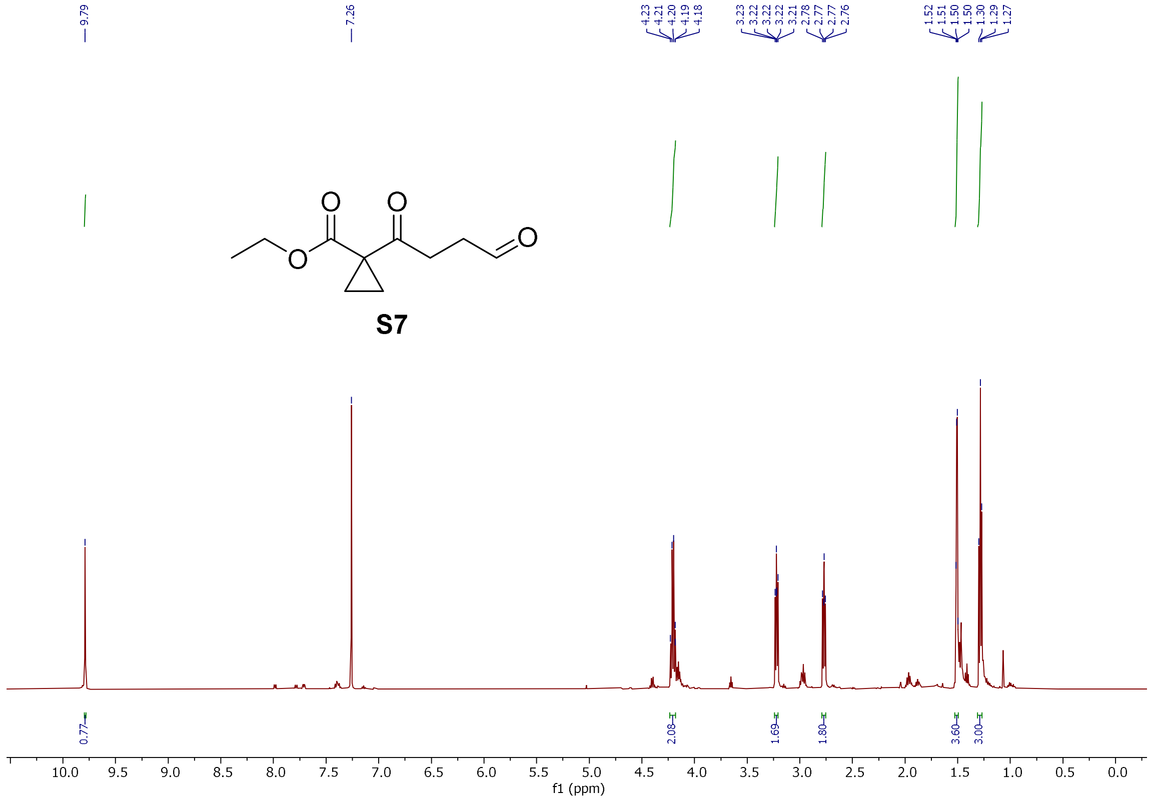

9.79
7.26
4.23 4.21 4.20 4.19 4.18
3.23 3.22 3.22 3.22 3.21 2.78 2.77 2.77 2.76
1.52 1.51 1.50 1.50 1.30 1.29 1.27
0.77
2.08
1.69
1.80
3.60
3.00
10.0 9.5 9.0 8.5 8.0 7.5 7.0 6.5 6.0 5.5 5.0 4.5 4.0 3.5 3.0 2.5 2.0 1.5 1.0 0.5 0.0
f1 (ppm)

S7

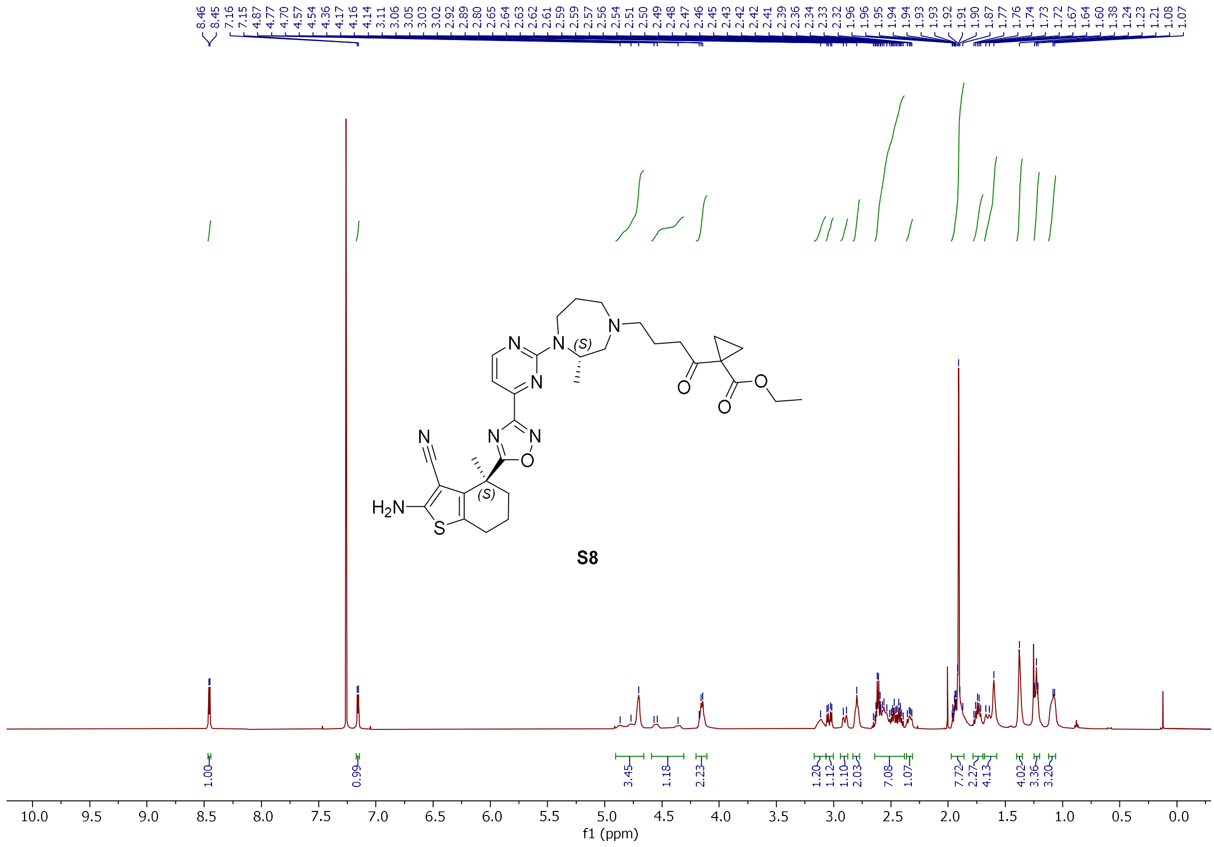

8.46 8.45 7.16 7.15 4.87 4.77 4.70 4.57 4.54 4.36 4.17 4.16 4.14 3.11 3.06 3.05 3.03 3.02 2.92 2.89 2.80 2.65 2.64 2.63 2.62 2.61 2.59 2.59 2.57 2.56 2.54 2.51 2.50 2.49 2.48 2.47 2.46 2.45 2.43 2.42 2.42 2.41 2.39 2.36 2.34 2.33 2.32 1.96 1.95 1.94 1.94 1.93 1.93 1.92 1.91 1.90 1.87 1.77 1.76 1.74 1.73 1.72 1.67 1.64 1.60 1.38 1.24 1.23 1.21 1.08 1.07
N
N
N
(S)
N
N
N
N
O
(S)
H2N
S
O
O
O
1.00
0.99
3.45
1.18
2.23
1.20
1.12
1.10
2.03
7.08
1.07
7.72
2.27
4.13
4.02
3.36
3.20
10.0 9.5 9.0 8.5 8.0 7.5 7.0 6.5 6.0 5.5 5.0 4.5 4.0 3.5 3.0 2.5 2.0 1.5 1.0 0.5 0.0
f1 (ppm)

S8

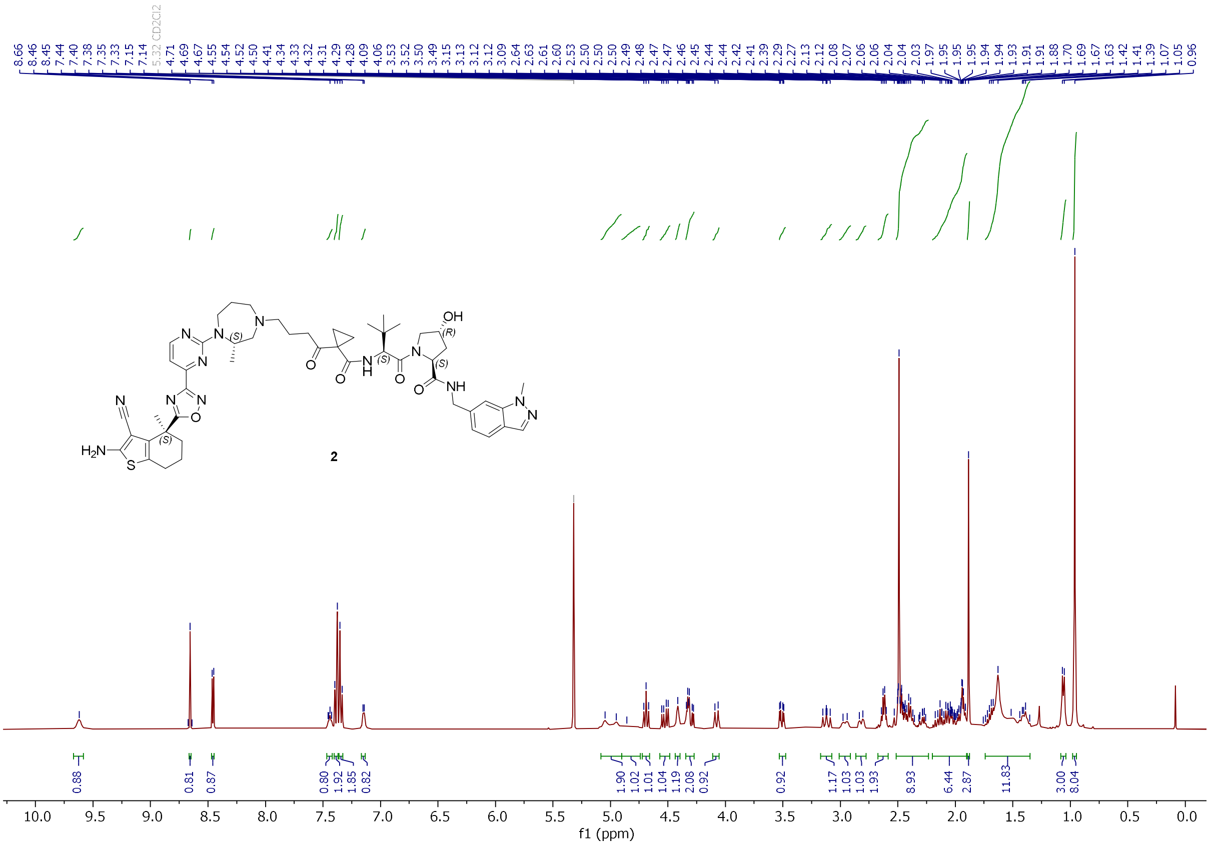

2

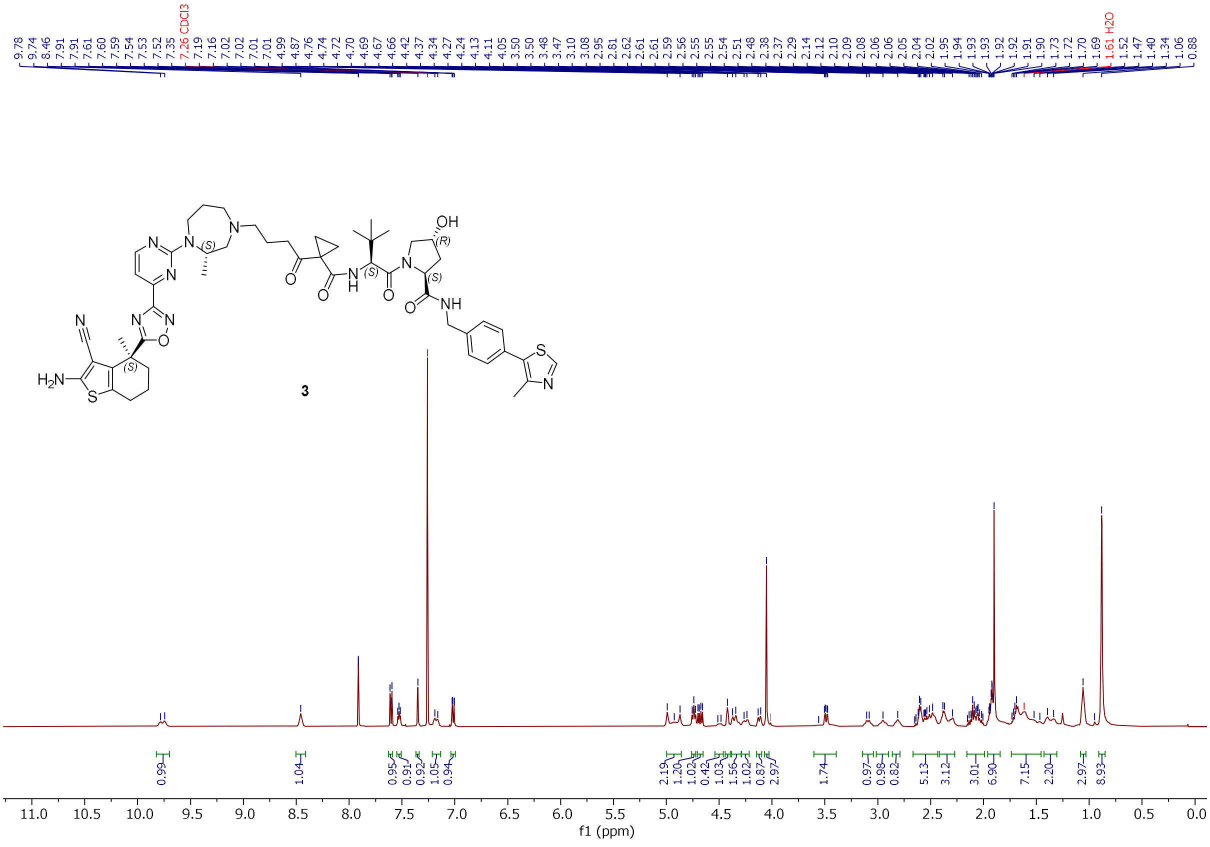

3

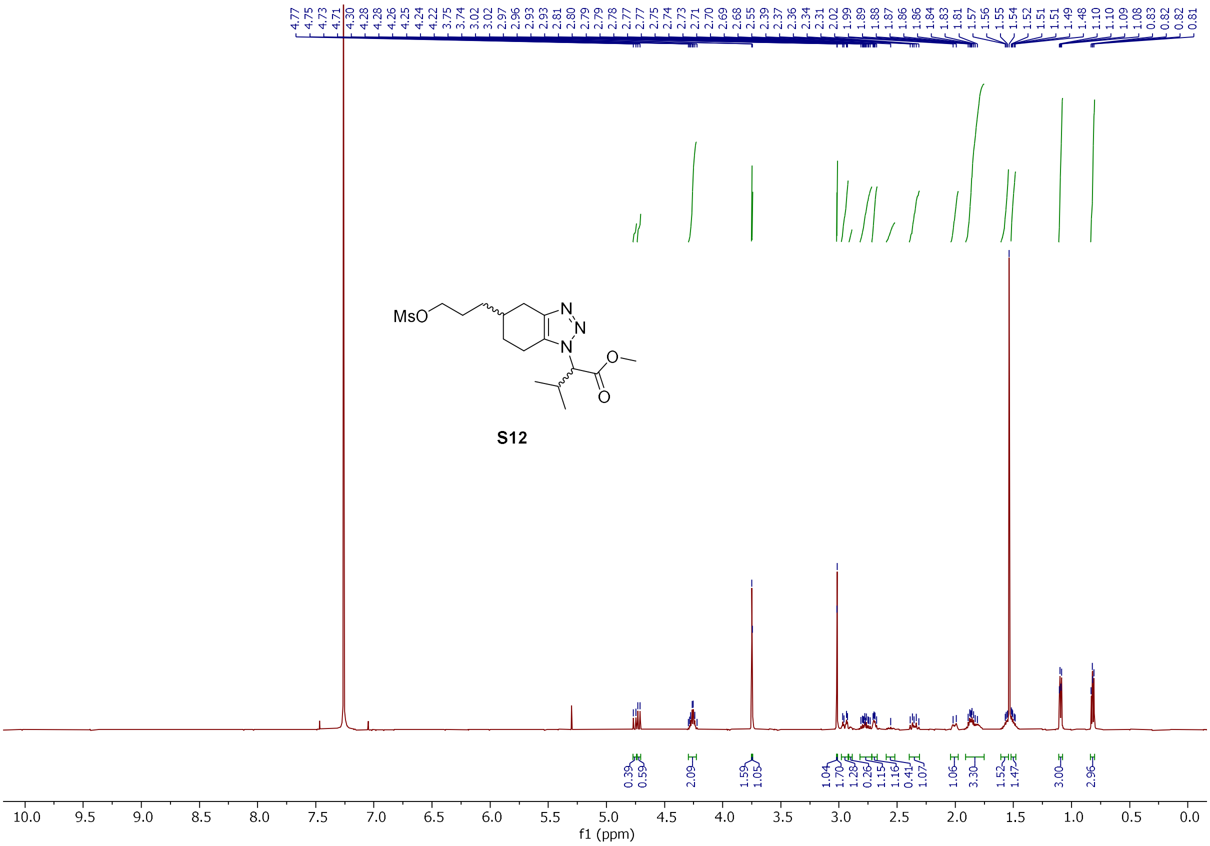
MsO
S12
f1 (ppm)

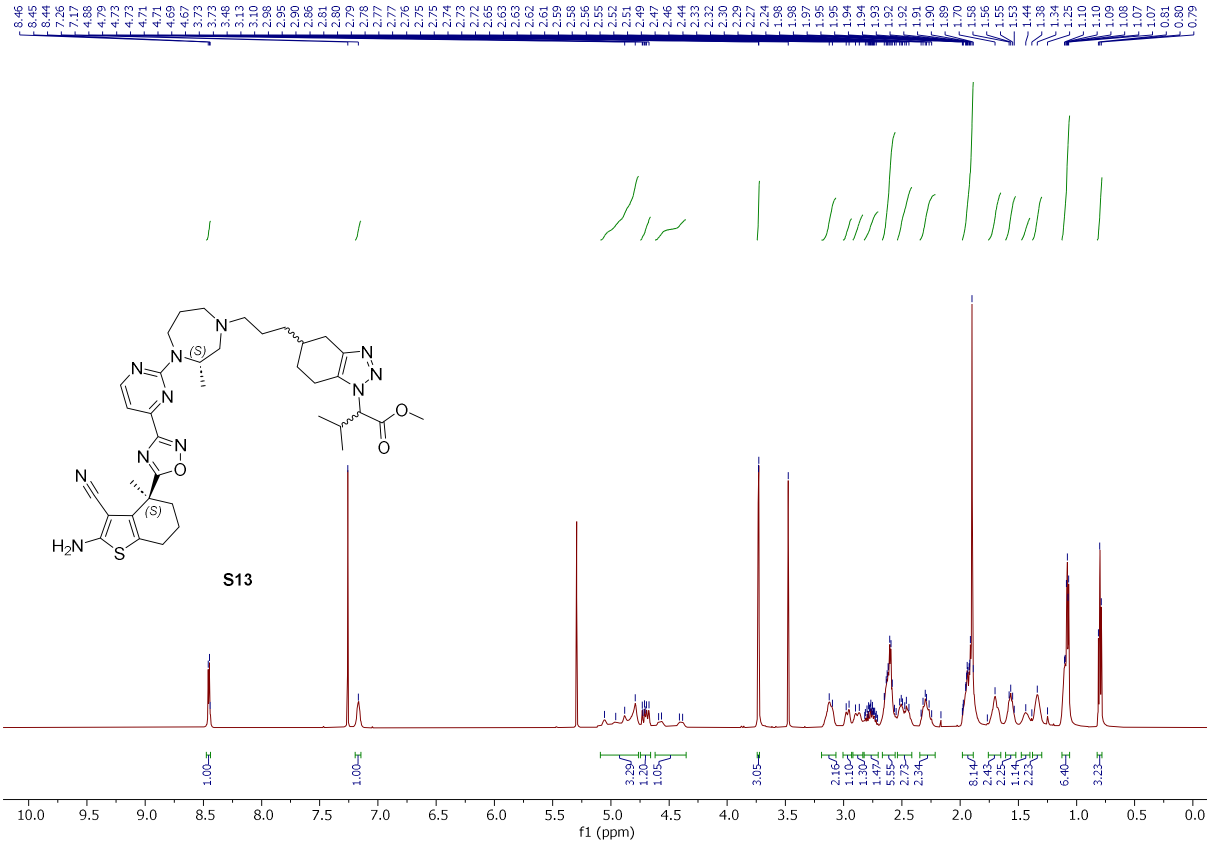
S13
f1 (ppm)

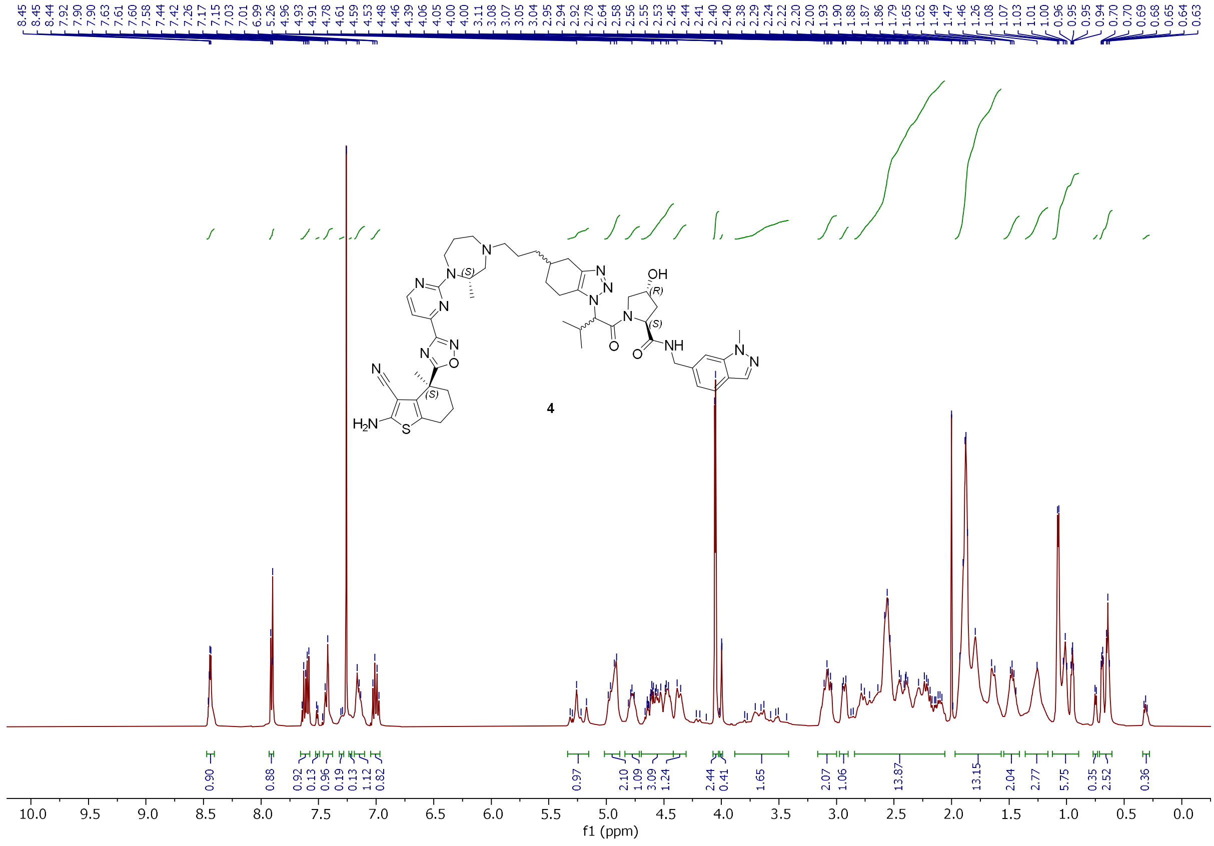

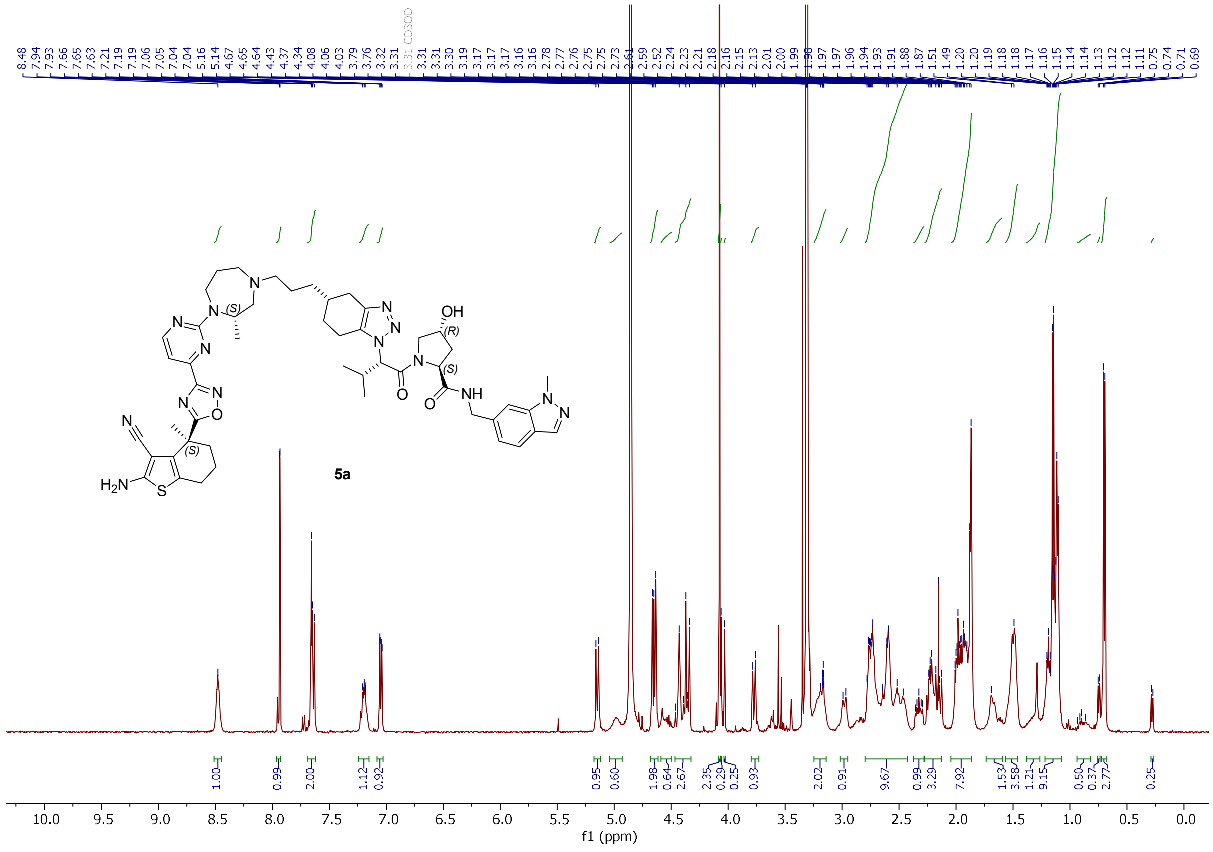

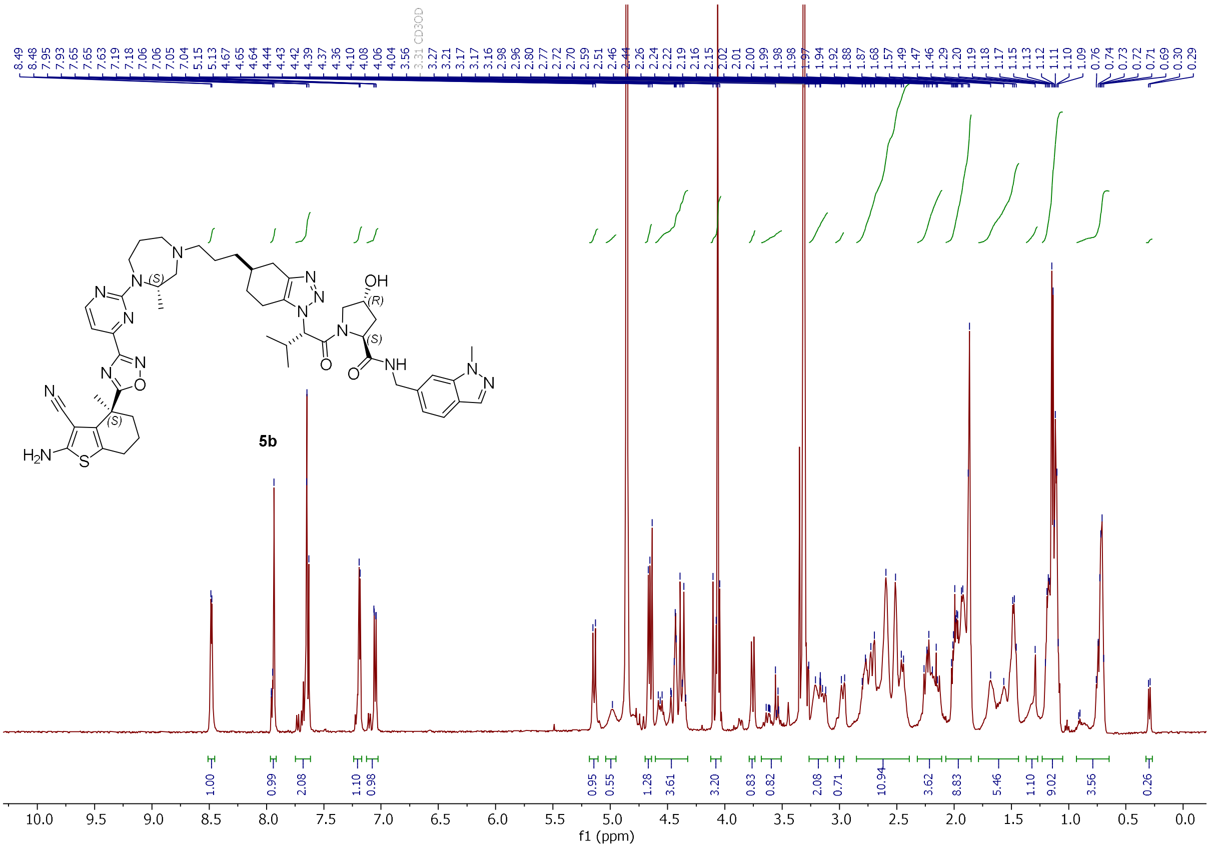

5b
f1 (ppm)

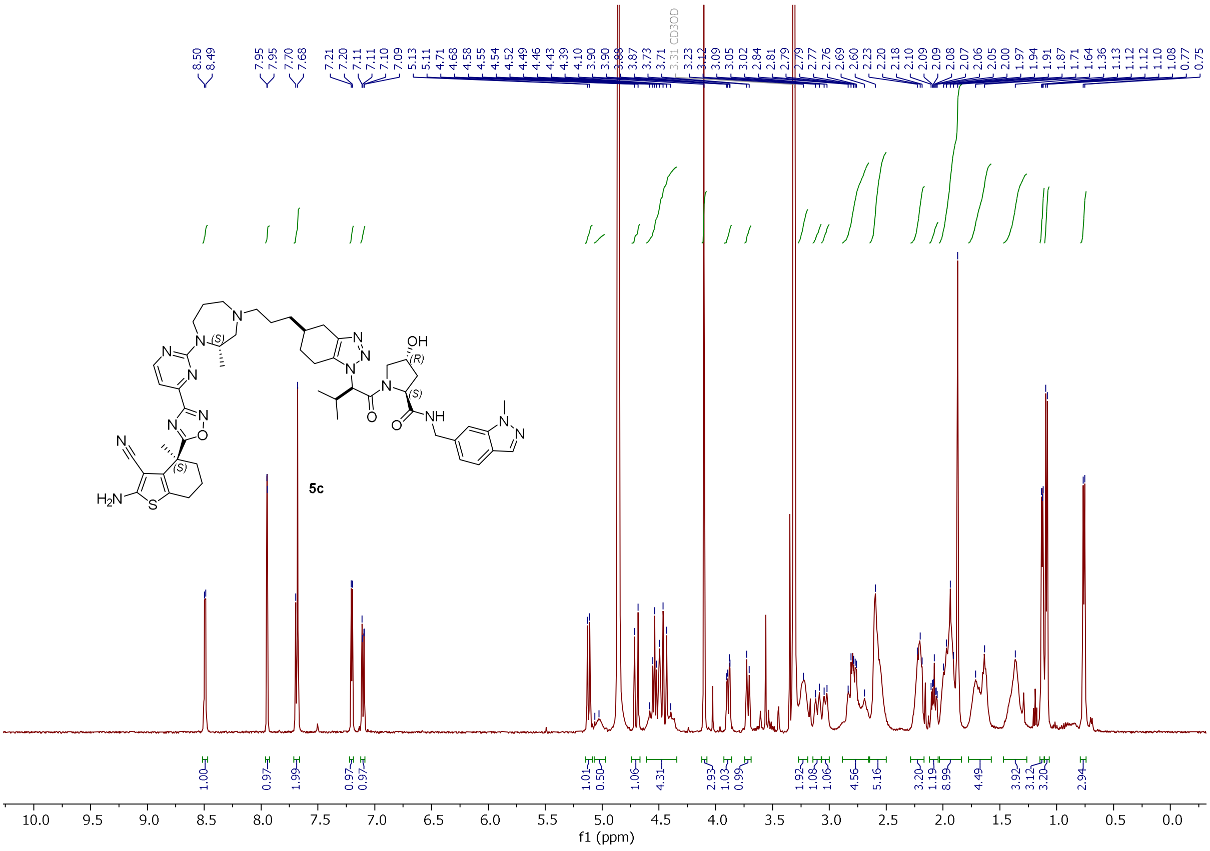

5c
f1 (ppm)

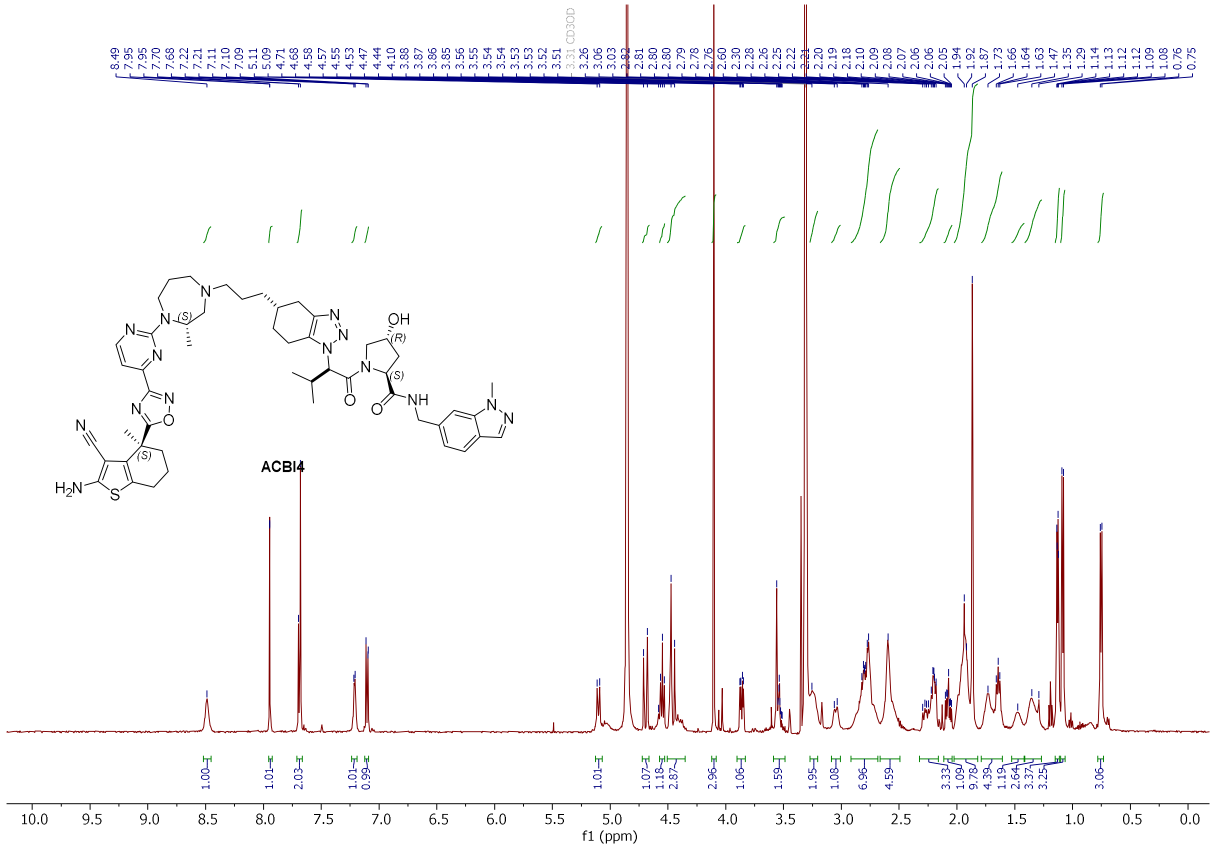
ACBI4
f1 (ppm)
CD3OD

Supplementary Data and Figures

Table S1a: SPR $KRAS^{G12D}$-GCP Characterisation Data Summary

		1	ACBI3	2	3	4	ACBI4	5c
KRAS immobilised - binary	$KRAS^{G12D}$-GCP K_D (nM), (number of repeats)	1466 +/- 193 (5)	719 +/- 147 (5)	246 +/- 50 (5)	161 +/- 22 (5)	148+/- 20 (5)	125 +/- 16 (5)	194 +/- 40 (5)
	$KRAS^{G12D}$-GCP $t_{1/2}$ (s)	15 +/- 2	13 +/- 3	21 +/- 2	46 +/- 3	24 +/- 2	32 +/- 4	20 +/- 3
	$KRAS^{G12D}$-GCP k_a [1/ (M s) *10^5]	0.32 +/- 0.05	0.76 +/- 0.08	1.41 +/- 0.36	0.94 +/- 0.13	2.04 +/- 0.35	1.78 +/- 0.09	1.92 +/- 0.48
	$KRAS^{G12D}$-GCP k_d (1/s)	0.0461 +/- 0.0067	0.0549 +/- 0.0142	0.0329 +/- 0.0036	0.015 +/- 0.0011	0.0296 +/- 0.0028	0.0221 +/- 0.0027	0.036 +/- 0.0058
	$KRAS^{G12D}$-GCP Rmax (RU)	45 +/- 2	39 +/- 2	33 +/- 1	38 +/- 1	36 +/- 1	34 +/- 1	36 +/- 1
	$KRAS^{G12D}$-GCP binding capacity (%)	112.3 ± 4.8	95.4 ± 5.6	92.6 ± 2.4	83.2 ± 3.6	89.5 ± 2.3	86.9 ± 2.4	91.1 ± 3.4
	$KRAS^{G12D}$-GCP Chi^2 (RU^2)	1.49 +/- 1.6	1.41 +/- 0.7	1.1 +/- 0.64	0.93 +/- 0.61	1.27 +/- 0.63	1.76 +/- 0.74	3.21 +/- 2.01
	$KRAS^{G12D}$-GCP U-value	6 +/- 2.3	8 +/- 1.6	6 +/- 1.9	3 +/- 1.2	5 +/- 1.5	6 +/- 1.9	9 +/- 1.8

KRAS immobilised - ternary	$KRAS^{G12D}$-GCP+VCB K_D (nM), (number of repeats)	221 +/- 12 (5)	987 +/- 241 (4)	38.8 +/- 9 (5)	38.4 +/- 2.3 (5)	2.4 +/- 0.2 (4)	0.9 +/- 0.2 (4)	3.4 +/- 0.8 (5)
	$KRAS^{G12D}$-GCP+VCB $t_{1/2}$ (s)	42 +/- 1	9 +/- 2	56 +/- 4	93 +/- 6	641 +/- 72	3283 +/- 155	837 +/- 54
	$KRAS^{G12D}$-GCP+VCB k_a [1/ (M s) *10^5]	0.74 +/- 0.04	0.8 +/- 0.07	3.43 +/- 1.03	1.96 +/- 0.16	4.48 +/- 0.34	2.41 +/- 0.7	2.64 +/- 0.75
	$KRAS^{G12D}$-GCP+VCB k_d (1/s)	0.0164 +/- 0.0003	0.0778 +/- 0.013	0.0124 +/- 0.0009	0.0075 +/- 0.0005	0.0011 +/- 0.0001	0.0002 +/- 0.00001	0.0008 +/- 0.0001
	$KRAS^{G12D}$-GCP+VCB Rmax (RU)	21 +/- 1	18 +/- 1	24 +/- 1	24 +/- 1	20 +/- 2	21 +/- 2	21 +/- 2
	$KRAS^{G12D}$-GCP+VCB binding capacity (%)	32 ± 1.9	27.8 ± 1.4	37.6 ± 2.5	37.4 ± 2.6	30.2 ± 2.1	32.4 ± 1.4	31.8 ± 3.7
	$KRAS^{G12D}$-GCP+VCB Chi^2 (RU^2)	0.1 +/- 0.05	0.44 +/- 0.36	0.75 +/- 0.7	0.48 +/- 0.4	0.22 +/- 0.1	0.08 +/- 0.02	0.2 +/- 0.16
	$KRAS^{G12D}$-GCP+VCB U-value	2 +/- 0.6	10 +/- 2.1	4 +/- 1.5	3 +/- 1.3	2 +/- 0.4	1 +/- 0	2 +/- 0.4
	Co-operativity (α)	6.6 +/- 0.6	0.8 +/- 0.1	6.7 +/- 2.2	4.2 +/- 0.5	59.1 +/- 10.7	142.6 +/- 25	60.4 +/- 14.5

Values are derived from single cycle experiment kinetic fitting. Binding affinities towards $KRAS^{G12D}$, as well as $KRAS^{G12D}$ ± VCB ternary complex affinity and half-lives. Errors are ± standard deviation with repeats (N) specified in brackets. Binding capacity is calculated from the observed to calculated Rmax ratio. Ternary complex cooperativity (alpha) is calculated as the ratio between $KRAS^{G12D}$ K_D / $KRAS^{G12D}$ + VCB K_D values measured in the same run.

Table S1b: SPR KRAS^{G12D}-GDP Characterisation Data Summary

		1	ACBI3	2	3	4	ACBI4	5c
KRAS immobilised - binary	KRAS^{G12D}-GDP K_D (nM), (number of repeats)	10.39 +/- 3.2 (4)	6.72 +/- 0.8 (3)	2.87 +/- 0.4 (3)	2.43 +/- 0.8 (4)	1.24 +/- 0.4 (4)	0.96 +/- 0.1 (3)	1.78 +/- 0.7 (4)
	KRAS^{G12D}-GDP $t_{1/2}$ (s)	231 +/- 41	156 +/- 32	387 +/- 29	631 +/- 81	302 +/- 66	330 +/- 36	286 +/- 48
	KRAS^{G12D}-GDP k_a [1/ (M s) *10^5]	3.4 +/- 1.38	6.86 +/- 0.87	6.32 +/- 0.35	4.92 +/- 1.13	21.9 +/- 8.3	22.7 +/- 4.66	16.45 +/- 6.55
	KRAS^{G12D}-GDP k_d (1/s)	0.0031 +/- 0.0007	0.0046 +/- 0.0009	0.0018 +/- 0.0001	0.0011 +/- 0.0002	0.0024 +/- 0.0004	0.0021 +/- 0.0002	0.0025 +/- 0.0004
	KRAS^{G12D}-GDP Rmax (RU)	43 +/- 1	41 +/- 1	31 +/- 1	38 +/- 1	36 +/- 1	36 +/- 1	37 +/- 1
	KRAS^{G12D}-GDP binding capacity (%)	104.3 ± 3.8	99.4 ± 1.6	91.7 ± 2.3	78.2 ± 2.0	88.7 ± 3.2	90.3 ± 1.7	89.2 ± 4.1
	KRAS^{G12D}-GDP Chi^2 (RU^2)	0.23 +/- 0.15	0.24 +/- 0.12	0.46 +/- 0.11	0.28 +/- 0.28	1.04 +/- 1.04	0.39 +/- 0.15	2.5 +/- 3.68
	KRAS^{G12D}-GDP U-value	2 +/- 0.9	2 +/- 0.5	2 +/- 0	1 +/- 0.4	3 +/- 1.5	2 +/- 0.8	3 +/- 3.3

KRAS immobilised - ternary	KRAS^{G12D}-GDP+VCB K_D (nM), (number of repeats)	7.8 +/- 0.81 (4)	9.4 +/- 1.63 (3)	6.06 +/- 0.91 (4)	7.04 +/- 0.65 (4)	0.23 +/- 0.06 (3)	0.08 +/- 0.05 (4)	0.25 +/- 0.03 (4)
	KRAS^{G12D}-GDP+VCB $t_{1/2}$ (s)	225 +/- 13	196 +/- 34	66 +/- 6	73 +/- 10	274 +/- 124	11699 +/- 6899	3232 +/- 385
	KRAS^{G12D}-GDP+VCB k_a [1/ (M s) *10^5]	4.03 +/- 0.62	3.88 +/- 0.26	17.73 +/- 2.38	13.96 +/- 3.06	146.67 +/- 73.86	12.46 +/- 3.96	8.84 +/- 0.59
	KRAS^{G12D}-GDP+VCB k_d (1/s)	0.0031 +/- 0.0002	0.0036 +/- 0.0006	0.0106 +/- 0.001	0.0097 +/- 0.0014	0.003 +/- 0.001	0.0001 +/- 0.00005	0.0002 +/- 0.00003
	KRAS^{G12D}-GDP+VCB Rmax (RU)	23 +/- 1	21 +/- 0	26 +/- 1	27 +/- 1	23 +/- 2	21 +/- 3	19 +/- 2
	KRAS^{G12D}-GDP+VCB binding capacity (%)	35.0 ± 8.7	34.8 ± 6.7	40.9 ± 7.6	40.4 ± 7.4	33.6 ± 7.2	32.2 ± 2.8	28.9 ± 6.3
	KRAS^{G12D}-GDP+VCB Chi^2 (RU^2)	0.15 +/- 0.1	2.04 +/- 1.05	2.21 +/- 1.17	0.85 +/- 0.55	0.55 +/- 0.11	0.19 +/- 0.16	0.11 +/- 0.02
	KRAS^{G12D}-GDP+VCB U-value	1 +/- 0.4	8 +/- 1.9	7 +/- 3.2	6 +/- 3.9	10 +/- 5.6	2 +/- 0.5	2 +/- 0.5
	Co-operativity (α)	1.2 +/- 0.3	0.9 +/- 0	0.5 +/- 0.1	0.3 +/- 0.1	6.3 +/- 3.8	19.8 +/- 13.4	7.5 +/- 3.4

Values are derived from single cycle experiment kinetic fitting. Binding affinities towards KRAS^{G12D}, as well as KRAS^{G12D} ± VCB ternary complex affinity and half-lives. Errors are ± standard deviation with repeats (N) specified in brackets. Binding capacity is calculated from the observed to calculated Rmax ratio. Ternary complex cooperativity (alpha) is calculated as ratio the between KRAS^{G12D} K_D / KRAS^{G12D} + VCB K_D values measured in the same run.

Table S1c: SPR KRAS^{G12R}-GCP Characterisation Data Summary

		1	ACBI3	2	3	4	5a	5b	ACBI4	5c
binary	KRAS^{G12R}-GCP K_D (nM), (number of repeats)	1396.7 +/- 185.5 (3)	251.8 +/- 12.8 (4)	166.8 +/- 23.4 (4)	169.3 +/- 10.2 (3)	111.4 +/- 27 (4)	105.2 +/- 16.4 (4)	250.5 +/- 59.8 (4)	140.9 +/- 32.2 (4)	178.5 +/- 24.4 (4)
	KRAS^{G12R}-GCP $t_{1/2}$ (s)	14 +/- 1	14.6 +/- 0.8	35.9 +/- 8.8	20.9 +/- 0.1	22.1 +/- 4.9	19.2 +/- 4	12.7 +/- 1	19.9 +/- 2.7	18.4 +/- 5.2
	KRAS^{G12R}-GCP k_a [1/(M s)*10^5]	0.37 +/- 0.07	1.91 +/- 0.18	1.32 +/- 0.59	1.97 +/- 0.13	3.28 +/- 1.45	3.78 +/- 1.36	2.29 +/- 0.44	2.69 +/- 0.75	2.41 +/- 0.99
	KRAS^{G12R}-GCP k_d (1/s)	0.05 +/- 0.004	0.048 +/- 0.002	0.021 +/- 0.006	0.033 +/- 0	0.033 +/- 0.007	0.038 +/- 0.009	0.055 +/- 0.005	0.036 +/- 0.006	0.042 +/- 0.015
	KRAS^{G12R}-GCP Rmax (RU)	37.1 +/- 2.4	37.4 +/- 2.4	36.6 +/- 2.3	33.3 +/- 2.3	36.9 +/- 2.5	34.1 +/- 2.3	36.5 +/- 4	36.1 +/- 1.7	38.2 +/- 3.1
	KRAS^{G12R}-GCP Binding capacity (%)	97.5 +/- 3.1	89.7 +/- 0.3	89.2 +/- 2.6	86.2 +/- 2.6	86.3 +/- 3.1	84.8 +/- 5.7	88.4 +/- 3.3	87 +/- 2	90 +/- 3.7
	KRAS^{G12R}-GCP Chi^2 (RU^2)	0.44 +/- 0.05	1.2 +/- 0.82	1.11 +/- 0.88	1.03 +/- 0.38	1.32 +/- 1.01	1.12 +/- 0.72	0.66 +/- 0.31	1.29 +/- 0.89	3.33 +/- 3.18
	KRAS^{G12R}-GCP U-value	4 +/- 0.5	5 +/- 0.4	3 +/- 1.1	4 +/- 0.5	5 +/- 1.5	6 +/- 1.7	6 +/- 1.7	6 +/- 1.9	12 +/- 6.3
ternary	KRAS^{G12R}-GCP+VCB K_D (nM), (number of repeats)	201 +/- 30.7 (5)	366 +/- 26.3 (3)	20.6 +/- 3 (4)	23.2 +/- 0.8 (3)	1.8 +/- 0.3 (4)	3.9 +/- 0.4 (4)	8.5 +/- 1.4 (4)	1.6 +/- 0.8 (4)	2.1 +/- 0.6 (4)
	KRAS^{G12R}-GCP+VCB $t_{1/2}$ (s)	34 +/- 3	5.6 +/- 0.9	97.4 +/- 7.3	51.8 +/- 2.7	657.5 +/- 54.2	460.8 +/- 34.7	208.3 +/- 80.1	1862.5 +/- 148.6	710 +/- 32.5

	KRAS^{G12R}-GCP+VCB k_a [1/(M s)*10^5]	1.04 +/- 0.11	3.48 +/- 0.36	3.54 +/- 0.42	5.77 +/- 0.12	6.02 +/- 1.09	3.95 +/- 0.34	5.37 +/- 3.63	2.8 +/- 0.83	4.96 +/- 1.52
	KRAS^{G12R}-GCP+VCB k_d (1/s)	0.0206 +/- 0.0017	0.1277 +/- 0.0208	0.0072 +/- 0.0006	0.0134 +/- 0.0007	0.0011 +/- 0.0001	0.0015 +/- 0.0001	0.0044 +/- 0.0029	0.0004 +/- 0.00003	0.001 +/- 0.00005
	KRAS^{G12R}-GCP+VCB Rmax (RU)	28.8 +/- 3.1	27.1 +/- 3.3	29.8 +/- 3.1	32.7 +/- 2.7	27.9 +/- 2.4	28.2 +/- 2.6	26 +/- 3.6	26 +/- 3.5	28.2 +/- 1.8
	KRAS^{G12R}-GCP+VCB Binding capacity (%)	44.2 +/- 7	43.2 +/- 8.4	45.6 +/- 8.1	50.6 +/- 5.4	42.7 +/- 6.4	43.2 +/- 6.8	38.8 +/- 7.3	38.8 +/- 5.9	42.4 +/- 5.6
	KRAS^{G12R}-GCP+VCB Chi^2 (RU^2)	1.4 +/- 1.2	1.1 +/- 0.8	1.5 +/- 0.6	1.1 +/- 0.3	0.5 +/- 0.2	0.2 +/- 0.1	1.9 +/- 0.9	0.1 +/- 0.04	0.1 +/- 0.1
	KRAS^{G12R}-GCP+VCB U-value	6.2 +/- 3.9	11 +/- 1.4	4 +/- 0.7	4 +/- 0.8	2.3 +/- 0.4	1.8 +/- 0.8	7 +/- 2	1 +/- 0	1.3 +/- 0.4
	Co-operativity (α)	7.4 +/- 2.6	0.7 +/- 0.03	8.3 +/- 1.8	7.3 +/- 0.6	62.2 +/- 15.5	27.5 +/- 4.5	30.3 +/- 9.1	105.9 +/- 44.4	89.3 +/- 24.8

Values are derived from single cycle experiment kinetic fitting. Binding affinities towards KRAS^{G12R}, as well as KRAS^{G12R} ± VCB ternary complex affinity and half-lives. Errors are ± standard deviation with repeats (N) specified in brackets. Binding capacity is calculated from the observed to calculated Rmax ratio. Ternary complex cooperativity (alpha) is calculated as ratio the between KRAS^{G12R} K_D / KRAS^{G12R} + VCB K_D values measured in the same run.

Table S1d: SPR VCB Ternary Characterisation Data Summary

			1	ACBI3	2	3	4	ACBI4	5c
VCB immobilised	+ $KRAS^{G12D}$ GCP	VCB+$KRAS^{G12D-}$GCP K_D (nM), (number of repeats)	111 +/- 24 (5)	17 +/- 0.7 (3)	88.8 +/- 26.2 (5)	51.3 +/- 12.6 (5)	1.2 +/- 0.3 (5)	0.5 +/- 0.1 (3)	1.8 +/- 0.1 (3)
		VCB+$KRAS^{G12D-}$GCP $t_{1/2}$ (s)	56 +/- 2	30 +/- 3	46 +/- 1	81 +/- 4	948 +/- 283	2807 +/- 374	896 +/- 18
		VCB+$KRAS^{G12D-}$GCP k_a [1/ (M s) *10^5]	1.17 +/- 0.23	13.73 +/- 1.5	1.85 +/- 0.55	1.78 +/- 0.45	7.62 +/- 4.27	5.37 +/- 0.36	4.44 +/- 0.34
		VCB+$KRAS^{G12D-}$GCP k_d (1/s)	0.0125 +/- 0.0005	0.0235 +/- 0.0029	0.0151 +/- 0.0004	0.0086 +/- 0.0005	0.0008 +/- 0.0003	0.0003 +/- 0.00004	0.0008 +/- 0.00002
		VCB+$KRAS^{G12D}$-GCP Rmax (RU)	32 +/- 13	26 +/- 11	46 +/- 15	44 +/- 15	39 +/- 16	31 +/- 12	42 +/- 11
		$KRAS^{G12D}$-GCP binding capacity (%)	34.2 ± 13.1	28.5 ± 12.5	49.4 ± 14.5	50.2 ± 14.1	42.2 ± 16.6	34.6 ± 9.7	43.2 ± 8.1
		VCB+$KRAS^{G12D}$-GCP Chi^2 (RU^2)	1.34 +/- 0.8	6.4 +/- 5.39	1.15 +/- 1	0.21 +/- 0.13	0.5 +/- 0.39	0.08 +/- 0.07	0.26 +/- 0.1
		VCB+$KRAS^{G12D}$-GCP U-value	4 +/- 1.5	13 +/- 1.4	3 +/- 3.1	1 +/- 0.4	1 +/- 0.4	1 +/- 0	1 +/- 0.5
	+ $KRAS^{G12D}$ GDP	VCB+$KRAS^{G12D}$-GDP K_D (nM), (number of repeats)	27 +/- 3.4 (4)	6.7 +/- 0.3 (3)	42 +/- 2.4 (3)	25.3 +/- 3 (3)	0.68 +/- 0.29 (4)	0.23 +/- 0.11 (3)	0.4 +/- 0.05 (3)
		VCB+$KRAS^{G12D}$-GDP $t_{1/2}$ (s)	197 +/- 8	99 +/- 15	53 +/- 1	95 +/- 3	5122 +/- 2042	8503 +/- 2443	3547 +/- 168
		VCB+$KRAS^{G12D}$-GDP k_a [1/ (M s) *10^5]	1.33 +/- 0.19	10.8 +/- 1.8	3.12 +/- 0.26	2.93 +/- 0.43	2.46 +/- 0.68	4.17 +/- 0.82	4.91 +/- 0.37
		VCB+$KRAS^{G12D}$-GDP k_d (1/s)	0.00352 +/- 0.00014	0.00714 +/- 0.00096	0.01303 +/- 0.00029	0.00729 +/- 0.00025	0.00017 +/- 0.00009	0.00009 +/- 0.00002	0.0002 +/- 0.00001
		VCB+$KRAS^{G12D}$-GDP Rmax (RU)	40 +/- 2.5	45.4 +/- 9.7	56.9 +/- 12.9	56.8 +/- 10.3	44.4 +/- 9.3	37.5 +/- 1.1	42.1 +/- 12.9

		$KRAS^{G12D}$-GDP binding capacity (%)	50.3 ± 4.9	52.4 ± 6.4	64.8 ± 6.7	65.5 ± 8.5	53.6 ± 6.8	47.3 ± 3.7	47.5 ± 10.4
		VCB+$KRAS^{G12D}$-GDP Chi^2 (RU^2)	1.35 +/- 1.34	2.51 +/- 1.06	0.23 +/- 0.1	0.26 +/- 0.13	0.07 +/- 0.02	0.04 +/- 0.03	0.02 +/- 0.01
		VCB+$KRAS^{G12D}$-GDP U-value	3 +/- 1.5	9 +/- 2.4	1 +/- 0.5	1 +/- 0	1 +/- 0	1 +/- 0	1 +/- 0

Values are derived from single cycle experiment kinetic fitting. Binding affinities towards VCB + $KRAS^{G12D}$-GDP, as well as VCB + $KRAS^{G12D}$-GCP ternary complex affinity and half-lives. Binding capacity is calculated from the observed to calculated Rmax ratio. Errors are ± standard deviation with repeats (N) specified in brackets.

Figure S1: SPR characterization of binary and ternary complexes for immobilized KRAS^{G12D}-GCP with VCB

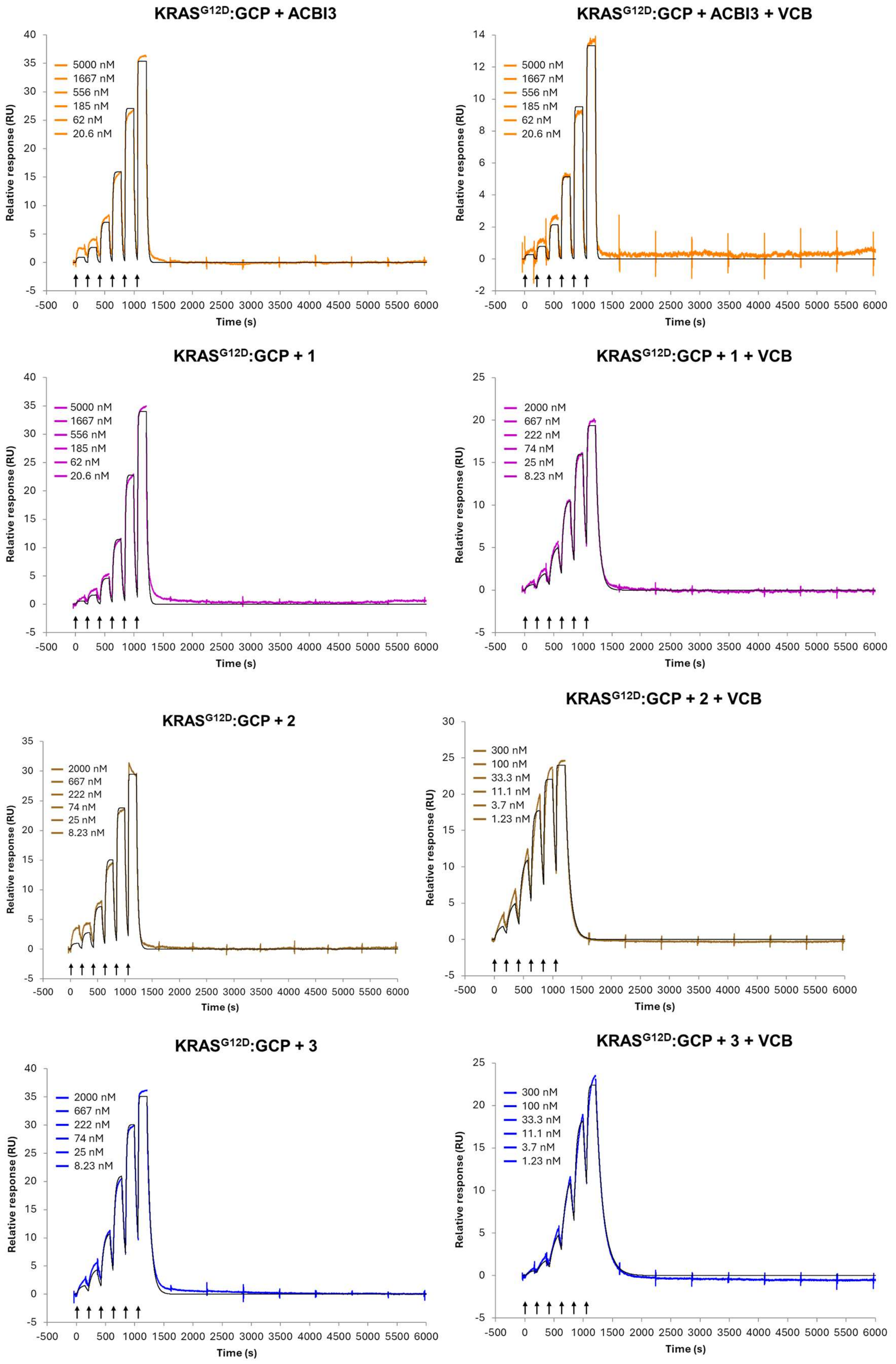

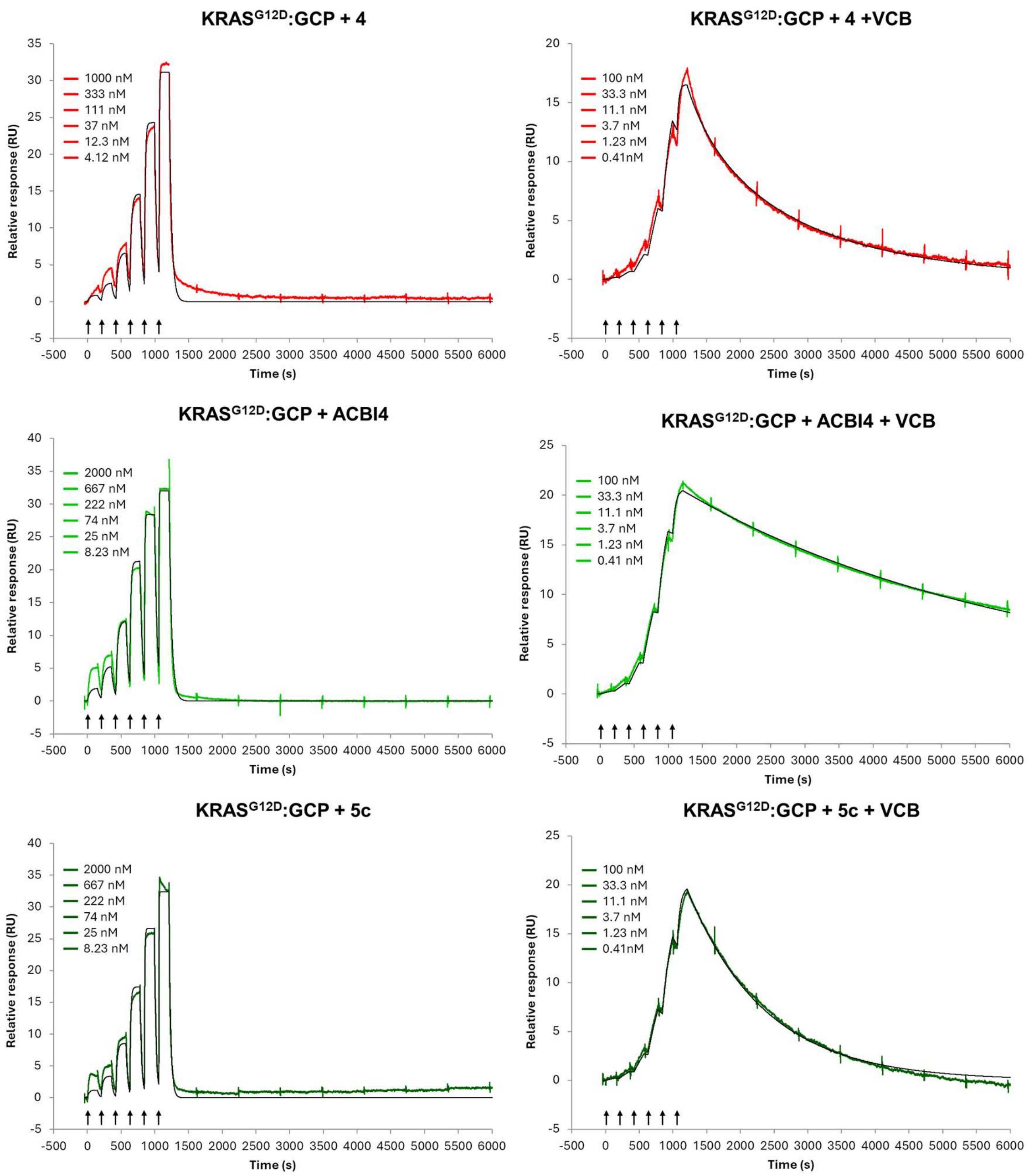
KRASG12D:GCP + 4
1000 nM
333 nM
111 nM
37 nM
12.3 nM
4.12 nM
Relative response (RU)
Time (s)
KRASG12D:GCP + 4 +VCB
100 nM
33.3 nM
11.1 nM
3.7 nM
1.23 nM
0.41nM
Relative response (RU)
Time (s)
KRASG12D:GCP + ACBI4
2000 nM
667 nM
222 nM
74 nM
25 nM
8.23 nM
Relative response (RU)
Time (s)
KRASG12D:GCP + ACBI4 + VCB
100 nM
33.3 nM
11.1 nM
3.7 nM
1.23 nM
0.41 nM
Relative response (RU)
Time (s)
KRASG12D:GCP + 5c
2000 nM
667 nM
222 nM
74 nM
25 nM
8.23 nM
Relative response (RU)
Time (s)
KRASG12D:GCP + 5c + VCB
100 nM
33.3 nM
11.1 nM
3.7 nM
1.23 nM
0.41nM
Relative response (RU)
Time (s)

Figure S2: SPR characterization of binary and ternary complexes for immobilized KRAS^{G12D}-GDP with VCB

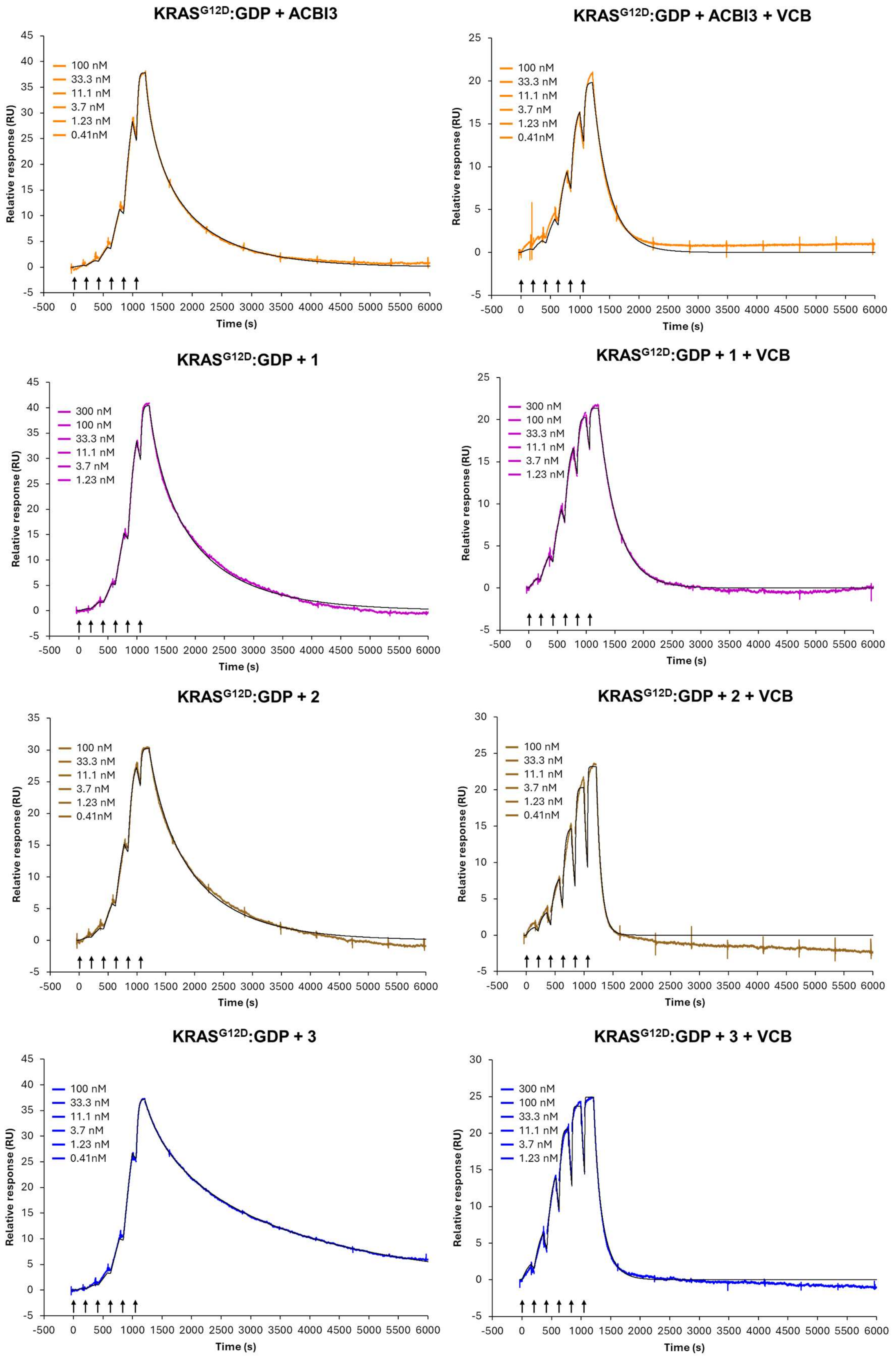

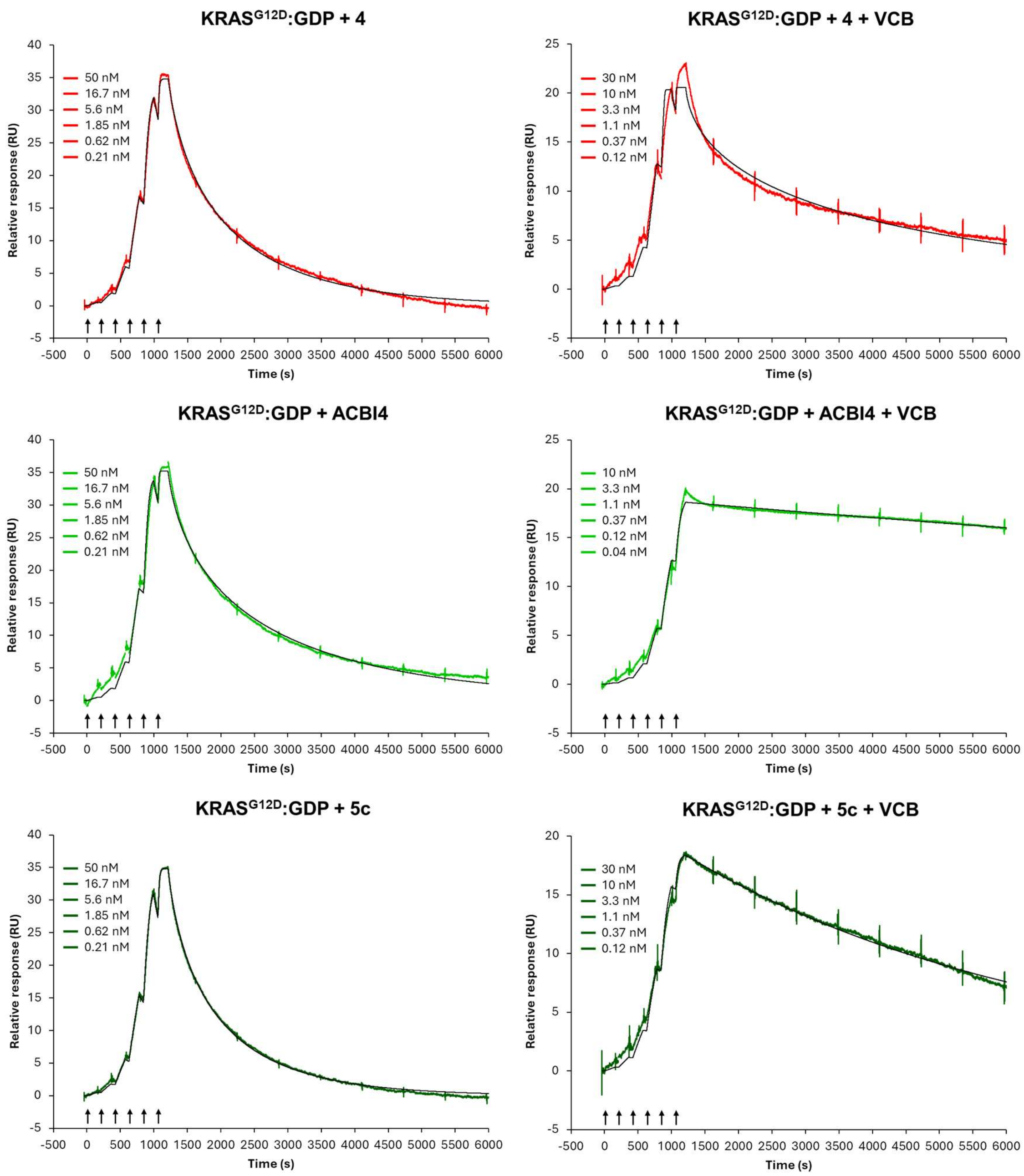
KRAS^{G12D}:GDP + 4
50 nM
16.7 nM
5.6 nM
1.85 nM
0.62 nM
0.21 nM
KRAS^{G12D}:GDP + 4 + VCB
30 nM
10 nM
3.3 nM
1.1 nM
0.37 nM
0.12 nM
KRAS^{G12D}:GDP + ACBI4
50 nM
16.7 nM
5.6 nM
1.85 nM
0.62 nM
0.21 nM
KRAS^{G12D}:GDP + ACBI4 + VCB
10 nM
3.3 nM
1.1 nM
0.37 nM
0.12 nM
0.04 nM
KRAS^{G12D}:GDP + 5c
50 nM
16.7 nM
5.6 nM
1.85 nM
0.62 nM
0.21 nM
KRAS^{G12D}:GDP + 5c + VCB
30 nM
10 nM
3.3 nM
1.1 nM
0.37 nM
0.12 nM
Relative response (RU)
Time (s)

Figure S3: SPR characterization of binary and ternary complexes for immobilized $KRAS^{G12R}$-GCP with VCB

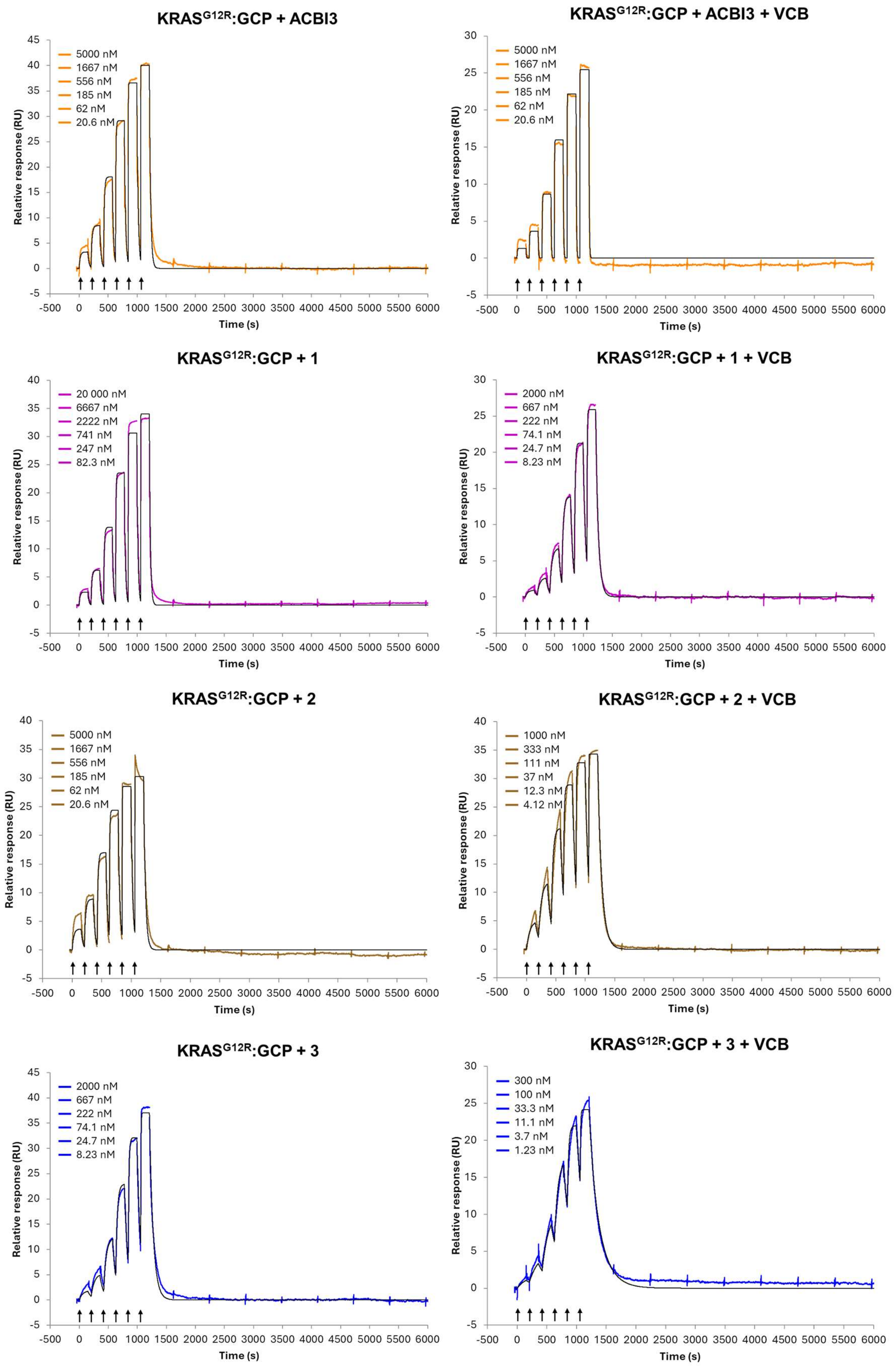

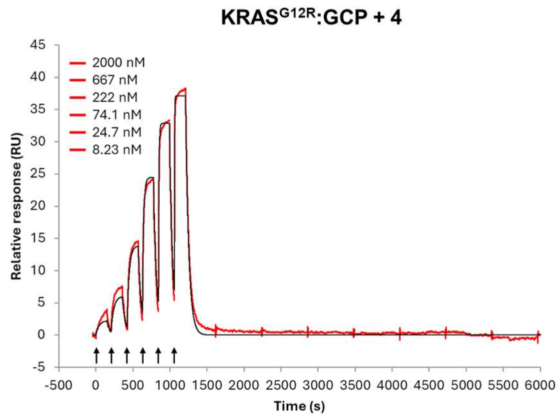
KRAS^{G12R}:GCP + 4
2000 nM
667 nM
222 nM
74.1 nM
24.7 nM
8.23 nM
Relative response (RU)
Time (s)
45 40 35 30 25 20 15 10 5 0 -5
-500 0 500 1000 1500 2000 2500 3000 3500 4000 4500 5000 5500 6000

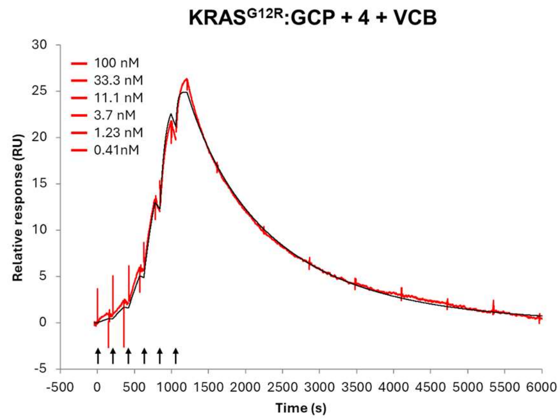
KRAS^{G12R}:GCP + 4 + VCB
100 nM
33.3 nM
11.1 nM
3.7 nM
1.23 nM
0.41nM
Relative response (RU)
Time (s)
30 25 20 15 10 5 0 -5
-500 0 500 1000 1500 2000 2500 3000 3500 4000 4500 5000 5500 6000

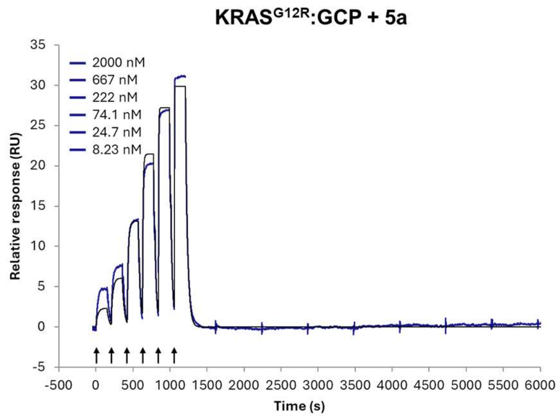
KRAS^{G12R}:GCP + 5a
2000 nM
667 nM
222 nM
74.1 nM
24.7 nM
8.23 nM
Relative response (RU)
Time (s)
35 30 25 20 15 10 5 0 -5
-500 0 500 1000 1500 2000 2500 3000 3500 4000 4500 5000 5500 6000

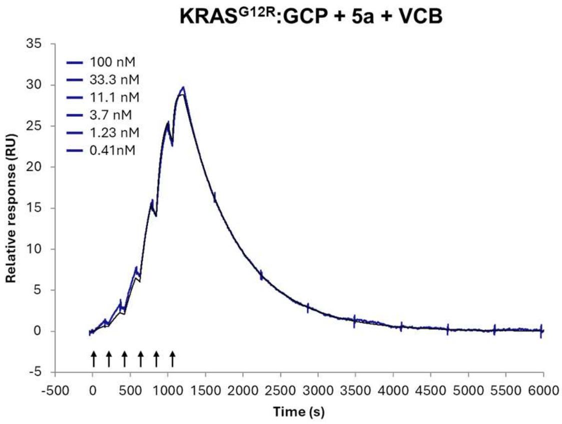
KRAS^{G12R}:GCP + 5a + VCB
100 nM
33.3 nM
11.1 nM
3.7 nM
1.23 nM
0.41nM
Relative response (RU)
Time (s)
35 30 25 20 15 10 5 0 -5
-500 0 500 1000 1500 2000 2500 3000 3500 4000 4500 5000 5500 6000

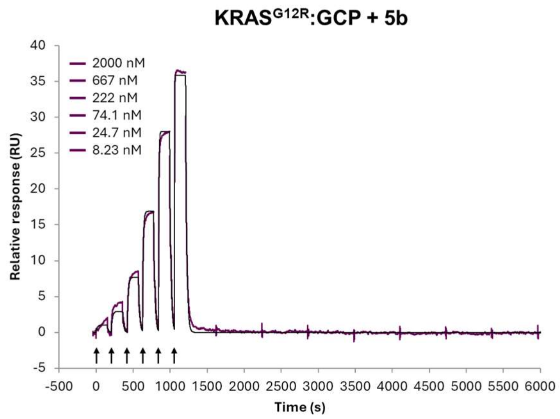
KRAS^{G12R}:GCP + 5b
2000 nM
667 nM
222 nM
74.1 nM
24.7 nM
8.23 nM
Relative response (RU)
Time (s)
40 35 30 25 20 15 10 5 0 -5
-500 0 500 1000 1500 2000 2500 3000 3500 4000 4500 5000 5500 6000

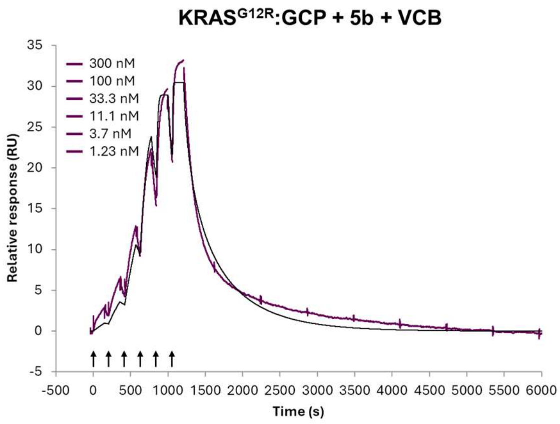
KRAS^{G12R}:GCP + 5b + VCB
300 nM
100 nM
33.3 nM
11.1 nM
3.7 nM
1.23 nM
Relative response (RU)
Time (s)
35 30 25 20 15 10 5 0 -5
-500 0 500 1000 1500 2000 2500 3000 3500 4000 4500 5000 5500 6000

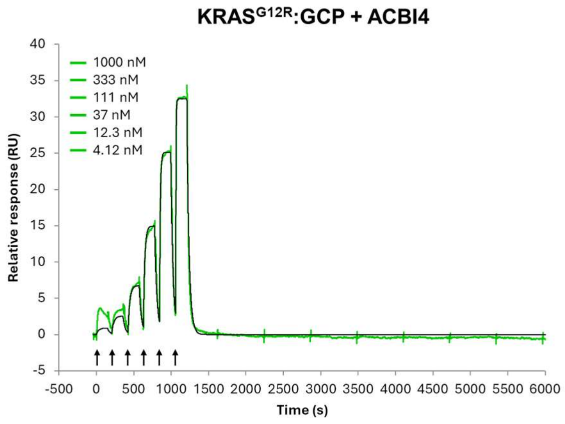
KRAS^{G12R}:GCP + ACBI4
1000 nM
333 nM
111 nM
37 nM
12.3 nM
4.12 nM
Relative response (RU)
Time (s)
40 35 30 25 20 15 10 5 0 -5
-500 0 500 1000 1500 2000 2500 3000 3500 4000 4500 5000 5500 6000

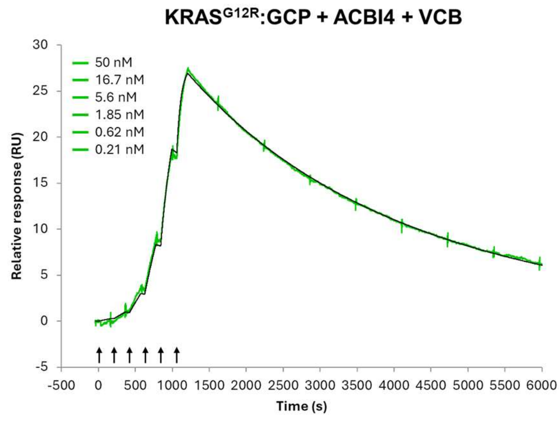
KRAS^{G12R}:GCP + ACBI4 + VCB
50 nM
16.7 nM
5.6 nM
1.85 nM
0.62 nM
0.21 nM
Relative response (RU)
Time (s)
30 25 20 15 10 5 0 -5
-500 0 500 1000 1500 2000 2500 3000 3500 4000 4500 5000 5500 6000

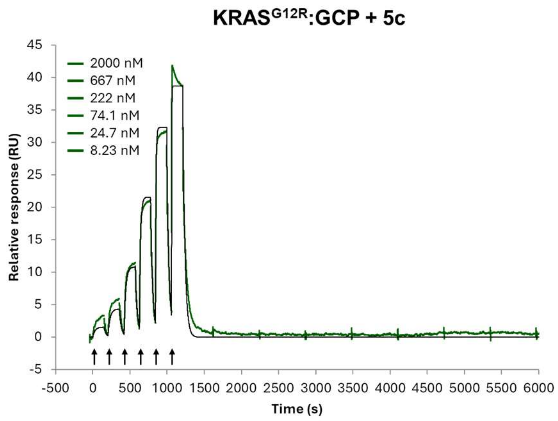
KRAS^G12R:GCP + 5c
2000 nM
667 nM
222 nM
74.1 nM
24.7 nM
8.23 nM
Relative response (RU)
Time (s)

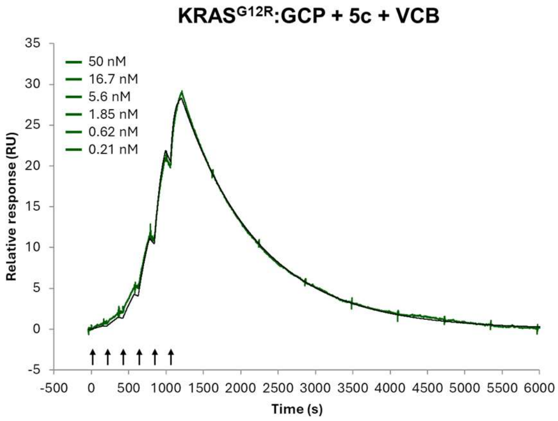
KRAS^G12R:GCP + 5c + VCB
50 nM
16.7 nM
5.6 nM
1.85 nM
0.62 nM
0.21 nM
Relative response (RU)
Time (s)

Figure S4: SPR characterization of ternary complexes for immobilized VCB with KRAS^{G12D}-GCP or KRAS^{G12D}-GDP

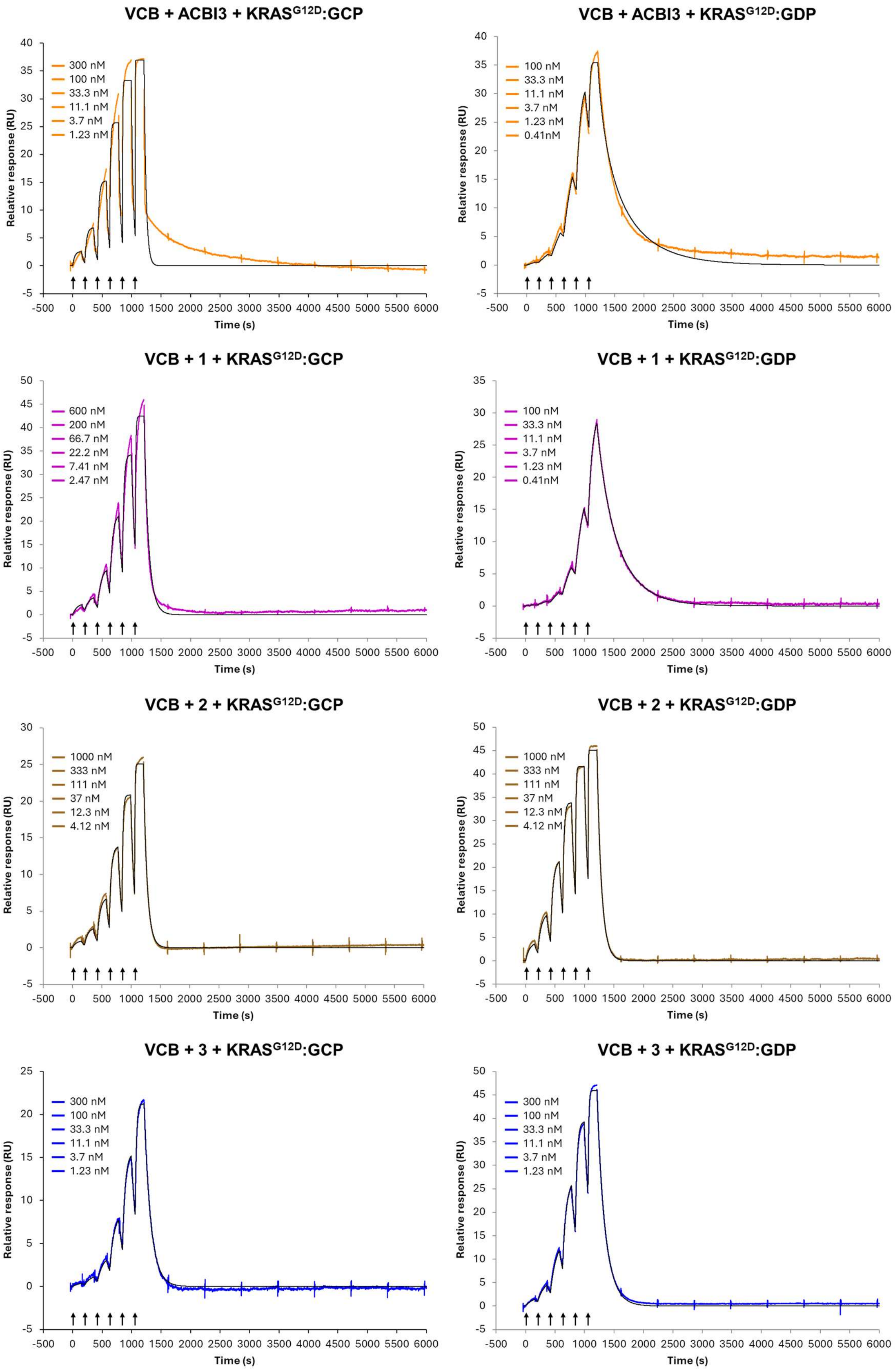

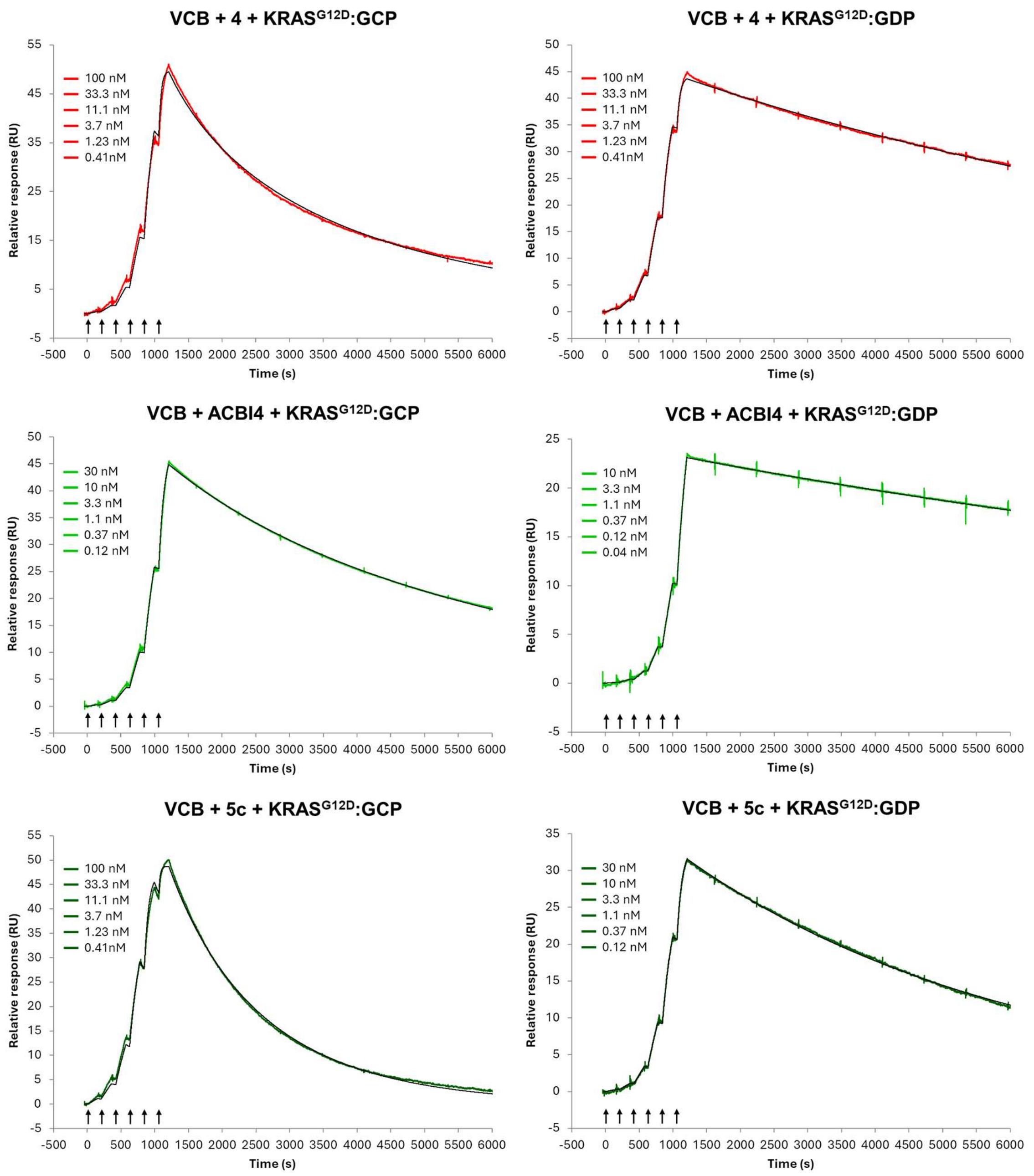

Table S2. Comparison of KRAS^{G12X}-GDP Binary SPR Affinity for BI-2865, ACBI3 and PROTAC **1**

		**KRAS^{G12X}-GDP**			
		KRAS^{WT}	KRAS^{G12D}	KRAS^{G12V}	KRAS^{G12R}
**BI-2865**	***K_D* (nM)**	3.0±1.0 (4)	7.2±2.2 (5)	3.1±1.2 (9)	2.6±0.4 (4)
	t1/2 (s)	0.041±0.011	0.099±0.107	1.164±3.352	0.015±0.018
**ACBI3**	***K_D* (nM)**	4.9±2.6 (2)	5.1±1.1 (13)	3.6±0.9 (10)	8.7±5.1 (4)
	t1/2 (s)	0.006±0.004	0.014±0.004	0.011±0.005	0.007±0.003
**1**	***K_D* (nM)**	4.8±0.0 (2)	14.9±17.4 (13)	4.3±2.4(10)	14.0±16.7 (5)
	t1/2 (s)	0.003±0.000	0.006±0.002	0.005±0.002	0.005±0.002

Data were generated using the protocol from Lito *et al.*[8] *Data for BI-2865 were previously reported.*[8] Errors are ± standard deviation with repeats (N) specified in brackets.

Figure S5. Comparison of SPR Afffinities for PROTACs with KRAS^{G12D}-GCP±VCB and KRAS^{G12R}-GCP±VCB

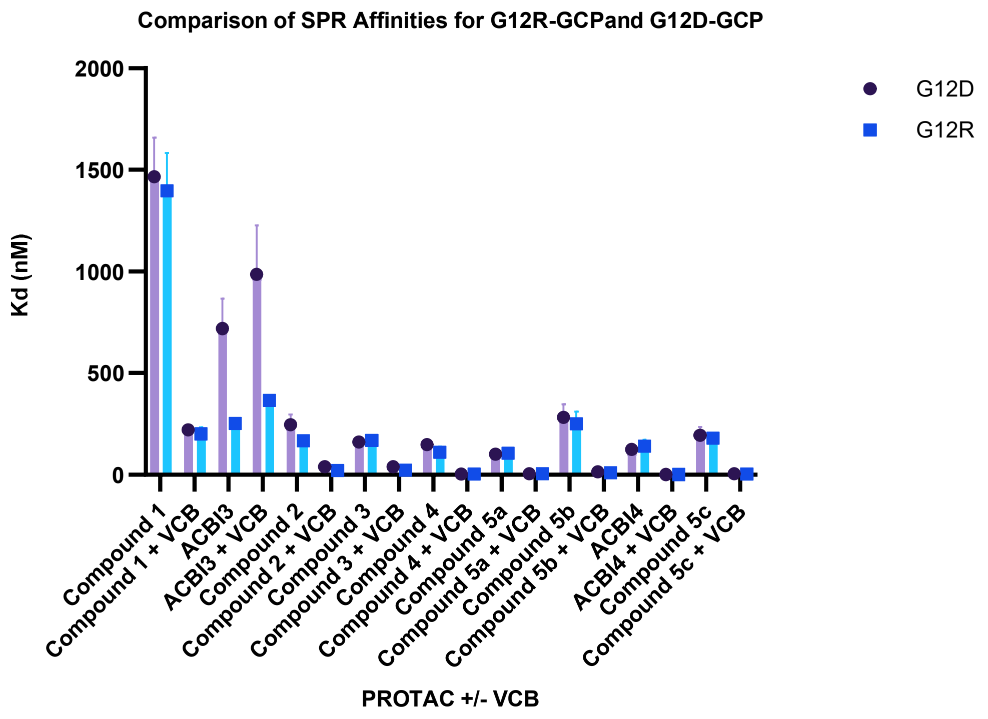

Figure S6. Comparison of Half-Lives for PROTACs with KRAS^{G12D}-GCP±VCB and KRAS^{G12R}-GCP±VCB

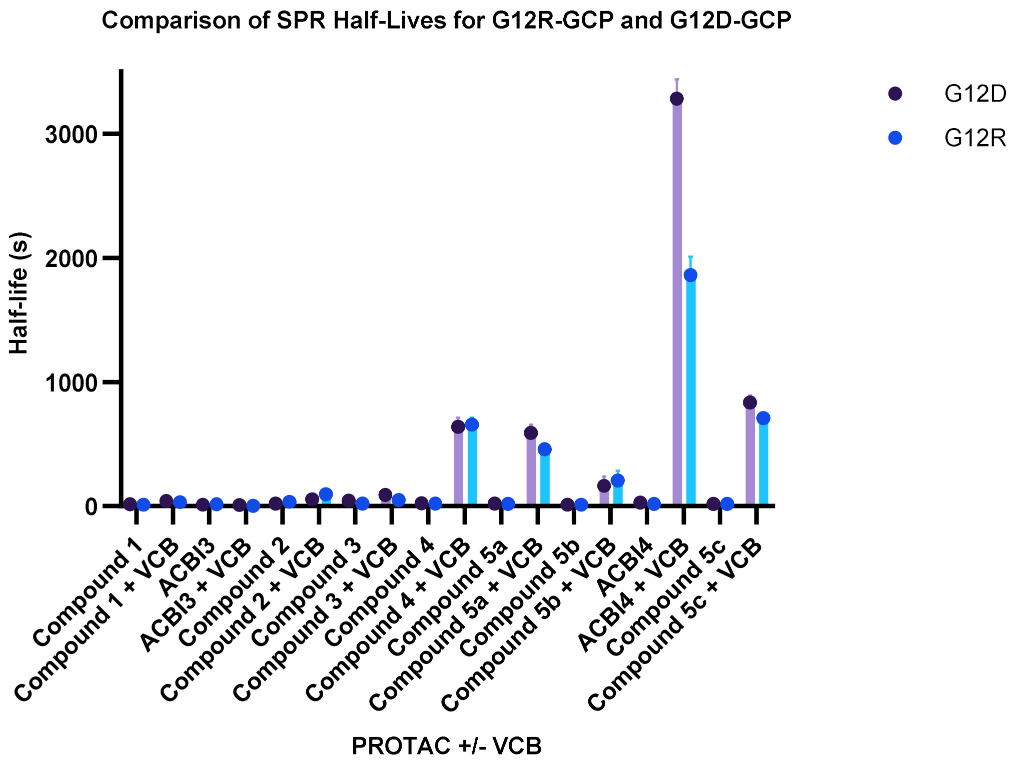

Table S3a. Data Summary *in vitro KRAS* Degradation and Proliferation

	Cell line	1	ACBI3	3	2	4	5a	5b	ACBI4	5c
Dc$_{50}$ 4h (nM)	Gp5D (G12D)	12*	7.3*	36*	n.d.	68*	n.d.	n.d.	7*	n.d.
	Cal-62 (G12R)	unstable	unstable	2209	n.d.	1531	n.d.	n.d.	382	n.d.
	KP-2 (G12R)	unstable	unstable	78	n.d.	6	n.d.	n.d.	316	n.d.
	Calu-6 (Q61K)	unstable	unstable	unstable	n.d.	146	n.d.	n.d.	274	n.d.
	SW948 (Q61L)	unstable	unstable	unstable	n.d.	unstable	n.d.	n.d.	300^	n.d.
	Gp5D (HiBit-G12D)	12.6*	9.9*	61.6*	72*	14*	186.5*	108*	9*	9*
	Cal-62 (HiBitG12R)	80	unstable	188	582	163	1999	979	80	131
Dc$_{50}$ 18h (nM)	Gp5D (G12D)	10	24	6*	n.d.	16	n.d.	n.d.	7	n.d.
	Cal-62 (G12R)	108	282	462	n.d.	418	n.d.	n.d.	162	n.d.
	KP-2 (G12R)	44	961	160	n.d.	224	n.d.	n.d.	151	n.d.
	Calu-6 (Q61K)	unstable	unstable	unstable	n.d.	187	n.d.	n.d.	157	n.d.
	SW948 (Q61L)	unstable	10000*	unstable	n.d.	unstable	n.d.	n.d.	1000^	n.d.
Dc$_{50}$ 24h (nM)	Gp5D (HiBit-G12D)	5.8	2.5	37	25	4	52	23	2	2
	Cal-62 (HiBitG12R)	242	114	483	516	183	1705	838	205	154
DMax 4h (%)	Gp5D (G12D)	95.9	98.3	67	n.d.	83.5	n.d.	n.d.	96.5	n.d.
	Cal-62 (G12R)	21	unstable	69	n.d.	64	n.d.	n.d.	88	n.d.
	KP-2 (G12R)	unstable	unstable	34	n.d.	37	n.d.	n.d.	91	n.d.
	Calu-6 (Q61K)	33	11	unstable	n.d.	25	n.d.	n.d.	34	n.d.
	SW948 (Q61L)	28	unstable	38	n.d.	49	n.d.	n.d.	58	n.d.
	Gp5D (HiBit-G12D)	80	79	54	61	83	66	79	84	80
	Cal-62 (HiBitG12R)	13	unstable	21	41	76	57	70	80	68
DMax 18h (%)	Gp5D (G12D)	99	100	98	n.d.	96.5	n.d.	n.d.	99	n.d.
	Cal-62 (G12R)	50	9	75	n.d.	93	n.d.	n.d.	93	n.d.
	KP-2 (G12R)	63	50	60	n.d.	70	n.d.	n.d.	82	n.d.
	Calu-6 (Q61K)	27	unstable	31	n.d.	72	n.d.	n.d.	68	n.d.
	SW948 (Q61L)	25	45^	45	n.d.	50	n.d.	n.d.	65^	n.d.
DMax 24h (%)	Gp5D (HiBit-G12D)	94	94	92	90	94	90	91	93	93
	Cal-62 (HiBitG12R)	64	31	69	69	87	73	89	87	82
CTG 72h Ic$_{50}$ (nM)	Cal-62 (G12R)	Unstable	unstable	1583	n.d.	488	n.d.	n.d.	174	n.d.
	Calu-6 (Q61K)	unstable	338	1000	n.d.	unstable	n.d.	n.d.	unstable	n.d.
CTG 144h Ic$_{50}$ (µM)	A375 (KRAS WT; KRAS independent)	>3**	n.d.	>3	n.d.	n.d.	n.d.	n.d.	>3	n.d.

Data were performed in triplicate; n.d. = not determined; unstable = no degradation/poor curve shape; *Hook effect was removed before calculating DC$_{50;}$ ^Data points were manually derived; **Data were previously reported[4]

Table S3b. Data Summary Kinetic Degradation

	Cell line	1	ACBI3	3	4	ACBI4
Plateau [1/min]	Cal62-HiBit	0.4749	unstable	0.1779	0.2614	0.2774
1/K [µM]	(G12R)	18	unstable	2.336	1.972	0.689
Plateu/ Tau [1/(M s)]	Live mode	439	unstable	1269	2207	6708

Data were plotted from n=5 independent experiments; unstable = no degradation; Tau=1/K

Figure S7: Effect of KRAS^{G12R} Depletion in Cell Lines of Interest

SiRNA depletion of KRAS levels in cell lines bearing KRAS^{G12R} mutations

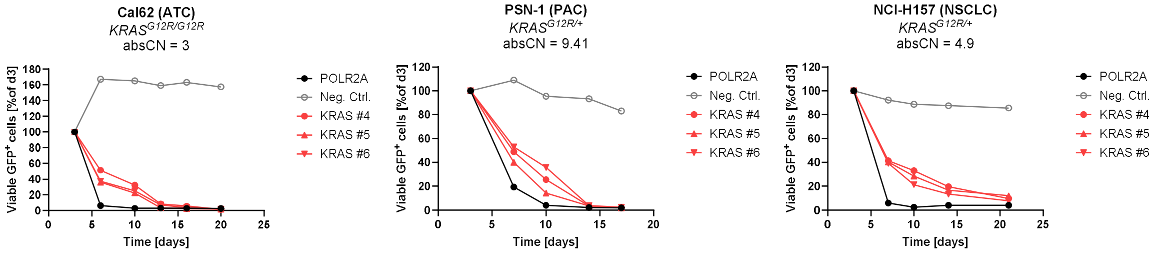

Depletion of KRAS levels by compound **3** in cell lines bearing KRAS^{G12R} mutations; 6 day CTG assay

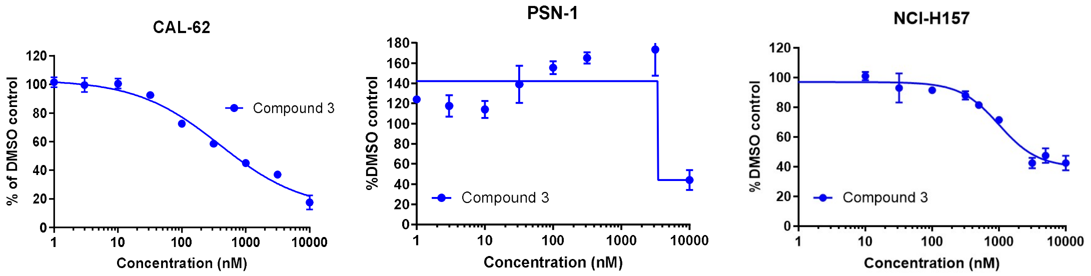

Figure S8: Whole Cell Proteomic Analysis

Whole cell proteomics MS analysis of Cal-62 cells treated for 6 h with 1 µM ACBI4 compared to inactive negative control or DMSO. KRAS, HRAS and NRAS are highlighted in pink

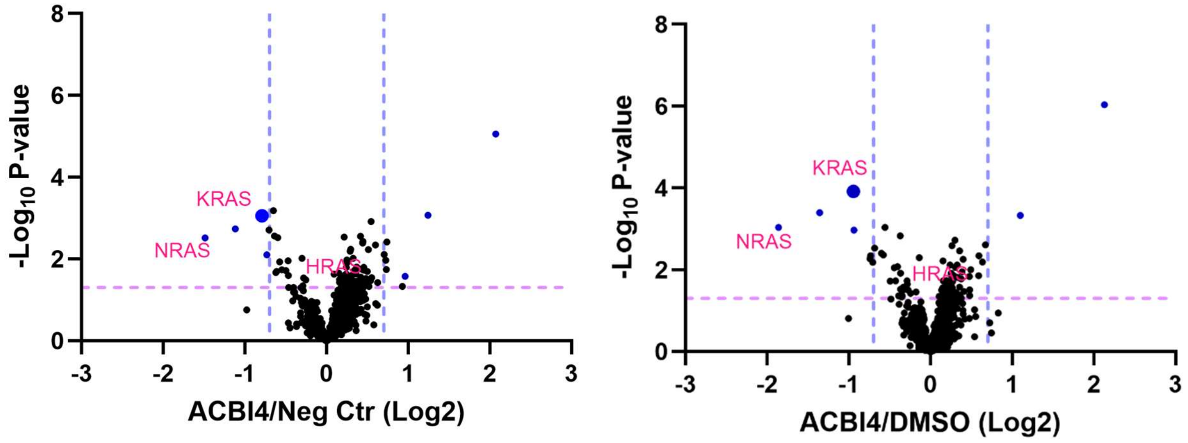

Table S4: Significantly Up- and Down-regulated Proteins in Whole Cell Proteomics Analysis

ACBI4/DMSO comparison and ACBI4/negative control comparison

T-test ACBI4/DMSO FDR<0.05			T-test ACBI4/Neg Ctr FDR<0.05		
**Protein**	**p-value**	**Log2 Difference**	**Protein**	**p-value**	**Log2 Difference**
KRAS	3.91	-0.94	KRAS	3.05	-0.79
G0S2	3.39	-1.35	G0S2	2.73	-1.11
NRAS	3.03	-1.86	FOSL1	2.7	-0.7
FOSL1	2.97	-0.93	NRAS	2.51	-1.48
CDKN1C	3.32	1.09	PLAU	2.1	-0.73
RHOB	6.03	2.12	TSPYL4	1.57	0.96
			CDKN1C	3.06	1.24
			RHOB	5.05	2.07

Figure S9. Endogenous Evaluation of the Effect of ACBI4 on HRAS and NRAS levels in Cal-62 Cells

Data from western Blot analysis of HRAS levels in Cal-62 cells treated with ACBI4 for 4 h (*N*=2 biological replicates, SD)

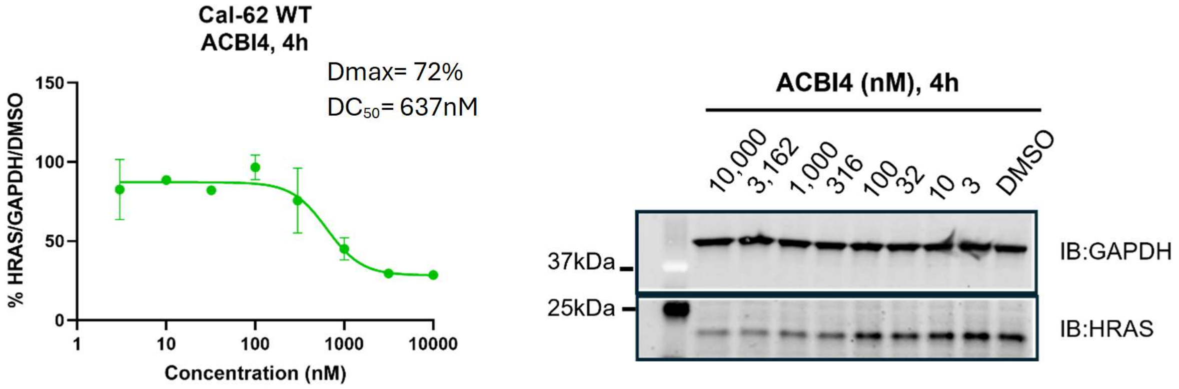

Data from western Blot analysis of NRAS levels in Cal-62 cells treated with ACBI4 for 4 h (*N*=2 biological replicates, SD)

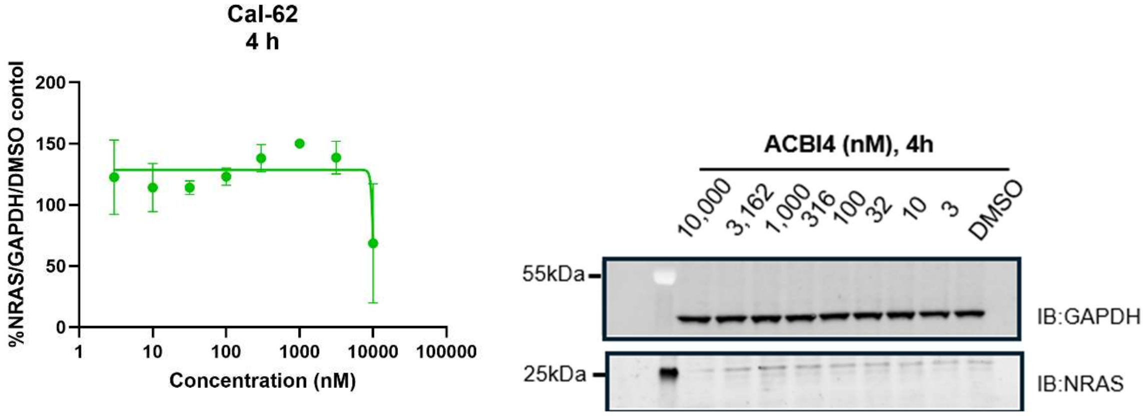

Data from western Blot analysis of NRAS levels in Cal-62 cells treated with ACBI4 for 18 h (*N*=2 biological replicates, SD)

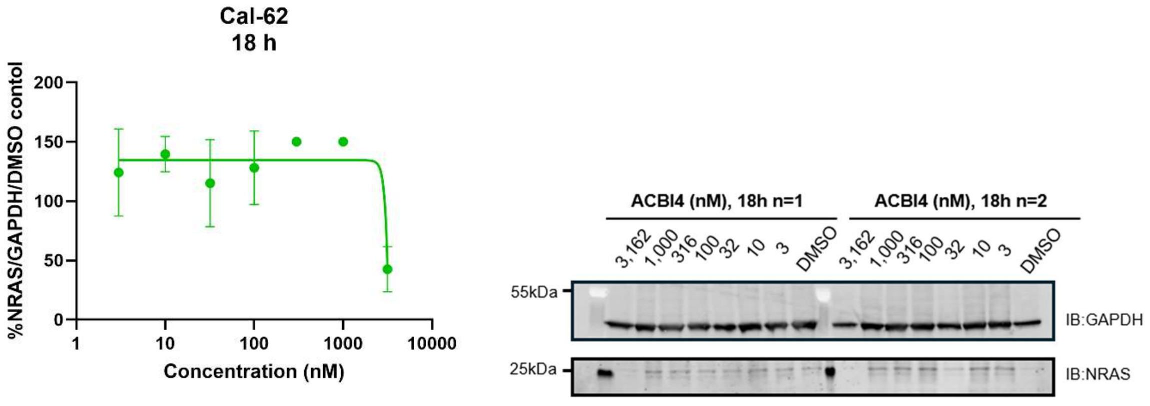

Figure S10. KRAS Degradation in KP2 Cells

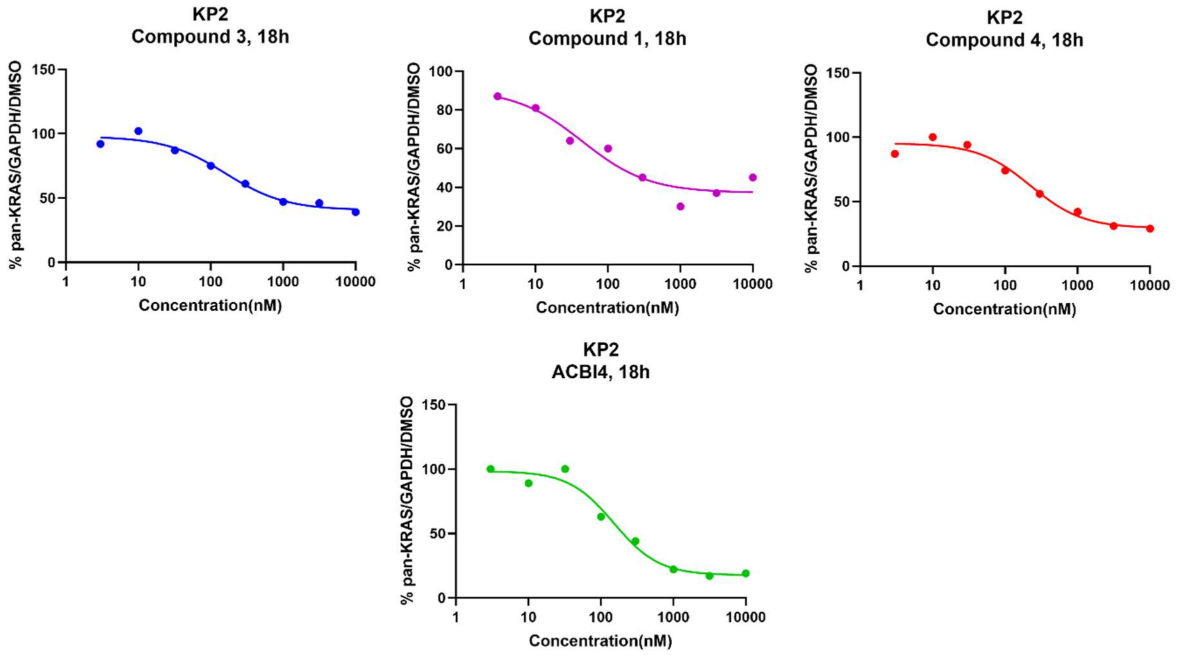

Figure S11. Representative Western Blot from MoA Experiment in Cal-62 Cells for ACBI4

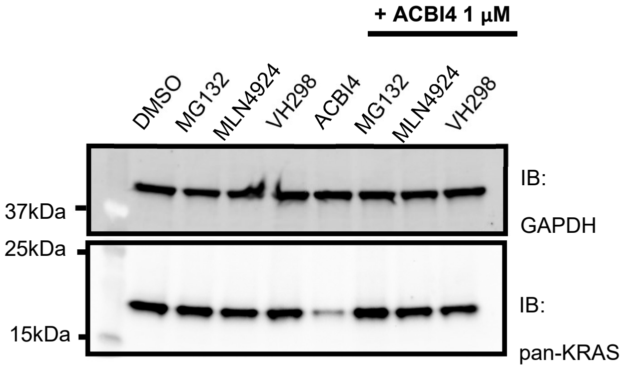

Figure S12. CTG Proliferation Data for ACBI4 in A375 Cell Line

No antiproliferation effect was observed for ACBI4 in the KRAS independent A375 cell line (*N*=4 biological replicates, SD)

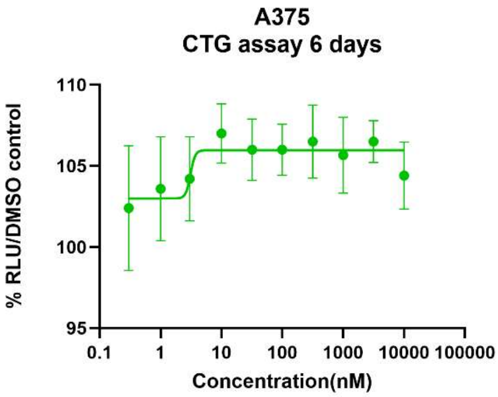

Table S5. Crystallographic Data and Refinement Statistics

	KRAS^{G12R}-GDP:1:VCB (PDB: 9RKE)	KRAS^{G12V}-GDP:3:VCB (PDB: 9RK8)	KRAS^{G12R}-GCP:3:VCB (PDB: 9RKJ)	KRAS^{G12D}-GDP:ACBI4:VCB (PDB: 9RKC)	KRAS^{G12R}-GCP:ACBI4:VCB (PDB: 9RKN)
**Resolution range (Å^2)**	91.89 - 2.83 (2.931 - 2.83)	54.71 - 2.63 (2.724 - 2.63)	79.5 - 2.89 (2.994 - 2.89)	44.81 - 2.19 (2.273 - 2.19)	58.05 - 2.85 (2.952 - 2.85)
**Space group**	P 32 2 1	C 2 2 21	P 21 21 21	P 1 21 1	P 1 21 1
**a, b, c (Å)**	101.9, 101.9, 275.7	96.2, 109.4, 252.0	101.6, 110.6, 114.4	71.9, 106.5, 82.7	72.3, 122.1, 80.3
**α, β, γ (°)**	90, 90, 120	90, 90, 90	90, 90, 90	90, 108.7, 90	90, 114.1, 90
**Total reflections**	1247082 (121054)	402589 (41932)	38494 (518)	428055 (42142)	210307 (20794)
**Unique reflections**	40611 (3958)	39396 (190)	19250 (259)	59951 (5834)	29820 (2920)
**Multiplicity**	30.7 (30.6)	10.2 (10.6)	2.0 (2.0)	7.1 (7.3)	7.1 (7.1)
**Completeness (%)**	99.5 (96.54)	72.79 (4.81)	65.03 (8.93)	97.6 (82.43)	99.61 (76.34)
**Mean I/sigma(I)**	4.9 (0.43)	9.12 (0.46)	7.30 (1.22)	4.1 (0.3)	3.2 (0.36)
**Wilson B-factor (Å^2)**	73.97	57.32	50.55	45.12	59.63
**R-merge**	0.452 (4.536)	0.197 (4.207)	0.109 (0.573)	0.272 (2.694)	0.353 (2.614)
**R-meas**	0.459 (4.613)	0.208 (4.418)	0.154 (0.810)	0.293 (2.903)	0.381 (2.819)
**CC1/2**	0.973 (0.309)	0.998 (0.217)	0.977 (0.538)	0.989 (0.318)	0.981 (0.302)
**Reflections used in refinement**	40324 (3821)	29071 (190)	19184 (259)	59000(4959)	29088 (2255)
**Reflections used for R-free**	2004 (186)	1411 (4)	932 (10)	1957 (167)	1524 (128)
**R-work**	0.234 (0.369)	0.232 (0.293)	0.237 (0.439)	0.232 (0.393)	0.226 (0.377)
**R-free**	0.286 (0.397)	0.263 (0.376)	0.287 (0.511)	0.274 (0.427)	0.275 (0.392)
**Number of non-hydrogen atoms**	7771	7992	7708	8044	7907
macromolecules	7527	7842	7453	7854	7684
ligands	238	146	242	142	221
solvent	6	4	13	48	2
Protein residues	994	998	982	995	993
**RMS(bonds)**	0.003	0.004	0.002	0.004	0.002
**RMS(angles)**	0.54	0.76	0.48	0.76	0.59
**Ramachandran favoured (%)**	95.87	96.71	96.13	97.12	96.06
**Ramachandran allowed (%)**	3.93	3.09	3.66	2.68	3.73
**Ramachandran outliers (%)**	0.21	0.21	0.21	0.21	0.21
**Rotamer outliers (%)**	1.95	3.81	2.21	0.59	2.2
**Clashscore**	5.62	7.84	5.12	5.71	5.73
**Average B-factor**	89.15	66.59	47.47	55.97	65.55
macromolecules	89.64	66.91	47.54	56.35	65.64
ligands	74.18	45.84	46.75	43.47	62.51
solvent	62.88	52.54	23.17	41.82	37.77

Figure S13. Modelling of ACBI4 Isomers

Overlay of ACBI4 modelled with the isopropyl stereocentre in the (*R*)-configuration (yellow) and in the (*S*)-configuration (purple) within the Omit Fo-Fc map (green) shown contoured at 3σ from the ternary crystal structure KRAS^{G12D}-GDP:ACBI4:VCB.

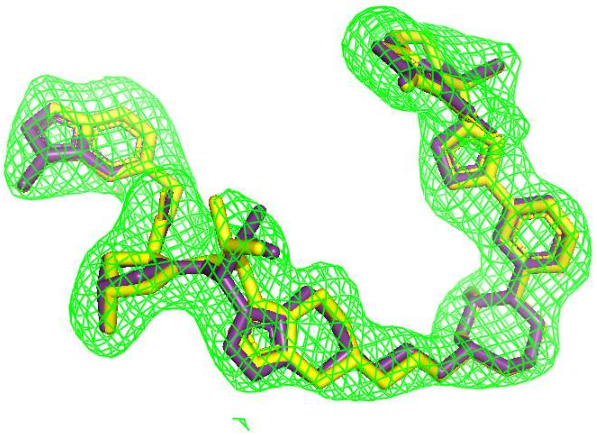

	***i*Pr Stereocentre Configuration**			
	R		***S***	
	**SCORE**	**CC**	**SCORE**	**CC**
**Ligand Copy 1**	138.29	0.858	136.59	0.889
**Ligand Copy 2**	146.02	0.848	139.97	0.903

Overlay of ACBI4 modelled with the isopropyl stereocentre in the (*R*)-configuration (yellow) and in the (*S*)-configuration (purple within the Omit Fo-Fc map (green) shown contoured at 3σ from the ternary crystal structure KRAS^{G12R}-GCP:ACBI4:VCB.

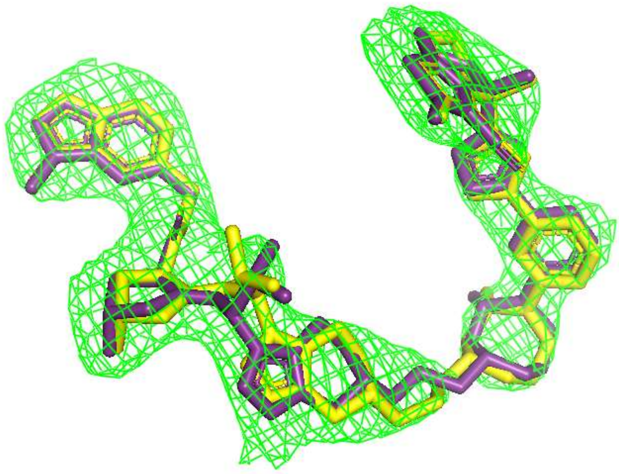

	***i*Pr Stereocentre Configuration**			
	R		***S***	
	**SCORE**	**CC**	**SCORE**	**CC**
**Ligand Copy 1**	131.36	0.856	132.51	0.877
**Ligand Copy 2**	126.67	0.814	127.9	0.858

References

(1) Farnaby, W.; Koegl, M.; Roy, M. J.; Whitworth, C.; Diers, E.; Trainor, N.; Zollman, D.; Steurer, S.; Karolyi-Oezguer, J.; Riedmueller, C.; et al. BAF complex vulnerabilities in cancer demonstrated via structure-based PROTAC design. *Nature Chemical Biology* **2019**, *15* (7), 672–680. DOI: 10.1038/s41589-019-0294-6.

(2) Gadd, M. S.; Testa, A.; Lucas, X.; Chan, K.-H.; Chen, W.; Lamont, D. J.; Zengerle, M.; Ciulli, A. Structural basis of PROTAC cooperative recognition for selective protein degradation. *Nature Chemical Biology* **2017**, *13* (5), 514–521. DOI: 10.1038/nchembio.2329.

(3) Kessler, D.; Gmachl, M.; Mantoulidis, A.; Martin, L. J.; Zoephel, A.; Mayer, M.; Gollner, A.; Covini, D.; Fischer, S.; Gerstberger, T.; et al. Drugging an undruggable pocket on KRAS. *Proceedings of the National Academy of Sciences* **2019**, *116* (32), 15823–15829. DOI: doi:10.1073/pnas.1904529116.

(4) Popow, J.; Farnaby, W.; Gollner, A.; Kofink, C.; Fischer, G.; Wurm, M.; Zollman, D.; Wijaya, A.; Mischerikow, N.; Hasenoehrl, C.; et al. Targeting cancer with small-molecule pan-KRAS degraders. *Science* **2024**, *385* (6715), 1338–1347. DOI: doi:10.1126/science.adm8684.

(5) Köferle, A.; Schlattl, A.; Hörmann, A.; Thatikonda, V.; Popa, A.; Spreitzer, F.; Ravichandran, M. C.; Supper, V.; Oberndorfer, S.; Puchner, T.; et al. Interrogation of cancer gene dependencies reveals paralog interactions of autosome and sex chromosome-encoded genes. *Cell Reports* **2022**, *39* (2), 110636. DOI: 10.1016/j.celrep.2022.110636.

(6) Cox, J.; Mann, M. MaxQuant enables high peptide identification rates, individualized p.p.b.-range mass accuracies and proteome-wide protein quantification. *Nature Biotechnology* **2008**, *26* (12), 1367–1372. DOI: 10.1038/nbt.1511.

(7) Tyanova, S.; Temu, T.; Sinitcyn, P.; Carlson, A.; Hein, M. Y.; Geiger, T.; Mann, M.; Cox, J. The Perseus computational platform for comprehensive analysis of (prote)omics data. *Nature Methods* **2016**, *13* (9), 731–740. DOI: 10.1038/nmeth.3901.

(8) Kim, D.; Herdeis, L.; Rudolph, D.; Zhao, Y.; Böttcher, J.; Vides, A.; Ayala-Santos, C. I.; Pourfarjam, Y.; Cuevas-Navarro, A.; Xue, J. Y.; et al. Pan-KRAS inhibitor disables oncogenic signalling and tumour growth. *Nature* **2023**, *619* (7968), 160–166. DOI: 10.1038/s41586-023-06123-3.
